# A Koopman operator-based prediction algorithm and its application to COVID-19 pandemic and influenza cases

**DOI:** 10.1038/s41598-024-55798-9

**Published:** 2024-03-09

**Authors:** Igor Mezić, Zlatko Drmač, Nelida Črnjarić, Senka Maćešić, Maria Fonoberova, Ryan Mohr, Allan M. Avila, Iva Manojlović, Aleksandr Andrejčuk

**Affiliations:** 1 University of California, Santa Barbara, CA 93106 USA; 2 Faculty of Science, University of Zagreb, Zagreb, Croatia; 3 Faculty of Engineering, University of Rijeka, Rijeka, Croatia; 4 AIMdyn Inc., Santa Barbara, CA 93101 USA; 5 Department of Applied Mathematics, Faculty of El. Engineering, University of Zagreb, Zagreb, Croatia

**Keywords:** Koopman operator, Prediction theory, COVID-19, Applied mathematics, Influenza virus, Viral infection, Applied mathematics, Influenza virus, Viral infection

## Abstract

Future state prediction for nonlinear dynamical systems is a challenging task. Classical prediction theory is based on a, typically long, sequence of prior observations and is rooted in assumptions on statistical stationarity of the underlying stochastic process. These algorithms have trouble predicting chaotic dynamics, “Black Swans” (events which have never previously been seen in the observed data), or systems where the underlying driving process fundamentally changes. In this paper we develop (1) a global and local prediction algorithm that can handle these types of systems, (2) a method of switching between local and global prediction, and (3) a retouching method that tracks what predictions would have been if the underlying dynamics had not changed and uses these predictions when the underlying process reverts back to the original dynamics. The methodology is rooted in Koopman operator theory from dynamical systems. An advantage is that it is model-free, purely data-driven and adapts organically to changes in the system. While we showcase the algorithms on predicting the number of infected cases for COVID-19 and influenza cases, we emphasize that this is a general prediction methodology that has applications far outside of epidemiology.

## Introduction

Ability for prediction of events is one of the key differentiators of homo sapiens. The key element of prediction is reliance on collected data over some time interval for estimation of evolution over the next time period. Mathematicians have long worked on formal aspects of prediction theory, and separate streaks such as the Wiener–Kolmogorov,^1^, Furstenberg^2^ and Bayesian prediction^3^ have emerged. However, all of these are concerned with prediction of future events based on a, typically long, sequence of prior observations. This is rooted in assumptions on statistical stationarity of the underlying stochastic process.

Furthermore, classical methods have difficulty in predicting chaotic systems due to their sensitivity to initial conditions leading to large divergence of initially close-by initial conditions (“Butterfly effect”). There have been some work in the machine learning literature that seek to make arbitrarily long prediction, such as Fan et. al.^4^. In that paper, the authors combine reservoir computing systems with an infrequent data assimilation step to extend the prediction window past one Lyapunov time. However, the paper considers predictions models for single systems, assuming they do not change, and do not consider the case where the underlying dynamics can fundamentally change.

In contrast to the Butterfly effect, which is an inherent property of some nonlinear, deterministic dynamical systems, another difficulty for classical prediction algorithms is a “Black Swan” event (a hard-to-predict and rare event beyond the realm of normal expectations) or in the dynamical context a sudden fundamental change in the underlying driving process. For typical learning algorithms these type of events are devastating; the learning algorithm has to be restarted as otherwise it would learn the deviation as normal.

This paper develops a model-free, purely data-driven prediction algorithm that can handle both the “Butterfly” effects and “Black Swan” events. The point of view on prediction in this paper is quite different: we view the process over a short (local) time scale and extract its coarse-grained ingredients. We proceed with prediction of the evolution based on these, learning the process and building a global time-scale on which such prediction is valid. Then, we monitor for  the change in such coarse-grained ingredients, detect if a substantial change is happening, and switch back to local learning and prediction. In this way, we accept the limitations on predictability due to, possibly finite time, nonstationarity, and incorporate them into the prediction strategy.

The developed algorithm is rooted in Koopman operator theory^5–10^ in its recently developed form that is applicable to nonstationary stochastic processes^11,12^. The Koopman operator theory is predicated on existence of a composition operator that dynamically evolves all the possible observables on the data, enabling the study of nonlinear dynamics by examining its action on a linear space of observables. The key ingredients of this approach become eigenvalues and eigenfunctions of the Koopman operator and the associated Koopman Mode Decomposition (KMD) of the observable functions, which is then approximated numerically using Dynamic Mode Decomposition (DMD). The numerical approach used in this work relies on lifting the available data to higher dimensional space using Hankel–Takens matrix and on the improved implementation of DMD algorithm for discovering the approximations of the Koopman modes with small residuals. The obtained Koopman mode approximations and the related eigenvalues, called Ritz pairs, are crucial for obtaining satisfactory predictions using KMD.

The contributions of this paper are three-fold: (1) Development of purely data-driven global and local prediction algorithms, (2) a method of switching between the two, and (3) a “retouching” algorithm that tracks what predictions would have been if the underlying dynamics had not changed and uses these predictions when the underlying process reverts back to the original dynamics. While we show the application of the methods on epidemiology examples (e.g. predicting COVID-19 number of infected) in the main text, we emphasize that this is a general method with applications well outside of epidemiology. We refer the reader to the Supplementary Information for mathematical details and additional examples.

## Methods

Our starting assumption is that observed data is generated by a dynamical process realized on some underlying state space. This is a broad enough assumption to cover data generated by both deterministic and stochastic dynamical systems^9^. The (internal) state is often inaccessible; instead, an observable (output) is given as a function $f({\textbf{x}}(t))$ of the state vector ${\textbf{x}}(t)$.

### The Koopman operator and the KMD

The Koopman operator family ${\mathscr {U}}^t$, acts on observables *f* by composition ${\mathscr {U}}^t f ({\textbf{x}}) = f({\textbf{x}}( t))$. It is a global linearization tool: ${\mathscr {U}}^t$ is a linear operator that allows studying the nonlinear dynamics by examining its action on a linear space ${\mathscr {F}}$ of observables. In data analysis, for the discrete time steps $t_i$, the discrete sequence ${\textbf{z}}_i\approx {\textbf{x}}(t_i)$, generated as numerical software output, is then a discrete dynamical system ${\textbf{z}}_{i+1}={\textbf{T}}({\textbf{z}}_i)$, for which the Koopman operator reads ${\mathscr {U}}f = f\circ {\textbf{T}}$.

The key of the spectral analysis of the dynamical system is a representation of a vector valued observable ${\textbf{f}}=(f_1,\ldots ,f_{d})^T$ as a linear combination of the eigenfunctions ${\varvec{\psi }}_j$ of ${\mathscr {U}}$. In a subspace spanned by eigenfunctions each observable $f_i$ can be written as $f_i({\textbf{z}}) \approx \sum _{j=1}^{\infty }{\varvec{\psi }}_j({\textbf{z}}) ({\textbf{v}}_j)_i$ and thus (see e.g.^6,13^)

1 $$\begin{aligned} \!{\textbf{f}}({\textbf{z}}) = \!\left( {\begin{matrix} f_1({\textbf{z}}) \\ \vdots \\ f_{d}({\textbf{z}})\end{matrix}}\right) \approx \sum _{j=1}^{\infty } {\varvec{\psi }}_j({\textbf{z}}) {\textbf{v}}_j,\; \text{ where }\;\; {\textbf{v}}_j =\! \left( {\begin{matrix} ({\textbf{v}}_j)_1 \\ \vdots \\ ({\textbf{v}}_j)_{d}\end{matrix}}\right) \!\!, \end{aligned}$$

then, since ${\mathscr {U}}{\varvec{\psi }}_j=\lambda _j{\varvec{\psi }}_j$, we can envisage the values of the observable ${\textbf{f}}$ at the *future* states ${\textbf{T}}({\textbf{z}})$, ${\textbf{T}}^2({\textbf{z}}), \ldots$ by

2 $$\begin{aligned} ({\mathscr {U}}^k{\textbf{f}})({\textbf{z}}) {\mathop {=}\limits ^{\mathrm {\tiny def}}} {\textbf{f}}({\textbf{T}}^k({\textbf{z}}))\approx \sum _{j=1}^{\infty } \lambda _j^k {\varvec{\psi }}_j({\textbf{z}}) {\textbf{v}}_j, \;\;k=1,2,\ldots \end{aligned}$$

The numerical approximation of KMD can be computed using for example DMD algorithms. Different versions of the algorithm used in this work are described in details in Supporting Information-Methods.

### Finite dimensional compression and Rayleigh–Ritz extraction

For practical computation, ${\mathscr {U}}$ is restricted to a finite dimensional space ${\mathscr {F}}_{{\mathscr {D}}}$ spanned by the dictionary of suitably chosen functions ${\mathscr {D}}=\{ f_1, \ldots , f_d\}$, and we use a matrix representation ${\mathbb {U}}$ of the compression ${\varvec{\Psi }}_{{\mathscr {F}}_{{\mathscr {D}}}}{\mathscr {U}}_{|{\mathscr {F}}_{{\mathscr {D}}}} : {\mathscr {F}}_{{\mathscr {D}}}\rightarrow {\mathscr {F}}_{{\mathscr {D}}}$, where ${\varvec{\Psi }}_{{\mathscr {F}}_{{\mathscr {D}}}}$ is a $L^2$ projection e.g. with respect to the empirical measure defined as the sum of the Dirac measures concentrated at the ${\textbf{z}}_i$’s. Since ${\mathbb {U}}$ is the adjoint of the DMD matrix ${\mathbb {A}}$ associated with the snapshots ${\textbf{z}}_i$, the approximate (numerical) Koopman modes and the eigenvalues are the Ritz pairs (Ritz eigenvalues and eigenvectors) of ${\mathbb {A}}$, computed using the Rayleigh–Ritz method. The residuals of the Ritz pairs can be computed and used to check the accuracy^14^. See Supporting Information-Methods.

### The Hankel-DMD (H-DMD)

The data snapshots (numerical values of the observables) can be rearranged in a Hankel–Takens matrix structure: for a subsequence (*window*) of ${\textsf{w}}$ successive snapshots ${\textbf{f}}_b, {\textbf{f}}_{b+1}, \ldots , {\textbf{f}}_{{\textsf{w}}-1}$, split ${\textsf{w}}= m_H + n_H$ and then define new snapshots as the columns ${\textbf{h}}_i$ of the $n_H\times m_H$ Hankel–Takens matrix (see^15–17^, and Supporting Information)

3 $$\begin{aligned} {\mathbb {H}}= \!\! \begin{pmatrix} {\textbf{f}}_{b} &{} {\textbf{f}}_{b+1} &{} \cdots &{} {\textbf{f}}_{b+m_H} \\ {\textbf{f}}_{b+1} &{} {\textbf{f}}_{b+2} &{} \cdots &{} {\textbf{f}}_{b+m_H+1} \\ \vdots &{} \vdots &{} \ddots &{} \vdots \\ {\textbf{f}}_{b+n_H-1} &{} {\textbf{f}}_{b+n_H} &{} \cdots &{} {\textbf{f}}_{b+n_H+m_H-1} \\ \end{pmatrix} \!= \!\begin{pmatrix} {\textbf{h}}_1&\ldots&{\textbf{h}}_{m_H+1} \end{pmatrix} \!. \end{aligned}$$

Then, for this data we compute the KMD and use (2) for prediction. Predictions of the observables ${\textbf{f}}_i$ are then extracted from the predicted values of the observables ${\textbf{h}}_i$.

The introduction of Hankel–Takens matrix alleviates issues that arise from using a basis on a potentially high dimensional space: namely, taking products of basis elements on 1-dimensional subspaces—for example Fourier basis on an interval in ${\mathbb {R}}$. Such constructions lead to an exponential growth in the number of basis elements, and the so-called curse of dimensionality. The Hankel–Takens matrix is based on the dynamical evolution of a one or more observables—functions on state space—that span a Krylov subspace. The idea is that one might start even with a single observable, and due to its evolution span an invariant subspace of the Koopman operator (note the connection of such methods with the Takens embedding theorem ideas^16–18^). Since the number of basis elements is in this case equal to the number of dynamical evolution steps, in any dimension, Krylov subspace-based methods do not suffer from the curse of dimensionality.

### Global/local Koopman prediction and “Black Swan” detection

We briefly describe at a high level the Global Koopman Prediction algorithm (GKP), detection of “Black Swan” events, and the local prediction algorithm. For full details, we refer the reader to the Supplementary Material (S1.4, S1.7, S1.7.2). We start we Global Prediction algorithm which relies on a sliding window Hankel DMD. Set an active window size *w*. If the present time moment is $t_{p-1}$, take the snapshots $\{{\textbf{f}}_{p-w}, \dots , {\textbf{f}}_{p-1}\}$ and form a Hankel–Takens matrix as in (3). Using an algorithm such as DMD^19^ which returns a set of Ritz pairs $\{\lambda _i, {\textbf{v}}_i\}_{i=1}^n$, and their associated residuals $\{r_i\}$, we can obtain the approximate decomposition of the considered dynamics using a truncated version of (1). If the residuals of the Ritz pairs are small, we can have an accurate decomposition, which can be used for the prediction far out this active window.

Detection of “Black Swan” events or major disturbances to the system are based on the spectral information computed above. In the absence of disturbances, one would expect that the spectral radius for the Ritz values corresponding to the active window would not change too much. Furthermore, the DMD algorithm should compute Ritz pairs with reasonably small residuals. Choosing thresholds ${\mathscr {I}}$ and $\eta$, one can flag the active window as possibly containing a Black Swan event if the spectral radius is greater than ${\mathscr {I}}$ or, alternatively, if all residuals are greater than $\eta$. If the Black Swan event is detected, to successfully predict after it, some retouching process is applied to the data so that original dynamics is decoupled from this disturbance.

In some cases, the global prediction algorithm is not feasible. For instance, when we just start collecting the data, we have not enough information for a GKP analysis. The other situation is when GKP recognizes the beginning of a Black Swan event. In that case, due to the fact that dynamics changed, the available data can not be used for prediction since there will be not enough Ritz pairs with small residuals that can give the accurate decomposition. Thus one can switch to Local prediction algorithm with much smaller active window size. In the Local Koopman Prediction LKP algorithm we change the size of the active window depending on the success of the previous prediction. The idea is to assimilate as much acquired data as possible, so we set Hankel matrix dimension variable with prediction moment. We start with a minimum Hankel size. If the error between the prediction and the actual value are below a certain threshold, we assimilate the newly acquired data into the active window by increasing the size of the Hankel matrix by 1 in each dimension.

## Results

We apply our algorithms to a few case studies in epidemiology: Influence epidemics and COVID-19. We do emphasize that the techniques are general and can be applied to any system that experience a drastic change in its fundamental behavior.

### Case study: influenza epidemics

As first example for showing our prediction methodology, we use the set of data associated with influenza epidemics. Clearly, not driven by an underlying deterministic dynamical system, the influenza time series exhibits substantial regularity in that it occurs typically during the winter months, thus enabling coarse-grained prediction of the type “we will see a very small number of cases of influenza occurring in summer months”. However, predicting the number of influenza cases accurately is a notoriously hard problem^20^, exacerbated by the possibility that a vaccine designed in a particular year does not effectively protect against infection. Moreover, the H1N1 pandemic that occurred in 2009 is an example of a Black Swan event.

The World Health Organization’s FluNet is a global web-based tool for influenza virological surveillance. FluNet makes publicly available data on the number of specimens with the detected influenza viruses of type A and type B. The data have been collected from different countries, starting with the year 1997, and are updated weekly by the National Influenza Centers (NICs) of the Global Influenza Surveillance and Response System (GISRS) and other national influenza reference laboratories, collaborating actively with GISRS. We use the weekly reported data for different countries, which consist of the number of received specimens in the laboratories, the distribution of the number of specimens with confirmed viruses of type  A.

The Koopman Mode Decomposition was used in the context of analyzing the dynamics of the flu epidemic from different—Google Flu—data in^21^. We remark that the authors of that paper have not attempted prediction, and have analyzed only “stationary” modes—e.g. the yearly cycles, thus making the paper’s goals quite different from the nonstationary prediction pursued here.

We first compare the global and the local prediction algorithms. The KMD is computed using active windows of size ${\textsf{w}}= 312$, and the $208 \times 104$ Hankel–Takens matrices. In Fig. reff1a, we show the performances of both algorithms, using the learning data from the window April 2003–April 2009 (shadowed rectangle). In the global prediction algorithm the dynamics is predicted for 104 weeks ahead. The first type of failure in the global prediction algorithm and forecasting appears after the Black Swan event occurred in the years 2009 and 2010. This is recognized by the algorithm, so that it adapts by using the smallest learning span and, with this strategy, it allows for reasonably accurate forecasting, at least for shorter lead times. This data, in addition to those from Supplementary Information section S2.4 show the benefits of monitoring the prediction error and switching to local prediction. The initial Hankel–Takens matrix is $3\times 2$, and the threshold for the local prediction relative error in Supplementary Information Algorithm S4 is 0.005.

**Figure 1:**
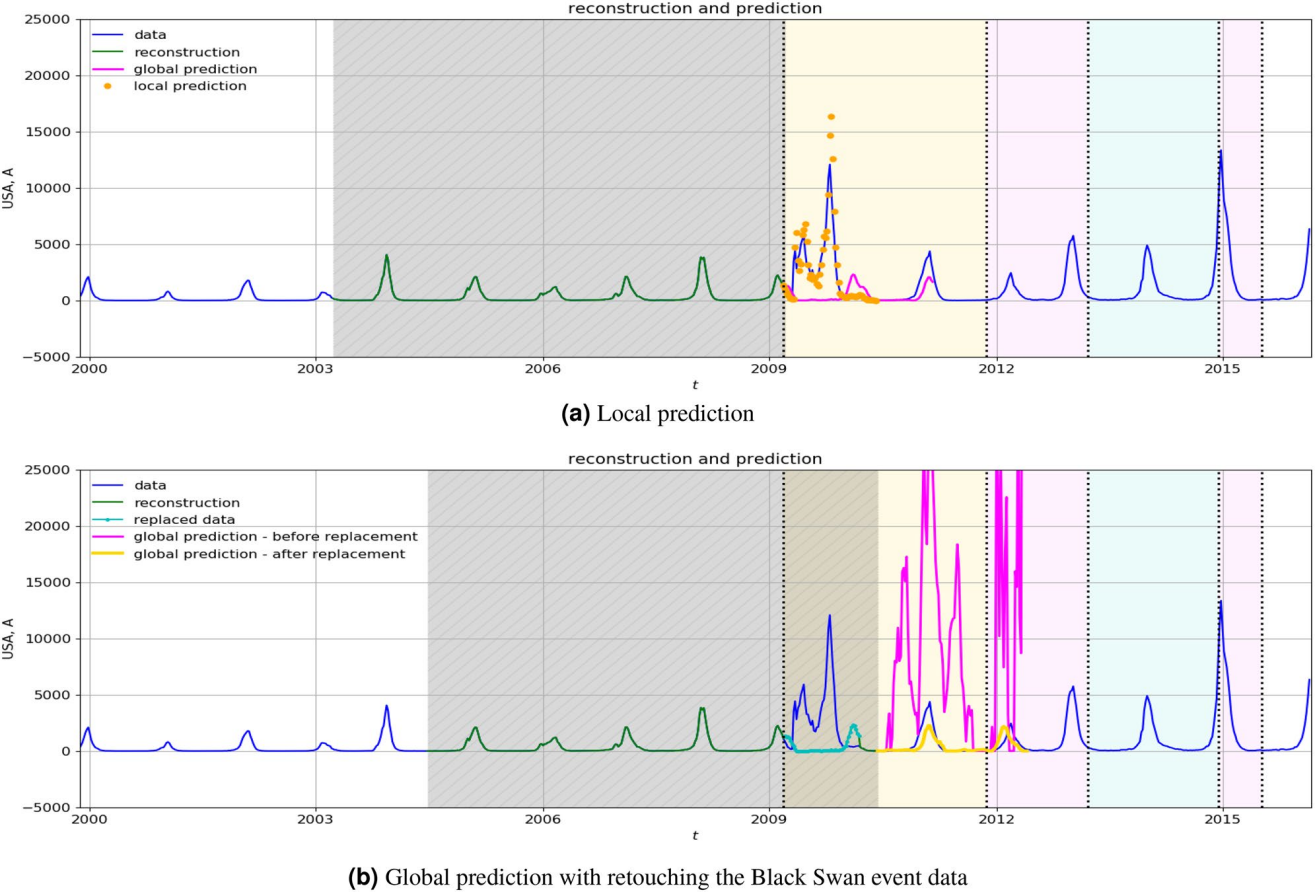
Influenza data (USA). (**a**) The data are collected in the window April 2003–April 2009 (shadowed rectangle) and then the dynamics is predicted for 104 weeks ahead. The local prediction algorithm recovers the prediction capability by forgetting the old data and using narrower learning windows. The local prediction algorithm delivers prediction for one week ahead. (**b**) The active window (shadowed rectangle) is July 2004–July 2010, and the dynamics is predicted for 104 weeks ahead. The global prediction fails due to the Black Swan data in the learning window. (Some predicted values were even negative; those were replaced with zeros.) The global prediction algorithm recovers after the retouching the Black Swan event data, which allows for using big learning window. Compare with positions of the corresponding colored rectangles in Fig. 2.

#### Retouching the Black Swan event data

Next, we introduce an approach that robustifies the global algorithm in the presence of disturbances in the data, including the missing data scenario. We use the data window July 2004–July 2010, which contains a Black Swan event in the period 2009–2010. As shown in Fig. 1b, the learned KMD failed to predict the future following the active training window. This is expected because the perturbation caused by the Black Swan event resulted in the computed Ritz pairs that deviated from the precedent ones (from a learning window before disturbance), and, moreover, with most of them having large residuals. This can be seen as a second type of failure in the global prediction.

The proposed Black Swan event detecting device, built in the prediction algorithm (see Supplementary Information Algorithm S3), checks for this anomalous behaviour of the Ritz values and pinpoints the problematic subinterval. Then, the algorithm replaces the corresponding supplied data with the values obtained as predictions based on the time interval preceding the Black Swan event. Figure 1b shows that such a retouching of the disturbance allows for a reasonable global prediction.

Note that in a realistic situation, global predictions of this kind will trigger response from authorities and therefore prevent its own accuracy and induce loss of confidence, whereas local prediction mechanisms need to be deployed again.

**Figure 2:**
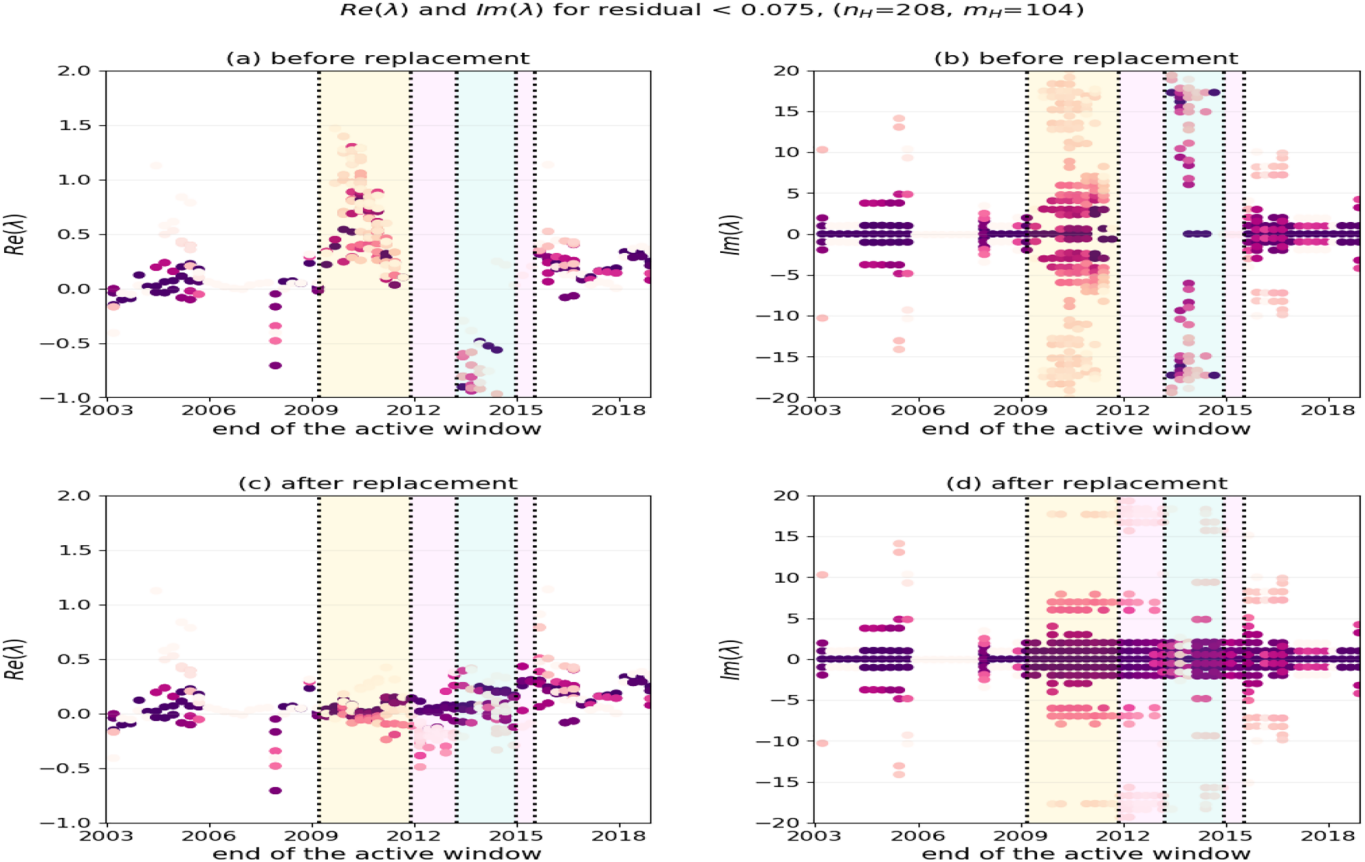
The real and imaginary parts of Ritz values with residuals bellow $\eta _r=0.075$ for sliding active windows. The color intensity of eigenvalues indicates the amplitudes of the corresponding modes. Pink rectangles mark ends of training windows with no acceptable Ritz values. Note how the unstable eigenvalues ($\Re (\lambda )>0$) impact the prediction performance, and how the retouching moves them towards neutral/stable—this is shown in the yellow rectangle in panels (**a**) and (**c**). Also influenced by the disturbance are the eigenvalues in the light blue rectangles in panels (**a**), (**b**); retouching moves the real parts of eigenvalues towards neutral/stable and rearranges them in a lattice-like structure^22^, as shown in panels (**c**), (**d**). Compare with Fig. 1b.

#### Monitoring and restoring the Ritz values

We now discuss the effect of the Black Swan event and its retouching to the computed eigenvalues and eigenvectors. We have observed that, as soon as a disturbance starts entering the training windows, the Ritz values start exhibiting atypical behavior, e.g. moving deeper into the right half plane (i.e. becoming more unstable), and having larger residuals because the training data no longer represent the Krylov sequence of the underlying Koopman operator.

This is illustrated in the panels (a) and (b) in Fig. 2, which show, for the sliding training windows, the real and the imaginary parts of those eigenvalues for which the residuals of the associated eigenvectors are smaller than $\eta _r=0.075$. Note the absence of such eigenvalues in time intervals that contain the disturbance caused by the Black Swan event.

On the other hand, the retouching technique that repairs the distorted training data restores the intrinsic dynamics over the entire training window. The distribution of the relevant eigenvalues becomes more consistent, and the prediction error decreases, see panels (c) and (d) in Fig. 2, and in Supplementary Information Figure S16.

#### Discussion

Our proposed retouching procedure relies on detecting anomalous behavior of the Ritz values; a simple strategy of monitoring the spectral radius of active windows (absolutely largest Ritz value extracted from the data in that window) is outlined in Supplementary Information. Note that this can also be used as a *litmus test* for switching to the local prediction algorithm. In Supplementary Information, we provide further examples, with the influenza data, that confirm the usefulness of the retouching procedure. In general, this procedure can also be adapted to the situation when the algorithm receives a signal that the incoming data is missing or corrupted.

### COVID-19 prediction

The second set of data we consider is that associated with the ongoing COVID-19 pandemic. Because the virus is new, the whole event is, in a sense, a “Black Swan”. However, as we show below, the prediction approach advanced here is capable of adjusting quickly to the new incoming, potentially sparse data and is robust to inaccurate reporting of cases.

At the beginning of the spread of COVID-19, we have witnessed at moments rather chaotic situation in gaining the knowledge on the new virus and the disease. The development of COVID-19 diagnostic tests made tracking and modeling feasible, but with many caveats: the data itself is clearly not ideal, as it depends on the reliability of the tests, testing policies in different countries (triage, number of tests, reporting intervals, reduced testing during the weekends), contact tracing strategies, using surveillance technology, credit card usage and phone contacts tracking, the number of asymptomatic transmissions etc. Many different and unpredictable exogenous factors can distort it. So, for instance the authors of^23^ comment at https://ourworldindata.org/coronavirus-testing that e.g. “The Netherlands, for instance, makes it clear that not all labs were included in national estimates from the start. As new labs get included, their past cumulative total gets added to the day they begin reporting, creating spikes in the time series.” For a prediction algorithm, this creates a Black Swan event that may severely impair prediction skills, see section “Retouching the Black Swan event data”.

This poses challenging problems to the compartmental type models of (SIR, SEIR) which in order to be useful in practice have to be coupled with data assimilation to keep adjusting the key parameters, see e.g.^24^. Our technique of retouching (section “Retouching the Black Swan event data”) can in fact be used to assist data assimilation by detecting Black Swan disturbance and thus to avoid assimilating disturbance as normal.

In the KMD based framework, the changes in the dynamics are automatically assimilated on-the-fly by recomputing the KMD using new (larger or shifted) data snapshot windows. This is different from the compartmental type models of infectious diseases, most notably in the fact that the procedure presented here does not assume any model and, moreover, that it is entirely oblivious to the nature of the underlying process.

### An example: European countries

As a first numerical example, we use the reported cumulative daily cases in European countries. In Supplementary Information section S1.5, we use this data for a detailed worked example that shows all technical details of the method. This is a good test case for the method—using the data from different countries in the same vector observable poses an additional difficulty for a data driven revealing of the dynamics, because the countries independently and in an uncoordinated manner impose different restrictions, thus changing the dynamics on local levels. For instance, at the time of writing these lines, a new and seemingly more infectious strain of the virus circulating in some parts of London and in south of England prompted the UK government to impose full lockdown measures in some parts of the United Kingdom. Many European countries reacted sharply and immediately suspended the air traffic with the UK.

**Figure 3:**
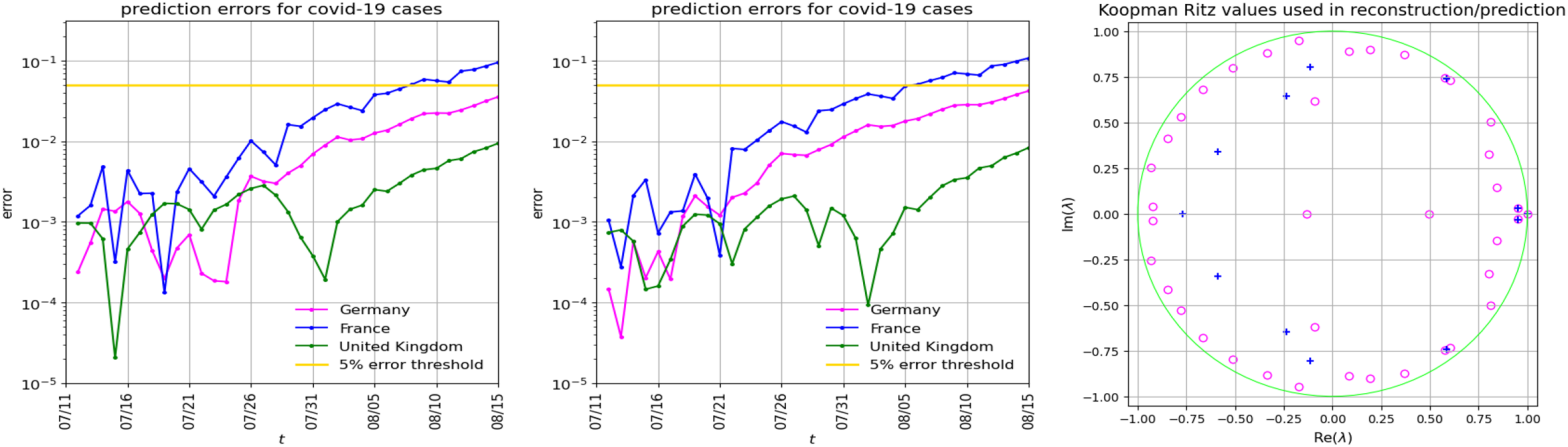
Prediction of COVID-19 cases (35 days ahead, starting July 11) for Germany, France and United Kingdom. Left panel: The Hankel–Takens matrix ${\mathbb {H}}$ is $282 \times 172$, the learning data consists of ${\textbf{h}}_{1:40}$. The KMD uses 39 modes. *Middle panel*: The matrix ${\mathbb {H}}$ is $363 \times 145$, the learning data is ${\textbf{h}}_{1:13}$. The KMD uses 12 modes. Right panel: The Koopman–Ritz values corresponding to the first (magenta circles) and the middle (blue plusses) panel. Note how the three rightmost values nearly match.

**Figure 4:**
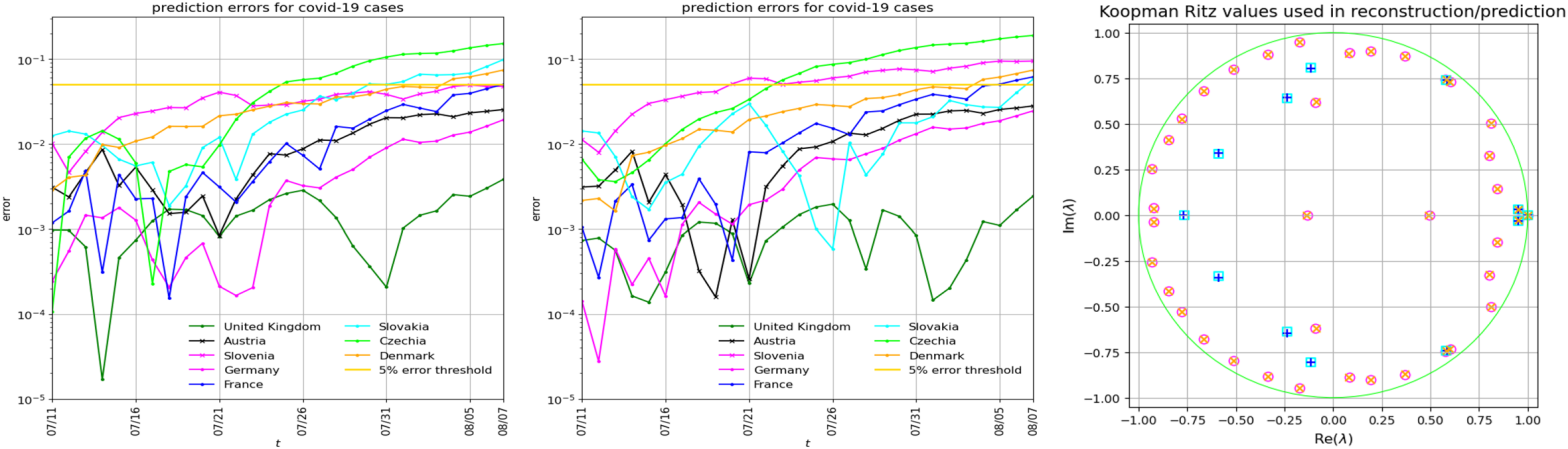
Prediction errors and KMD spectrum of COVID-19 cases (28 days ahead, starting July 11) for Germany, France, United Kingdom, Denmark, Slovenia, Czechia, Slovakia and Austria. Left panel: The Hankel–Takens matrix ${\mathbb {H}}$ is $752 \times 172$, the learning data consists of ${\textbf{h}}_{1:40}$. The KMD uses 39 modes. Middle panel: The matrix ${\mathbb {H}}$ is $968 \times 145$, the learning data is ${\textbf{h}}_{1:13}$. The KMD uses 12 modes. Right panel: The Koopman–Ritz values corresponding to the first two computations in Fig. 3 (magenta circles and blue pluses, respectively) and the the first two panels in this Figure (orange x-es and cyan squares, respectively). Note how the corresponding Koopman–Ritz values nearly match for all cases considered.

In the first numerical experiment, we use two datasets from the time period February 29 to November 19. and consider separately two sets of countries: Germany, France and the UK in the first, and Germany, France, UK, Denmark, Slovenia, Czechia, Slovakia and Austria in the second. The results for a particular prediction interval are given in Figs. 3 and  4. For more examples and discussion how the prediction accuracy depends on the Government Response Stringency Index (GRSI^25,26^) see Supplementary Information section S1.5.

**Figure 5:**
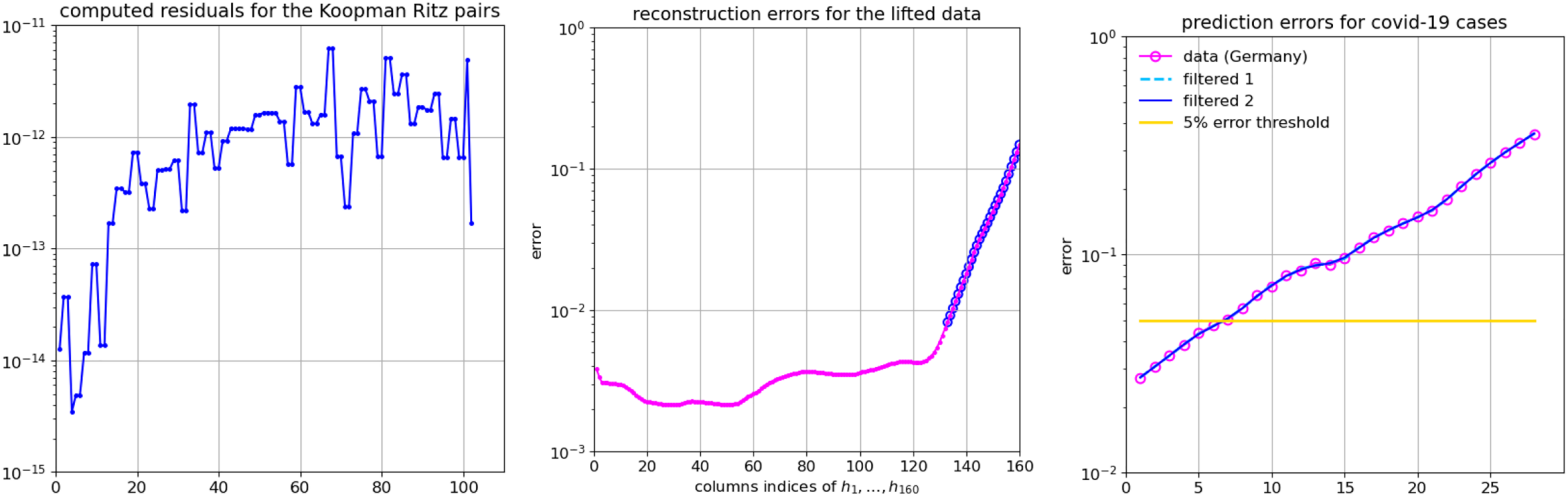
Prediction experiment with data from Germany. Left panel: the computed residuals for the computed 102 Koopman Ritz pairs (extracted from a subspace spanned by 132 snapshots ${\textbf{h}}_{1:132}$). Note that all residuals are small. The corresponding Ritz values are shown in the first panel in Fig. 6. Middle panel: KMD reconstruction error for ${\textbf{h}}_{1:132}$ and the error in the predicted values ${\textbf{h}}_{133:160}$ (encircled with ${\circ }$). The reconstruction is based on the coefficients $(\alpha _j)_{j=1}^r=\mathrm {arg\min }_{\alpha _j}\sum _{k} \Vert {\textbf{h}}_k - \sum _{j=1}^{r} \lambda _j^{k}\alpha _j {\textbf{v}}_j\Vert _2^2$. Right panel: Prediction errors for the period October 11–November 7.

**Figure 6:**
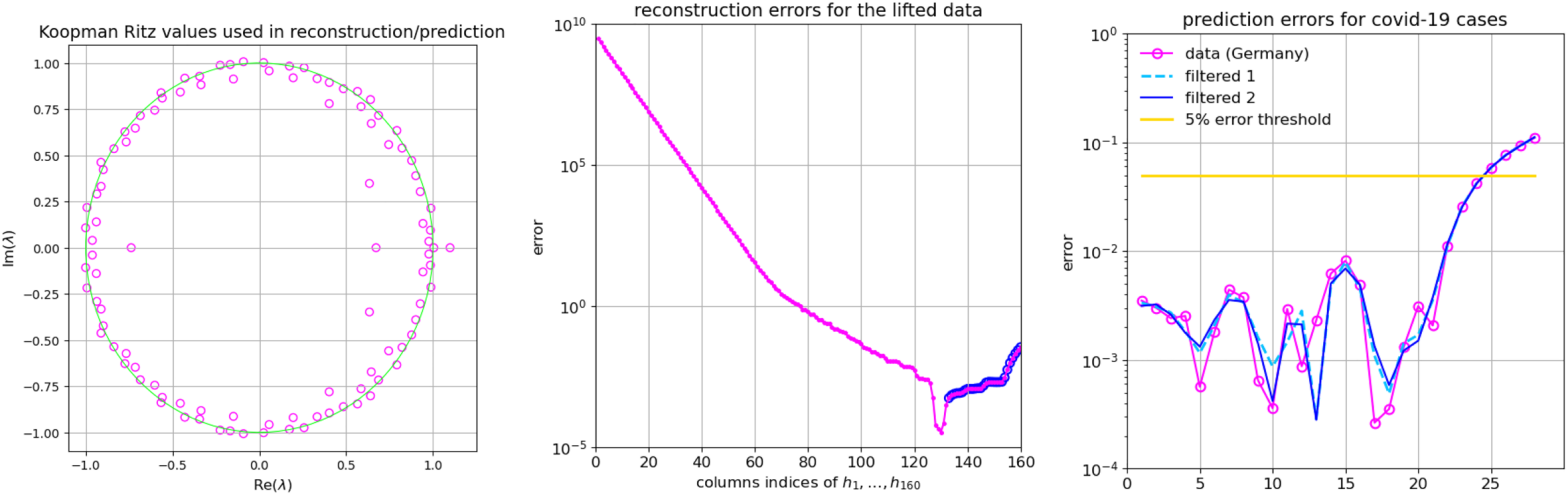
Prediction experiment with **DS3** with data from Germany. Left panel: the computed 102 Koopman Ritz values (extracted from a subspace spanned by 132 snapshots ${\textbf{h}}_{1:132}$). The corresponding residuals are shown in the first panel in Fig. 5. Middle panel: KMD reconstruction error for ${\textbf{h}}_{1:132}$ and the error in the predicted values ${\textbf{h}}_{133:160}$ (encircled with ${\circ }$). The reconstruction is based on the coefficients $(\alpha _j)_{j=1}^r=\mathrm {arg\min }_{\alpha _j}\sum _{k} w_k^2 \Vert {\textbf{h}}_k - \sum _{j=1}^{r} \lambda _j^{k}\alpha _j {\textbf{v}}_j\Vert _2^2$. Right panel: Prediction errors for the period October 11 – November 7. Compare with the third graph in Fig. 5.

In the above examples, the number of the computed modes was equal to the dimension of the subspace of spanned by the training snapshots, so that the KMD of the snapshots themselves was accurate up to the errors of the finite precision arithmetic. In general, that will not be the case, and the computed modes will span only a portion the training subspace, meaning that the KMD of the snapshots might have larger representation error. (Here we refer the reader to Supplementary Information section S1.3, where all technical details are given.) This fact has a negative impact to the extrapolation forward in time and the problem can be mitigated by giving more importance to reconstruction of more recent weights. This is illustrated in Figs. 5 and 6, where the observables are the raw data (reported cases) for Germany, extended by a two additional sequence of filtered (smoothened) values.

The figures illustrate an important point in prediction methodology, that we emphasized in the introduction: a longer dataset and a better data reconstruction ability (i.e. interpolation) does not necessarily lead to better prediction. Namely, weighting more recent data more heavily produces better prediction results. This was already observed in^27^ for the case of traffic dynamics, and the method we present here can be used to optimize the prediction ability.

#### An example: USA and worldwide data

We have deployed the algorithm to assess the global and United States evolution of the COVID-19 pandemic. The evolution of the virus is rapid, and “Black Swans” in the sense of new cases in regions not previously affected appear with high frequency. Despite that, the Koopman Mode Decomposition based algorithm performed well.

**Figure 7:**
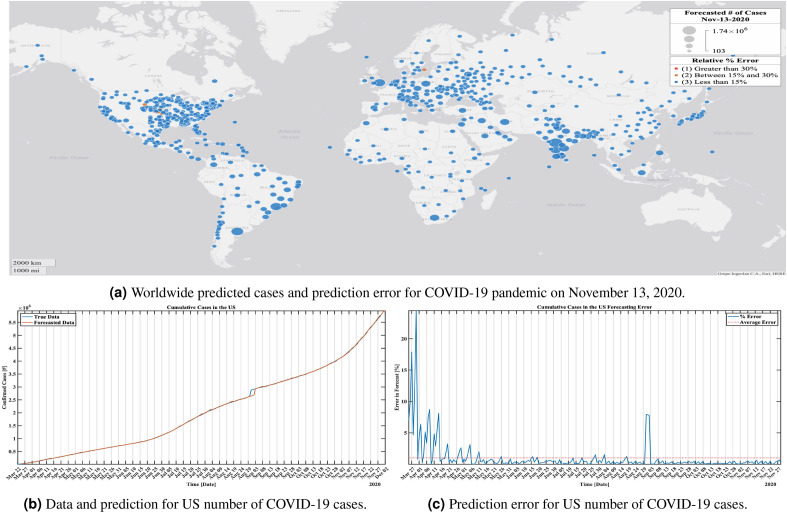
Prediction of confirmed COVID-19 cases utilizing the publicly available COVID-19 data repository provided by Johns Hopkins. The true data ranges between March 22nd, 2020 and November 29th, 2020. We utilize the last three days of data to forecast the following three days of data. (**a**) Predicted conditions and prediction error worldwide on November 13. The widths of the bubbles represent the number of cases in a region; only regions with more that 100 cases are used and the bubbles are colored according to their relative percent error. (**b**) Comparison of true and forecast data for cumulative confirmed cases in the US for April to December 2020. The cumulative forecasts shown here were obtained by summing the forecasts of the individual locations, indicating that the region specific forecasts were sufficiently accurate for tracking the cumulative dynamics of the virus in the US. (**c**) Percent error for the forecasts of the cumulative confirmed cases in the US. On average the percent error is less than 5 percent and although spikes occur, which could be due to changes in testing availability, the algorithm adjusts and the error stabilizes within a short amount of time. Furthermore, Johns Hopkins provided data for around 1787 locations around the United States and we produced forecasts for each of those locations.

In Fig. 7a we show the worldwide forecast number of confirmed cases produced by the algorithm for November 13th, 2020. The forecasts were generated by utilizing the previous three days of data to forecast the next three days of data for regions with higher than 100 cases reported. The bubbles in Fig. 7a are color coded according to their relative percent error. As can be observed, a majority of the forecasts fell below 15% error. The highest relative error for November 13th, 2020 was 19.8% which resulted from an absolute error of 196 cases. The mean relative percent error, produced by averaging across all locations, is 1.8% with a standard deviation of 3.36% for November 13th, 2020. Overall, the number of confirmed cases are predicted accurately and since the forecasts were available between one to three days ahead of time, local authorities could very well utilize our forecasts to focus testing and prevention measures in hot-spot areas that will experience the highest growth.

A video demonstrating the worldwide forecasts for March 25, 2020–November 29, 2020 is provided in the Supplementary Information online (Fig. 7a is a snapshot from that video). Lastly, it is well known that the ability to test people for the virus increased throughout the development of the pandemic and thus resulted in changes in the dynamics of reported cases. Although it is impossible for a data-driven algorithm to account for changes due to external factors, such as increased testing capabilities, it is important that the algorithm be able to adjust and relearn the new dynamics. For this reason, we encourage the reader to reference the video and note that although periods of inaccuracy due to black swan events occur, the algorithm is always able to stabilize and recover. In contrast, since this is at times a rapidly (exponentially) growing set of data, methods like naive persistence forecast do poorly.

In Fig. 7b, c we show the performance of the prediction for the cumulative data for the US in March-April 2020. It is of interest to note that the global curve is obtained as a sum of local predictions shown in Fig. 7a, rather than as a separate algorithm on the global data. Again, the performance of the algorithm on this nonstationary data is good.

## Discussion

In this work, we have presented a new paradigm for prediction in which the central tenet is understanding of the confidence with which the algorithm is capable of predicting the future realizations of a non-stationary stochastic process. Our methodology is based on Koopman operator theory^6^. Operator-theoretic methods have been used for detection of change in complex dynamics in the past, based on both Koopman^18,28^ and Perron–Frobenius operators^29^. Other methods include variational finite element techniques combined with information theoretic measure (Akaike’s information criterion) and maximum entropy principle^30^.

Our approach to the problem of prediction of nonstationary processes has several key ingredients. First, the Koopman operator on the space of the observables is used as a global linearization tool, whose eigenfunctions provide a coordinate system suitable for representation of the observables. Second, in a numerical computation, we lift the available snapshots to a higher dimensional Hankel–Takens structure, which in particular in the case of abundance of data, allows for better numerical (finite dimensional) Rayleigh–Ritz approximation of eigenvalues and eigenvectors of the associated Koopman operator, as well as the KMD. Third, using our recent implementation of the DMD, we select the Koopman modes that have smallest residuals, and thus highest confidence, which is the key for the prediction capabilities of the KMD. In the absence of enough modes with reasonably small residuals, i.e. low confidence, we switch to local prediction, with narrower learning windows and shorter lead time. By monitoring the prediction error, the algorithm may return back to global prediction.

Our methodology is entirely consistent with the typical training/test dataset validation techniques in machine learning. Namely, the globally learned model on the training data is applied to test data for the next time interval. The novelty in our approach is that we constantly check for how well the learned model generalizes, and if it does not generalize well, we restart the learning. One can say that we implemented a feedback loop, within which the machine learning algorithm’s generalizability from training to test dataset is constantly checked, and the system adapts to new conditions. Evidence for effectiveness of this procedure is presented for the COVID-19 prediction example, where we show how the generalization error diminishes over time.

We emphasize that this a general method that is model-free and completely data-driven. It adapts organically to changes in the underlying system. Contrast this in the context of epidemiology where SIR-type models are used. For a changing driving dynamics, the SIR modeling approach would need to be coupled with a data-assimilation approach to offer the same adaptability as our method.

### Supplementary Information

Supplementary Information 1.

Supplementary Information 2.

## Data Availability

The raw COVID-19 data is made publicly available by the Center for Systems Science and Engineering (CSSE) at Johns Hopkins University at https://github.com/CSSEGISandData/COVID-19 . The raw Influenza data is made publicly available by the World Health Organization at https://www.who.int/tools/flunet The raw geomagnetic storm data is made publicly available by the National Aeronautics and Space Administration at https://omniweb.gsfc.nasa.gov/form/omni_min.html.
