# Supplementary material for: A Koopman operator-based prediction algorithm and its application to COVID-19 pandemic and influenza cases
Source: Sci Rep. 2024 Mar 9;14:5788. doi: 10.1038/s41598-024-55798-9 (PMC10924939; doi:10.1038/s41598-024-55798-9)
Supplement: Supplementary file 2 — Supplementary Information 2. [file 41598_2024_55798_MOESM2_ESM.pdf]

Supplementary Information for

A Koopman Operator-Based Prediction Algorithm and its Application to COVID-19 Pandemic

Igor Mezić, Zlatko Drmač, Nelida Črnjarić-Žic, Senka Maćešić, Maria Fonoberova, Ryan Mohr, Allan M. Avila, Iva Manojlović, Aleksandr Andrejčuk

S1 Methods

In this section we provide technical details of the numerical spectral analysis of dynamical systems, which is at the core of the prediction algorithms presented in this work. The tools of trade are the Koopman operator, the KMD and the DMD decompositions. We first present a compact tutorial on the numerical aspects of the Koopman modal analysis of nonlinear dynamical systems, and, then, we provide theoretical underpinnings for the methods presented in the paper.

We do not go into the details of convergence of the Koopman operator approximations utilized in the paper, but we mention the associated work. For example, for on-attractor evolution, the properties of the Generalized Laplace Analysis (GLA) method acting on L^2 functions were studied in[1,2]. The off-attractor case was pursued in[3] in Hardy-type spaces. This study was continued in[4] to construct dynamics-adapted Hilbert spaces. A study of convergence of DMD-type approximations utilized in this paper is provided in[5]. There are two types of results presented in these papers 1) Convergence of the spectral objects over an infinite time interval and 2) rate of convergence to spectral objects. Since we are pursuing a finite-time analysis, the results of type 2) are more relevant. The gist of these results is that the convergence rate is $1/n$ for regular dynamics (limit cycles, limit tori), where n is the number of snapshots, and $1/\sqrt{n}$ for irregular (chaotic) dynamics. This can be improved to even exponential convergence under some conditions[6]. While the proofs indicated here are for GLA, and depend on convergence time averages over trajectories, they can be extended for DMD methods since in general they can be related to time averages over trajectories[7].

Since the prediction methods in the paper are tightly connected to detection of “Black Swan" events, and these are often defined in imprecise terms, we provide a mathematical definition that we utilize in this work for the orientation of the reader:

Definition S1.1 *Let $f : M \to \mathbb{C}$, $f \in H$ and $K : H \to H$ an operator from the Hilbert space H to itself. Consider the dynamics given by $f' = Kf$. Let μ be an ergodic invariant measure for K, i.e.*

$$\int_M f d\mu = \lim_{n\to\infty} \frac{1}{n}\sum_{j=0}^{n-1} K^j f(x), \tag{S1}$$

for almost all $x \in M$. Assume the support S of μ is such that $S \neq M$. A Black Swan event for an observable f is $g \notin f(S)$. The magnitude of the Black Swan event for observation g is $d(g, f(S))$ where d is a metric, and $f(S)$ is the range of f on S.

The set M does not necessarily need to be the state space. In the examples shown in the paper, the observables are spectral (e.g. the spectral radius), $M = \mathbb{C}$ and the operator K is the operator acting on the spectral objects induced by the Koopman evolution.

For a non-degenerate stochastic process with an ergodic measure μ it can be unlikely that the support of μ is different from M. In that case, the "BlackSwannes" of a value can be defined as $d(g, \nu)$, where d is e.g. the Wasserstein distance of a delta distribution at f and $\nu(E) = \mu(f^{-1}(E))$ is the pushforward measure under f[8]. Note that this particular d can be used in the deterministic case described previously.

S1.1 Organization of the section

This section is organized as follows. First, in §S1.2 we review the continuous and the discrete autonomous dynamical systems, and the definition of the Koopman operator $\mathscr{U}$ on the space of observables. The review material is based on the papers on the theory and applications of the Koopman operator[9],[10],[11], and our recent work[12],[13],[14]. The spatio-temporal representation of the evolution of the observables using the eigenvalues and eigenvectors of the Koopman operator (Koopman mode decomposition, KMD) is discussed in §S1.2.1. The next key ingredient, numerical computation of approximate eigenvalues and eigenvectors, is reviewed in §S1.3. In particular, the details of the matrix representation of a compression of $\mathscr{U}$ to the subspace of observables are worked out in §S1.3.2; numerical realization of the Koopman mode decomposition is presented in detail in §S1.3.3 and §S1.3.4. In §S1.3.5 we provide the key elements of the Schmid's DMD algorithm, and in §S1.3.6 its recent enhancement that allows selection of Ritz pairs that can be used for a KMD suitable for prediction. In §S1.3.7 we review the least squares methods for spatio-temporal representation of the snapshots using the selected Koopman modes. And finally, after having prepared all necessary ingredients, in §S1.4 we present the global prediction algorithm. The setup of the prediction framework is given in §S1.4.1 which introduces basic notation and §S1.4.2, where we lift the data snapshots in a Hankel structure that will

be used in the algorithms. In §S1.4.3, we discuss the limitations of the numerical realization of the KMD based prediction scheme introduced in §S1.2.1. A worked example that illustrates technical details is provided in §S1.5, where we apply the prediction scheme to the spread of the coronavirus disease in European countries. In §S1.7, we discuss the problem of Black Swan events[15] in the training data, and we propose a novel technique, based on the method reviewed in §S1.3.6, for detecting and retouching Black Swan type disturbances of the data. This makes the global prediction scheme resilient to sudden and unpredictable disturbances that have become part of the learning data window. In §S1.7.2, we present a more flexible local prediction scheme that dynamically resizes the learning data windows and the forecasting lead time in an event of sudden changes and large prediction errors.

S1.2 Setting the scene: Koopman operator

Consider an autonomous system of differential equations

$$\dot{\mathbf{x}}(t) = \mathbf{F}(\mathbf{x}(t)) \equiv \begin{pmatrix} \mathbf{F}_1(\mathbf{x}(t)) \\ \vdots \\ \mathbf{F}_N(\mathbf{x}(t)) \end{pmatrix}, \tag{S2}$$

with state space $\mathscr{X}$ and vector-valued nonlinear function $\mathbf{F}$. Here $\mathscr{X}$ is a compact smooth N-dimensional manifold, endowed with a Borel sigma algebra $\mathscr{B}$, and for simplicity identified with a subset of $\mathbb{R}^N$, with $\mathbf{F} : \mathscr{X} \longrightarrow \mathbb{R}^N$. The associated flow map $\varphi^t : \mathscr{X} \longrightarrow \mathscr{X}$ advances an initial state $\mathbf{x}(t_0)$ forward in time by a time unit t,

$$\mathbf{x}(t_0+t) = \varphi^t(\mathbf{x}(t_0)) = \mathbf{x}(t_0) + \int_{t_0}^{t_0+t} \mathbf{F}(\mathbf{x}(\tau))d\tau. \tag{S3}$$

Note that $\varphi^{t+s} = \varphi^t \circ \varphi^s$, where $\circ$ denotes the composition of mappings. The (internal) state is often inaccessible; instead an observable (output) is given as a function $f : \mathscr{X} \longrightarrow \mathbb{C}$ of the state, where the class (function space) $\mathscr{F} \ni f$ of observables is appropriately chosen and endowed with a Banach or Hilbert space structure. For more detailed introduction we refer to[16], in particular Chapters II and V, and[17]. For instance, we can take $\mathscr{F} = L^p(\mathscr{X}, \mu)$, $1 \le p \le \infty$, with an appropriate measure μ and e.g. for $p = 2$ with the corresponding Hilbert space structure.

The Koopman operator semigroup $(\mathscr{U}_{\varphi^t})_{t\ge 0}$ is defined by

$$\mathscr{U}_{\varphi^t} f = f \circ \varphi^t, \;\; f \in \mathscr{F}. \tag{S4}$$

Here we assume that φ^t preserves sets of measure zero (if $\mu(A) = 0$, then $\mu((\varphi^t)^{-1})(A) = 0$) and that $\mathscr{U}_{\varphi^t}$ is defined on the equivalency classes (modulo μ). It can be considered as a linearization tool for (S2): $\mathscr{U}_{\varphi^t}$ is a linear operator that allows studying (S2) by examining its action on the infinitely dimensional space $\mathscr{F}$ of observables. If φ^t is measure-preserving ($(\forall A \in \mathscr{B})$ $(\mu((\varphi^t)^{-1}(A)) = \mu(A))$ then $\mathscr{U}_{\varphi^t}$ is an isometry. For an introduction to the theory of the Koopman operator on the Banach lattice L^p see [17, Chapter 7].

An analogous approach is applicable to a discrete dynamical system

$$\mathbf{z}_{i+1} = \mathbf{T}(\mathbf{z}_i), \tag{S5}$$

where $\mathbf{T} : \mathscr{X} \longrightarrow \mathscr{X}$ is a measurable nonlinear map on a state space $\mathscr{X}$ and $i \in \mathbb{Z}$. The Koopman operator $\mathscr{U} \equiv \mathscr{U}_{\mathbf{T}}$ for the discrete system is defined analogously by

$$\mathscr{U} f = f \circ \mathbf{T}, \;\; f \in \mathscr{F}. \tag{S6}$$

Discrete dynamical systems naturally describe evolution of discrete events, e.g. stock market data, reported cases of influenza illnesses, or lynx population in Europe, but they are also at the core of numerical analysis of the continuous systems. More precisely, if we run a numerical simulation of (S2) in a time interval $[t_0, t_*]$, the numerical solution is obtained on a discrete equidistant grid with fixed time lag Δt:

$$t_0,\, t_1 = t_0 + \Delta t,\, \ldots,\, t_{i-1} = t_{i-2} + \Delta t,\, t_i = t_{i-1} + \Delta t,\, \ldots \tag{S7}$$

In this case, a black-box software toolbox acts as a discrete dynamical system $\mathbf{z}_i = \mathbf{T}(\mathbf{z}_{i-1})$ that produces the discrete sequence of $\mathbf{z}_i \approx \mathbf{x}(t_i)$; this is sampling with noise. For $t_i = t_0 + i\Delta t$ we have (using (S3), (S4) and the group property)

$$f(\mathbf{x}(t_0 + i\Delta t)) = (f \circ \varphi^{i\Delta t})(\mathbf{x}(t_0)) = (\mathscr{U}_{\varphi^{i\Delta t}} f)(\mathbf{x}(t_0)) = (\mathscr{U}^i_{\varphi^{\Delta t}} f)(\mathbf{x}(t_0)), \;\; \mathscr{U}^i_{\varphi^{\Delta t}} = \mathscr{U}_{\varphi^{\Delta t}} \circ \ldots \circ \mathscr{U}_{\varphi^{\Delta t}}. \tag{S8}$$

On the other hand, using (S6),

$$f(\mathbf{z}_i) = f(\mathbf{T}(\mathbf{z}_{i-1})) = \ldots = f(\mathbf{T}^i(\mathbf{z}_0)) = (\mathscr{U}^i f)(\mathbf{z}_0), \tag{S9}$$

where $\mathbf{T}^2 = \mathbf{T} \circ \mathbf{T}$, $\mathbf{T}^i = \mathbf{T} \circ \mathbf{T}^{i-1}$. Hence, in a software simulation of (S2) with the initial condition $\mathbf{z}_0 = \mathbf{x}(t_0)$, we have an approximation

$$(\mathscr{U}^i f)(\mathbf{z}_0) \approx (\mathscr{U}^i_{\varphi^{\Delta t}} f)(\mathbf{z}_0), \;\; f \in \mathscr{F}, \, \mathbf{z}_0 \in \mathscr{X}, \;\; i = 0, 1, 2, \ldots \tag{S10}$$

with the fidelity that depends on the numerical scheme deployed in the software, and which is studied in the shadowing theory, see e.g.[18],[19].

This can be obviously extended to vector valued observables: for $\mathbf{g} = (g_1, \ldots, g_d) : \mathscr{X} \longrightarrow \mathbb{C}^d$ we define

$$\mathscr{U}_d \mathbf{g} = \begin{pmatrix} g_1 \circ \mathbf{T} \\ \vdots \\ g_d \circ \mathbf{T} \end{pmatrix} = \begin{pmatrix} \mathscr{U} g_1 \\ \vdots \\ \mathscr{U} g_d \end{pmatrix}. \tag{S11}$$

The observables can be both purely physical quantities (e.g. temperature, pressure, energy) and mathematical constructs using suitable classes of functions (e.g. (multivariate) Hermite polynomials, radial basis functions). In particular, if we set $d = N$, $g_i(\mathbf{z}) = e_i^T \mathbf{z}$, where $\mathbf{z} \in \mathbb{C}^N$, $e_i = (\delta_{ji})_{j=1}^N$, $i = 1, \ldots, N$, then $\mathbf{g}(\mathbf{z}) = \mathbf{z}$ is called full state observable and $(\mathscr{U}_d \mathbf{g})(\mathbf{z}_i) = \mathbf{z}_{i+1}$.

S1.2.1 Spectral decomposition and representation of observables

Spectral decomposition of $\mathscr{U}$ is the pillar of both theoretical and practical analysis of dynamical systems in the framework of the Koopman linearizations (S4), (S6). We consider (S6) with $\mathscr{F} = L^2(\mathscr{X}, \mu)$, where $\mathscr{X}$ is compact and big enough to contain the states.

An eigenpair (λ_j, ψ_j) of the eigenvalue $\lambda_j \in \mathbb{C}$ and nonzero function $\psi_j \in \mathscr{F}$ (eigenvector, eigenfunction) satisfies

$$\mathscr{U} \psi_j = \lambda_j \psi_j.$$

The key of the spectral analysis of the dynamical system is a representation of an observable as a linear combination of the eigenfunctions of $\mathscr{U}$. Since the Koopman operator can have continuous spectrum[20,21], this might not be always possible. However, an observable that belongs to a subspace spanned by eigenfunctions can be written as

$$\mathbf{h}(\mathbf{z}) = \begin{pmatrix} h_1(\mathbf{z}) \\ \vdots \\ h_\ell(\mathbf{z}) \end{pmatrix} \approx \sum_{j=1}^{\infty} \psi_j(\mathbf{z}) \mathbf{v}_j, \;\text{ where }\; h_i(\mathbf{z}) \approx \sum_{j=1}^{\infty} \psi_j(\mathbf{z}) (\mathbf{v}_j)_i, \;\; \mathbf{v}_j = \begin{pmatrix} (\mathbf{v}_j)_1 \\ \vdots \\ (\mathbf{v}_j)_\ell \end{pmatrix}. \tag{S12}$$

Then we can envisage the values of the observable $\mathbf{h}$ at the *future* states $\mathbf{T}(\mathbf{z})$, $\mathbf{T}^2(\mathbf{z}), \ldots$ by

$$\mathbf{h}(\mathbf{T}(\mathbf{z})) = \begin{pmatrix} h_1(\mathbf{T}(\mathbf{z})) \\ \vdots \\ h_\ell(\mathbf{T}(\mathbf{z})) \end{pmatrix} = \begin{pmatrix} (\mathscr{U} h_1)(\mathbf{z}) \\ \vdots \\ (\mathscr{U} h_\ell)(\mathbf{z}) \end{pmatrix} = (\mathscr{U}_d \mathbf{h})(\mathbf{z}) \approx \sum_{j=1}^{\infty} \lambda_j \psi_j(\mathbf{z}) \mathbf{v}_j, \ldots \;\; \mathbf{h}(\mathbf{T}^k(\mathbf{z})) \approx \sum_{j=1}^{\infty} \lambda_j^k \psi_j(\mathbf{z}) \mathbf{v}_j, \ldots \tag{S13}$$

For more details see e.g.[22],[23],[24]. If the dynamics is evolving on an attractor, then all eigenvalues are on the unit circle; however we are also interested in an off-attractor analysis. The mapping φ^t is thus not assumed measure preserving, and thus $\mathscr{U}$ is not necessarily unitary. Detailed analysis of the spectrum in this general case and the function spaces associated with it can be found in[22].

S1.3 Numerical computation

For a practical application of the Koopman operator, we need numerical methods to compute its approximate eigenvalues and eigenvectors, and the modes $\mathbf{v}_j$ in (S12). We consider the discrete case (S5), (S6), (S11); for numerical computations with a continuous system we invoke the discretization (S8), (S9), (S10). For more details on the Extended DMD and implementation using the kernel trick see[11].

S1.3.1 The data

In a typical data driven setting, we will have a sequence of snapshots, where we use the notion of snapshot as a numerical value of a scalar or vector valued observable at a specific instance in time. We do not assume explicit knowledge of the mappings $\mathbf{F}$ (S2) or $\mathbf{T}$ (S5). For example, the snapshots may be obtained from high speed camera recording of a combustion process in a turbine[25], or. e.g. as the wind tunnel measurements. A less expensive and less restrictive is a numerical simulation of (S2)

represented by (S8), (S9), (S10), where we can feed an initial $\mathbf{z}_0$ to a software tool (representing $\mathbf{T}$, or its linearization through a numerical scheme encoded in the software toolbox) to obtain the sequence

$$\mathbf{f}(\mathbf{z}_0) = (\mathscr{U}_d^0\mathbf{f})(\mathbf{z}_0),\ \mathbf{f}(\mathbf{z}_1) = (\mathscr{U}_d\mathbf{f})(\mathbf{z}_0),\ \mathbf{f}(\mathbf{z}_2) = (\mathscr{U}_d^2\mathbf{f})(\mathbf{z}_0), \ldots, \mathbf{f}(\mathbf{z}_{M+1}) = (\mathscr{U}_d^{M+1}\mathbf{f})(\mathbf{z}_0), \tag{S14}$$

where $\mathbf{f} = (f_1, \ldots, f_d)^T$ is a vector valued ($d > 1$) observable with the action of $\mathscr{U}_d$ defined by (S11). The time resolution Δt can be set to obtain the desirable numerical accuracy. This can then be repeated for many initial values $\mathbf{z}_0$; if the new initial value is defined as $\widetilde{\mathbf{z}}_0 = p(\mathbf{z}_0)$, then the new simulation data can be incorporated by adding the new observables $f_i \circ p$ as new components of $\mathbf{f}$. In a CFD application, $\mathbf{f}$ may be the full state observable, and the entries in the state vectors $\mathbf{z}_i$ are then e.g. the values of the pressure and of the components of the velocity at a discrete spatial grid in the physical domain.

Hence, independent of the underlying process, the numerical data values are of the form of a matrix $\mathbf{S}$ with columns, respectively, $\mathbf{f}(\mathbf{z}_0)$, $\mathbf{f}(\mathbf{z}_{k+1}) = (\mathscr{U}_d\mathbf{f})(\mathbf{z}_k)$, $k = 0, \ldots, M$:

$$\mathbf{S} = \begin{pmatrix} \mathbf{f}(\mathbf{z}_0) & \mathbf{f}(\mathbf{z}_1) & \mathbf{f}(\mathbf{z}_2) & \ldots & \mathbf{f}(\mathbf{z}_M) & \mathbf{f}(\mathbf{z}_{M+1}) \end{pmatrix} = \begin{pmatrix} f_1(\mathbf{z}_0) & f_1(\mathbf{z}_1) & f_1(\mathbf{z}_2) & \ldots & f_1(\mathbf{z}_M) & f_1(\mathbf{z}_{M+1}) \\ f_2(\mathbf{z}_0) & f_2(\mathbf{z}_1) & f_2(\mathbf{z}_2) & \ldots & f_2(\mathbf{z}_M) & f_2(\mathbf{z}_{M+1}) \\ \vdots & \vdots & \vdots & \ldots & \vdots & \vdots \\ f_d(\mathbf{z}_0) & f_d(\mathbf{z}_1) & f_d(\mathbf{z}_2) & \ldots & f_d(\mathbf{z}_M) & f_d(\mathbf{z}_{M+1}) \end{pmatrix} \in \mathbb{C}^{d\times(M+2)},\ \ \mathbf{z}_{k+1} = \mathbf{T}(\mathbf{z}_k),\ \ k = 0, \ldots, M. \tag{S15}$$

Although the snapshots are generated by the nonlinear system (S5), (S9), the recursion (Krylov sequence) (S14), driven by the linear operator $\mathscr{U}_d$ and numerically evaluated along a trajectory initialized at $\mathbf{z}_0$, motivates to seek out a linear operator (matrix) $\mathbb{A} \in \mathbb{C}^{d\times d}$ whose action on the available snapshots is given by

$$\mathbb{A}\mathbf{f}(\mathbf{z}_k) = (\mathscr{U}_d\mathbf{f})(\mathbf{z}_k) = \begin{pmatrix} (\mathscr{U} f_1)(\mathbf{z}_k) \\ \vdots \\ (\mathscr{U} f_d)(\mathbf{z}_k) \end{pmatrix} = \mathbf{f}(\mathbf{T}(\mathbf{z}_k)),\ \ k = 0, \ldots, M. \tag{S16}$$

Thus, if we set $\mathbf{X} = \mathbf{S}(1:d, 1:M+1)$, $\mathbf{Y} = \mathbf{S}(1:d, 2:M+2)$, then such an $\mathbb{A}$ would satisfy $\mathbf{Y} = \mathbb{A}\mathbf{X}$, and this could be extended linearly to the span of the columns of $\mathbf{X}$ by $\mathbb{A}(\mathbf{X}v) = \mathbf{Y}v$, $v \in \mathbb{C}^{M+1}$. The action of $\mathbb{A}$ outside the range of $\mathbf{X}$ is not specified by the available data.

In general, $\mathbf{X}$ and $\mathbf{Y}$ are not necessarily extracted from a single trajectory (S14, S15). The data may consist of several short bursts with different initial conditions, arranged as a sequence of column vector pairs of snapshots $(\mathbf{x}_k, \mathbf{y}_k)$, where $\mathbf{x}_k = \mathbf{f}(\mathbf{z}_k)$, $\mathbf{y}_k = \mathbf{f}(\mathbf{T}(\mathbf{z}_k))$ column-wise so that a kth column in $\mathbf{Y}$ corresponds to the value of the observable in the kth column of $\mathbf{X}$ through the action of $\mathscr{U}_d$, as in (S16); see[26]. Depending on the parameters d and M, the matrices $\mathbf{X}$, $\mathbf{Y}$ can be square, tall (more rows than columns), or wide (more columns than rows). Then, analogously to (S16), we can search for a linear transformation $\mathbb{A}$ such that $\mathbf{Y} = \mathbb{A}\mathbf{X}$. Such an $\mathbb{A}$ may not exist.

However, we can always define a particular matrix $\mathbb{A}$ which minimizes $\|\mathbf{Y} - \mathbb{A}\mathbf{X}\|_F$. Clearly, if $\mathbf{X}^T$ has a nontrivial null-space, $\mathbb{A}$ is not unique; we can choose B so that $B\mathbf{X} = \mathbf{0}$ and thus $(\mathbb{A} + B)\mathbf{X} = \mathbb{A}\mathbf{X}$. One can impose an additional condition of minimality of $\|\mathbb{A}\|_F$, which yields the well known solution $\mathbb{A} = \mathbf{Y}\mathbf{X}^\dagger$, expressed using the Moore-Penrose pseudoinverse $\mathbf{X}^\dagger$ of $\mathbf{X}$. This additional constraint, although useful to enforce uniqueness and boundness, has (to the best of our knowledge) no other useful interpretation in this framework. If we are interested in approximating some eigenvalues and eigenvectors, then with the restricted information on $\mathbb{A}$ (only its action on the range of $\mathbf{X}$ is meaningfully defined), we will use the Rayleigh quotient $\mathbf{X}^\dagger\mathbb{A}\mathbf{X} = \mathbf{X}^\dagger(\mathbb{A} + B)\mathbf{X} = \mathbf{X}^\dagger\mathbf{Y}$, so this non-uniqueness of $\mathbb{A}$ is immaterial. If $\mathbf{X}$ is of full row rank, then the optimal $\mathbb{A}$ is unique. If $\mathbf{X}$ is of full column rank, then $\mathbb{A} = \mathbf{Y}\mathbf{X}^\dagger$ satisfies $\mathbf{Y} = \mathbb{A}\mathbf{X}$ exactly. Throughout this paper we assume that $\mathbf{X}$ is of full (row or column) rank. However, even when full rank, $\mathbf{X}$ may be severely ill-conditioned so that its numerical rank is lower, which poses notrivial numerical challenges; these issues are addressed in our recent work[27].

S1.3.2 A dual representation and the compression of $\mathscr{U}$

The sequence of vector valued observables (S15) naturally describes the (discrete) time dynamics, with the column index representing the timestamp, and row indices representing the scalar observables (e.g. the pressure and the components of the velocity at particular spacial coordinates) that build the vector observable. For instance, in a CFD application, the multi-indexed (2D, 3D) spatial positions are mapped (vectorized) into a column vector in the usual way, and $\mathbf{S}$ is generated by the software, column by column.

In a dual interpretation (reading) of the data, we can think of each row in (S15) as a set of values of an observable, sampled

over the spatial domain. In other words, we transpose the matrix $\mathbf{S}$ in (S15), partition $\widehat{\mathbf{S}} = \mathbf{S}^T$ as

$$\widehat{\mathbf{S}}(1:M+1,1:d) = \begin{pmatrix} f_1(\mathbf{z}_0) & f_2(\mathbf{z}_0) & f_3(\mathbf{z}_0) & \dots & f_d(\mathbf{z}_0) \\ f_1(\mathbf{z}_1) & f_2(\mathbf{z}_1) & f_3(\mathbf{z}_1) & \dots & f_d(\mathbf{z}_1) \\ \vdots & \vdots & \vdots & \dots & \vdots \\ f_1(\mathbf{z}_M) & f_2(\mathbf{z}_M) & f_3(\mathbf{z}_M) & \dots & f_d(\mathbf{z}_M) \end{pmatrix} = \mathbf{X}^T, \tag{S17}$$

$$\widehat{\mathbf{S}}(2:M+2,1:d) = \begin{pmatrix} f_1(\mathbf{T}(\mathbf{z}_0)) & f_2(\mathbf{T}(\mathbf{z}_0)) & f_3(\mathbf{T}(\mathbf{z}_0)) & \dots & f_d(\mathbf{T}(\mathbf{z}_0)) \\ f_1(\mathbf{T}(\mathbf{z}_1)) & f_2(\mathbf{T}(\mathbf{z}_1)) & f_3(\mathbf{T}(\mathbf{z}_1)) & \dots & f_d(\mathbf{T}(\mathbf{z}_1)) \\ \vdots & \vdots & \vdots & \dots & \vdots \\ f_1(\mathbf{T}(\mathbf{z}_M)) & f_2(\mathbf{T}(\mathbf{z}_M)) & f_3(\mathbf{T}(\mathbf{z}_M)) & \dots & f_d(\mathbf{T}(\mathbf{z}_M)) \end{pmatrix} = \mathbf{Y}^T, \tag{S18}$$

and consider the action of $\mathscr{U}$ on the space $\mathscr{F}_{\mathscr{D}}$ spanned by the dictionary of scalar functions $\mathscr{D} = \{f_1,\dots,f_d\}$. That is, we seek a matrix representation $\mathbb{U}$ of the compression $\Psi_{\mathscr{F}_{\mathscr{D}}}\mathscr{U}_{|\mathscr{F}_{\mathscr{D}}} : \mathscr{F}_{\mathscr{D}} \longrightarrow \mathscr{F}_{\mathscr{D}}$, where $\Psi_{\mathscr{F}_{\mathscr{D}}}$ is a suitable projection with the range $\mathscr{F}_{\mathscr{D}}$. This is the standard construction: we need a representation of $\mathscr{U} f_i$ of the form

$$(\mathscr{U} f_i)(z) = f_i(\mathbf{T}(z)) = \sum_{j=1}^{d} \mathbf{u}_{ji} f_j(z) + \rho_i(z), \;\; i = 1,\dots,d, \;\; z \in \mathscr{X}. \tag{S19}$$

With the data at hand, the projection is feasible only in the discrete (algebraic) sense: we can define the matrix $\mathbb{U} = (\mathbf{u}_{ji}) \in \mathbb{C}^{d\times d}$ column-wise by minimizing the residual $\rho_i(z)$ in (S19) over the states $z = \mathbf{z}_k$, using the values

$$(\mathscr{U} f_i)(\mathbf{z}_k) = f_i(\mathbf{T}(\mathbf{z}_k)), \;\; i = 1,\dots,d; \;\; k = 0,\dots,M. \tag{S20}$$

To that end, write the least squares residual

$$\frac{1}{M+1}\sum_{k=0}^{M} |\rho_i(\mathbf{z}_k)|^2 = \frac{1}{M+1}\sum_{k=0}^{M} |\sum_{j=1}^{d} \mathbf{u}_{ji} f_j(\mathbf{z}_k) - f_i(\mathbf{T}(\mathbf{z}_k))|^2, \tag{S21}$$

which is the L^2 residual with respect to the empirical measure defined as the sum of the Dirac measures concentrated at the $\mathbf{z}_k$'s, $\delta_{M+1} = (1/(M+1))\sum_{k=0}^{M} \delta_{\mathbf{z}_k}$. Hence, the columns of the matrix representation are defined as the solutions of the least squares problems

$$\int \left| \sum_{j=1}^{d} \mathbf{u}_{ji} f_j - f_i \circ \mathbf{T} \right|^2 d\delta_{M+1} = \frac{1}{M+1} \left\| \left[\begin{pmatrix} f_1(\mathbf{z}_0) & f_2(\mathbf{z}_0) & \dots & f_d(\mathbf{z}_0) \\ \vdots & \vdots & \dots & \vdots \\ f_1(\mathbf{z}_M) & f_2(\mathbf{z}_M) & \dots & f_d(\mathbf{z}_M) \end{pmatrix} \begin{pmatrix} \mathbf{u}_{1i} \\ \vdots \\ \mathbf{u}_{di} \end{pmatrix} - \begin{pmatrix} f_i(\mathbf{T}(\mathbf{z}_0)) \\ \vdots \\ f_i(\mathbf{T}(\mathbf{z}_M)) \end{pmatrix} \right] \right\|_2^2 \longrightarrow \min_{\mathbf{u}_{1i},\dots,\mathbf{u}_{di}}, \tag{S22}$$

for $i = 1,\dots,d$. The solutions of the above algebraic least squares problems for all $i = 1,\dots,d$ are compactly written as the matrix $\mathbb{U} \in \mathbb{C}^{d\times d}$ that minimizes $\|\mathbf{X}^T\mathbb{U} - \mathbf{Y}^T\|_F$, i.e.

$$\mathbb{U} = (\mathbf{X}^T)^\dagger \mathbf{Y}^T \equiv (\mathbf{Y}\mathbf{X}^\dagger)^T = \mathbb{A}^T, \tag{S23}$$

and the action of $\mathscr{U}$ can be represented, using (S19), as

$$\mathscr{U}\begin{pmatrix} f_1(z) & \dots & f_d(z) \end{pmatrix} = \begin{pmatrix} f_1(z) & \dots & f_d(z) \end{pmatrix}\mathbb{U} + \begin{pmatrix} \rho_1(z) & \dots & \rho_d(z) \end{pmatrix}. \tag{S24}$$

Similarly as with the computation of $\mathbb{A}$ in §S1.3.1, $\mathbb{U}$ is uniquely determined only if $\mathbf{X}^T$ is of full column rank. Otherwise, we must proceed carefully when using the spectral data of $\mathbb{U}$ to infer approximate eigenvalues of $\mathscr{U}$. In particular, if $d > M+1$, $\mathbf{X}^T$ has a nontrivial null-space, and if $\widetilde{\mathbb{U}}$ is another least squares solution, then $\mathbf{X}^T(\widetilde{\mathbb{U}} - \mathbb{U}) = \mathbf{0}$. On the other hand, along the linear manifold $\mathbb{U} + \mathrm{Ker}(\mathbf{X}^T) = \{\mathbb{U} + B \; : \; \mathbf{X}^T B = \mathbf{0}\}$, the Rayleigh quotient (matrix representation of the compression of $\mathbb{U}$ onto the range of $\mathbf{X}$) $\mathbf{X}^\dagger(\mathbb{U}+B)\mathbf{X} = \mathbf{X}^\dagger\mathbb{U}\mathbf{X} \in \mathbb{C}^{(M+1)\times(M+1)}$ remains uniquely determined. (Note that in the case of complex data we work with the adjoints $\mathbf{X}^*$ and $\mathbf{Y}^*$, instead of $\mathbf{X}^T$ (S17) and $\mathbf{Y}^T$ (S18), to obtain $\mathbb{U} = \mathbb{A}^* = \overline{A}^T$, which is then the matrix representation in the basis of complex conjugate functions $\overline{f}_i$.)

The quality of this finite dimensional approximation of $\mathscr{U}$ depends on the selected dictionary of the observables (capturing a nearly invariant subspace that corresponds to the most relevant eigenvalues), as well as on the approximation level of the underlying measure by the empirical one, in particular on the distribution of the $\mathbf{z}_k$'s. For related numerical issues see[27] and for a theoretical study of convergence, see[5].

S1.3.3 Computation of eigenfunctions and the Koopman mode decomposition (KMD)

Next, we describe the framework for practical computation of the modal decomposition from §S1.2.1. It is the classical Rayleigh-Ritz extraction, based on (S24) and the spectral decomposition of $\mathbb{U}$.

Consider first the case $\operatorname{rank}(\mathbf{X}) = d$; then $d \le M+1$, and $\mathbb{U}$ is uniquely defined, column by column, from the solutions of the least squares problems (S22), for $i = 1,\ldots,d$. In this case all d eigenvalues (with the corresponding eigenvectors) are well determined by the data. For technical simplicity we assume that $\mathbb{U}$ is diagonalizable, with the spectral decomposition $\mathbb{U} = \mathbf{Q}\Lambda\mathbf{Q}^{-1}$, with $\Lambda = \operatorname{diag}(\lambda_i)_{i=1}^d$, $\mathbf{Q} = (\mathbf{q}_1,\ldots,\mathbf{q}_d)$, $\mathbb{U}\mathbf{q}_i = \lambda_i\mathbf{q}_i$. We do not assume that the eigenvalues are simple, and in the case of multiple eigenvalues we list them as successive diagonal entries of Λ. Then, for $z \in \mathscr{X}$,

$$\mathscr{U}\begin{pmatrix} f_1(z) & \ldots & f_d(z)\end{pmatrix}\mathbf{Q} = \begin{pmatrix} f_1(z) & \ldots & f_d(z)\end{pmatrix}\mathbf{Q}\Lambda + \begin{pmatrix} \rho_1(z) & \ldots & \rho_d(z)\end{pmatrix}\mathbf{Q}, \tag{S25}$$

and the approximate eigenfunctions of $\mathscr{U}$, extracted from the span of $f_1,\ldots,f_d$, are

$$\begin{pmatrix} \phi_1(z) & \ldots & \phi_d(z)\end{pmatrix} = \begin{pmatrix} f_1(z) & \ldots & f_d(z)\end{pmatrix}\mathbf{S} = \begin{pmatrix} \sum_{i=1}^d f_i(z)\mathbf{Q}_{i1} & \ldots & \sum_{i=1}^d f_i(z)\mathbf{Q}_{id}\end{pmatrix},\ (\mathscr{U}\phi_i)(z) = \lambda_i\phi_i(z) + \sum_{j=1}^d \rho_j(z)\mathbf{Q}_{ji}.$$

Following §S1.2.1, we seek a decomposition of observables in terms of the ϕ_i's, similar to (S12). In a numerical simulation, these eigenfunctions are accessible, as well as the observables, only as the tabulated values for $z \in \{\mathbf{z}_0,\ldots,\mathbf{z}_M\}$:

$$\begin{pmatrix} \phi_1(\mathbf{z}_0) & \phi_2(\mathbf{z}_0) & \phi_3(\mathbf{z}_0) & \ldots & \phi_d(\mathbf{z}_0) \\ \phi_1(\mathbf{z}_1) & \phi_2(\mathbf{z}_1) & \phi_3(\mathbf{z}_1) & \ldots & \phi_d(\mathbf{z}_1) \\ \vdots & \vdots & \vdots & \ldots & \vdots \\ \phi_1(\mathbf{z}_{M+1}) & \phi_2(\mathbf{z}_{M+1}) & \phi_3(\mathbf{z}_{M+1}) & \ldots & \phi_d(\mathbf{z}_{M+1}) \end{pmatrix} = \begin{pmatrix} f_1(\mathbf{z}_0) & f_2(\mathbf{z}_0) & f_3(\mathbf{z}_0) & \ldots & f_d(\mathbf{z}_0) \\ f_1(\mathbf{z}_1) & f_2(\mathbf{z}_1) & f_3(\mathbf{z}_1) & \ldots & f_d(\mathbf{z}_1) \\ \vdots & \vdots & \vdots & \ldots & \vdots \\ f_1(\mathbf{z}_{M+1}) & f_2(\mathbf{z}_{M+1}) & f_3(\mathbf{z}_{M+1}) & \ldots & f_d(\mathbf{z}_{M+1}) \end{pmatrix}\mathbf{Q} = \mathbf{S}^T\mathbf{Q}. \tag{S26}$$

Let now $\mathbf{g}(z)^T = (g_1(z),\ldots,g_d(z))$ be a vector valued observable and let $g_i(z) = \sum_{j=1}^d \gamma_{ji} f_j(z) + e_i(z)$, so that $\mathbf{g}(z)^T = (f_1(z),\ldots,f_d(z))\Gamma + E(z)$, $\Gamma = (\gamma_{ji}) \in \mathbb{C}^{d\times d}$, $E(z) = (e_1(z),\ldots,e_d(z))$. (If $g_i = f_i$, then $\Gamma = \mathbb{I}_d$ and $E = \mathbf{0}$. If $g_i \in \mathscr{F}_{\mathscr{D}}$, then $E = \mathbf{0}$.) Hence

$$\mathbf{g}(z)^T = \begin{pmatrix} f_1(z) & \ldots & f_d(z)\end{pmatrix}\mathbf{Q}\mathbf{Q}^{-1}\Gamma + E(z) = \begin{pmatrix} \phi_1(z) & \ldots & \phi_d(z)\end{pmatrix}\mathbf{Q}^{-1}\Gamma + E(z),\ z \in \mathscr{X}. \tag{S27}$$

Set $\mathbf{V} = \Gamma^T\mathbf{Q}^{-T} = \begin{pmatrix}\mathbf{v}_1 & \ldots & \mathbf{v}_d\end{pmatrix}$, where $\mathbf{v}_i$ is the ith column. Then

$$\begin{pmatrix} g_1(z) \\ \vdots \\ g_d(z)\end{pmatrix} = \underbrace{\Gamma^T\mathbf{Q}^{-T}}_{\mathbf{V}}\begin{pmatrix} \phi_1(z) \\ \vdots \\ \phi_d(z)\end{pmatrix} + E(z)^T = \sum_{i=1}^d \mathbf{v}_i\phi_i(z) + E(z)^T \approx \sum_{i=1}^d \mathbf{v}_i\phi_i(z).$$

Since $(\mathscr{U}\phi_i)(z) \approx \lambda_i\phi_i(z)$, we have

$$(\mathscr{U}_d^k\mathbf{g})(z) = \begin{pmatrix} (\mathscr{U}^k g_1)(z) \\ \vdots \\ (\mathscr{U}^k g_d)(z)\end{pmatrix} \approx \sum_{i=1}^d \mathbf{v}_i\phi_i(z)\lambda_i^k. \tag{S28}$$

In the sequel, we use $\Gamma = \mathbb{I}_d$; thus $\mathbf{V} = \mathbf{Q}^{-T}$. We can assume that $\|\mathbf{v}_i\|_2 = 1$, since $\mathbf{v}_i\phi_i(z) = (\mathbf{v}_i/\|\mathbf{v}_i\|_2)(\|\mathbf{v}_i\|_2\phi_i(z))$, where $\|\mathbf{v}_i\|_2\phi_i$ is again an eigenfunction. To evaluate (S28) numerically at $\mathbf{z}_0$, use (S26).

If some eigenvalues are multiple, then with any block diagonal nonsingular matrix $D = \bigoplus_k D_k$, that commutes with Λ, we have $\mathbb{U} = (\mathbf{Q}D)\Lambda(\mathbf{Q}D)^{-1}$. (The number of the blocks D_k equals the number of different eigenvalues, and the dimensions corresponds to their multiplicities.) If we repeat the same construction with $\widetilde{\mathbf{Q}} = \mathbf{Q}D$, then the new approximate eigenfunction of $\mathscr{U}$ are $(\widetilde{\phi}_1,\ldots,\widetilde{\phi}_d) = (\phi_1,\ldots,\phi_d)D$, and the matrix of the modes is $\widetilde{\mathbf{V}} = \mathbf{V}D^{-1}$. At the end, we obtain another representation of the sum in (S28).

Using (S23), we conclude that $\mathbb{A}\mathbf{Q}^{-T} = \mathbf{Q}^{-T}\Lambda$, i.e. the columns of $\mathbf{Q}^{-T}$ are the (right) eigenvectors of $\mathbb{A}$. Hence, for computing the Koopman modes, we can proceed with computing the eigenvectors of $\mathbb{A}$. The eigenvector matrix is necessarily of the form $\mathbf{Q}^{-T}D^{-1}$ with some $D = \bigoplus_k D_k$, as above.

Consider now the case $d > M+1 = \operatorname{rank}(\mathbf{X})$. We have $M+1 < d$ Ritz pairs of $\mathbb{U}$, and in the decomposition (S25) the matrix $\mathbf{Q}$ is tall rectangular, $d \times (M+1)$, so we cannot immediately insert $\mathbf{Q}\mathbf{Q}^{-1}$ as in (S27). To replace the spanning set $f_1,\ldots,f_d$ with $(\phi_1,\ldots,\phi_{M+1}) = (f_1,\ldots,f_d)\mathbf{Q}$, we must use $\mathbf{Q}\mathbf{Q}^{\dagger} \neq \mathbb{I}_d$. If the full column rank $\mathbf{Q}$ is extracted from the range of $\mathbf{X}$, then $\mathbf{Q}\mathbf{Q}^{\dagger}\mathbf{X} = \mathbf{X}$. We proceed with the assumption that the data snapshots are real – the additional goal is to point out that in that case all computation can be done (and in a software implementation it should) in real arithmetic, even if the eigenvalues

and eigenvectors (the columns of $\mathbf{Q}$) are complex. Since the matrix $\mathbb{U}$ is then real as well, the pair Λ, $\mathbf{Q}$ computed by the Rayleigh Ritz method will be closed under conjugation and can be indexed as follows: if $\lambda_i \in \mathbb{R}$, then $\mathbf{q}_i \in \mathbb{R}^d$, and if $\Im(\lambda_i) > 0$, then $\lambda_{i+1} = \overline{\lambda}_i$, $\mathbf{q}_{i+1} = \overline{\mathbf{q}}_i$. Using the identity

$$\begin{pmatrix}\mathbf{q}_i & \overline{\mathbf{q}}_i\end{pmatrix}\begin{pmatrix}1 & -\mathrm{i}\\ 1 & \mathrm{i}\end{pmatrix} = \begin{pmatrix}2\Re(\mathbf{q}_i) & 2\Im(\mathbf{q}_i)\end{pmatrix}$$

we immediately conclude that $\mathbf{Q} = \widetilde{\mathbf{Q}}J$, where $\widetilde{\mathbf{Q}}$ is real and J nonsingular. (Here $\Re(\cdot)$ and $\Im(\cdot)$ denote the real and the imaginary parts of complex scalars or vectors.) Hence, $\mathbf{Q}\mathbf{Q}^\dagger = \widetilde{\mathbf{Q}}\widetilde{\mathbf{Q}}^\dagger$ is real symmetric and $\mathbf{X}^T\mathbf{Q}\mathbf{Q}^\dagger = \mathbf{X}^T$. On the other hand, in a practical computation, we see the function values only at $z \in \{\mathbf{z}_0, \ldots, \mathbf{z}_{M+1}\}$, and for those values we can use $\mathbf{Q}\mathbf{Q}^\dagger$ instead of $\mathbf{Q}\mathbf{Q}^{-1}$ in relation (S27). The rest is straightforward, yielding the modal matrix $\mathbf{V} = \Gamma^T\mathbf{Q}^{\dagger T}$, and $\mathbf{Q}\mathbf{Q}^\dagger\mathbb{A}\mathbf{Q}^{\dagger T} = \mathbf{Q}^{\dagger T}\Lambda$. The latter reveals that $\mathbf{Q}^\dagger$, Λ correspond to Ritz pairs of $\mathbb{A}$, extracted from the range of $\mathbf{X}$.
In the next section, we derive the KMD directly from an application of the Rayleigh Ritz procedure to the matrix $\mathbb{A}$.

S1.3.4 Krylov compression of $\mathscr{U}_d$ and the KMD

Note that, for an $\mathbf{f} \in \mathscr{F}$, (S14) naturally generates a Krylov sequence of functions $\mathbf{f}, \mathscr{U}_d\mathbf{f}, \ldots, \mathscr{U}_d^M\mathbf{f}, \mathscr{U}_d^{M+1}\mathbf{f}$, and that

$$\mathscr{U}_d \underbrace{\begin{pmatrix}\mathbf{f} & \mathscr{U}_d\mathbf{f} & \mathscr{U}_d^2\mathbf{f} & \ldots & \mathscr{U}_d^M\mathbf{f}\end{pmatrix}}_{\mathscr{K}_{M+1}} = \begin{pmatrix}\mathbf{f} & \mathscr{U}_d\mathbf{f} & \mathscr{U}_d^2\mathbf{f} & \ldots & \mathscr{U}_d^M\mathbf{f}\end{pmatrix} C_{M+1} + E_{M+1}, \tag{S29}$$

$$\mathscr{U}_d\mathscr{K}_{M+1} = \mathscr{K}_{M+1}C_{M+1} + E_{M+1}, \quad C_{M+1} = \begin{pmatrix} 0 & 0 & 0 & 0 & \alpha_0\\ 1 & 0 & 0 & 0 & \alpha_1\\ 0 & 1 & 0 & 0 & \alpha_2\\ 0 & 0 & 1 & 0 & \alpha_3\\ 0 & 0 & 0 & 1 & \alpha_M\end{pmatrix}, \tag{S30}$$

where we have written $\mathscr{K}_{M+1} = \begin{pmatrix}\mathbf{f} & \mathscr{U}_d\mathbf{f} & \mathscr{U}_d^2\mathbf{f} & \ldots & \mathscr{U}_d^M\mathbf{f}\end{pmatrix}$,

$$\mathscr{U}_d^{M+1}\mathbf{f} = \sum_{i=0}^{M} \alpha_i \mathscr{U}_d^i\mathbf{f} + \mathbf{r}_{M+1}, \tag{S31}$$

and $E_{M+1} = \begin{pmatrix}\mathbf{0} & \mathbf{r}_{M+1}\end{pmatrix}$. In (S31), $\mathbf{r}_{M+1}$ is the residual obtained after projecting $\mathscr{U}_d^{M+1}\mathbf{f}$ onto the subspace spanned by $[\mathscr{K}_{M+1}] = \mathrm{span}\{\mathscr{U}_d^i\mathbf{f},\ i = 0, \ldots, M\}$. Here we assume that $M+1 < d$ (possibly even $M+1 \ll d$), so that we expect nonzero residual $\mathbf{r}_{M+1}$. Our earlier full rank assumption on $\mathbf{X}$ implies that its rank is $M+1$.

If $\mathbb{P}_{M+1}$ is the orthogonal projector onto $[\mathscr{K}_{M+1}]$, then the compression $\mathbb{P}_{M+1}\mathscr{U}_{d|[\mathscr{K}_{M+1}]}$ is represented by the matrix C_{M+1}. If $C_{M+1}\mathbf{v} = \lambda\mathbf{v}$, where $\mathbf{v} = (v_1, \ldots, v_{M+1})^T \neq \mathbf{0}$, then

$$\mathscr{U}_d(\sum_{i=0}^{M} v_{i+1}\mathscr{U}_d^i\mathbf{f}) = \lambda(\sum_{i=0}^{M} v_{i+1}\mathscr{U}_d^i\mathbf{f}) + v_{M+1}\mathbf{r}_{M+1}. \tag{S32}$$

This means that λ and the function $\mathbf{h} = \sum_{i=0}^{M} v_{i+1}\mathscr{U}_d^i\mathbf{f} = \mathscr{K}_{M+1}\mathbf{v}$ satisfy $\mathscr{U}_d\mathbf{h} = \lambda\mathbf{h} + v_{M+1}\mathbf{r}_{M+1}$, i.e. $(\lambda, \mathbf{h})$ is an approximate eigenpair with the residual

$$\|\mathscr{U}_d\mathbf{h} - \lambda\mathbf{h}\| = |v_{M+1}|\|\mathbf{r}_{M+1}\| \tag{S33}$$

measured in the norm of the function space $\mathscr{F}$.

Given the data snapshots (S15) as the only available numerical information, the coefficients $\alpha = (\alpha_1, \ldots, \alpha_M)$ in (S31) can be determined using the discretized (algebraic) least squares projection and the notation from §S1.3.1 as follows: The least squares error to be minimized is

$$\|(\mathscr{U}_d^{M+1}\mathbf{f})(\mathbf{z}_0) - \sum_{i=0}^{M} \alpha_i(\mathscr{U}_d^i\mathbf{f})(\mathbf{z}_0)\|_2^2 = \|\mathbf{f}(\mathbf{z}_{M+1}) - \sum_{i=0}^{M} \alpha_i\mathbf{f}(\mathbf{z}_i)\|_2^2 = \|\mathbf{y}_{M+1} - \mathbf{X}\alpha\|_2^2. \tag{S34}$$

If $\mathbf{X}$ is of full column rank, then $\alpha = \mathbf{X}^\dagger\mathbf{y}_{M+1}$ is the unique solution expressed using the Moore-Penrose pseudoinverse. Hence, for a particular initial $\mathbf{z}_0$, the relation (S30) reads

$$(\mathscr{U}_d\mathscr{K}_{M+1})(\mathbf{z}_0) = \mathbf{Y} = \mathscr{K}_{M+1}(\mathbf{z}_0)C_{M+1} + (\mathbf{y}_{M+1} - \mathbf{X}\mathbf{X}^\dagger\mathbf{y}_{M+1})\mathbf{e}_{M+1}^T \quad \mathbf{e}_{M+1}^T = \begin{pmatrix}0 & \ldots & 0, 1\end{pmatrix}. \tag{S35}$$

On the other hand, by (S16), $(\mathscr{U}_d\mathscr{K}_{M+1})(\mathbf{z}_0) = \mathbb{A}\mathscr{K}_{M+1}(\mathbf{z}_0)$, and, as a concrete numerical realization of (S30) on the trajectory starting at $\mathbf{z}_0$, we obtain the Krylov decomposition

$$\mathbf{Y} = \mathbb{A}\mathbf{X} = \mathbf{X}C_{M+1} + (\mathbf{y}_{M+1} - \mathbf{X}\mathbf{X}^\dagger\mathbf{y}_{M+1})\mathbf{e}_{M+1}^T. \tag{S36}$$

where $C_{M+1} = \mathbf{X}^\dagger \mathbb{A}\mathbf{X} = \mathbf{X}^\dagger \mathbf{Y}$ is the Rayleigh quotient. Note here that the full column rank assumption on $\mathbf{X}$ implies $\mathbf{X}^\dagger\mathbf{X} = \mathbb{I}$. Also note that here we do not have $\mathbb{A}$ explicitly formed, nor we think of it as $\mathbb{A} = \mathbf{Y}\mathbf{X}^\dagger$.

Hence, since the residual $\widehat{\mathbf{r}}_{M+1} = \mathbf{y}_{M+1} - \mathbf{X}\mathbf{X}^\dagger\mathbf{y}_{M+1}$ is unlikely to be zero, we can extract from $\mathbf{X}$ only approximate (Ritz) eigenpairs of $\mathbb{A}$. To that end, we first compute the eigenvalues and eigenvectors of C_{M+1}. Under the generic assumption that all eigenvalues of C_{M+1} are algebraically simple,[1] its spectral decomposition is $C_{M+1} = \mathbb{V}_{M+1}^{-1}\Lambda_{M+1}\mathbb{V}_{M+1}$, where

$$\Lambda_{M+1} = \begin{pmatrix} \lambda_1 & & \\ & \ddots & \\ & & \lambda_{M+1} \end{pmatrix}, \quad \mathbb{V}_{M+1} = \begin{pmatrix} 1 & \lambda_1 & \dots & \lambda_1^M \\ 1 & \lambda_2 & \dots & \lambda_2^M \\ \vdots & \vdots & \dots & \vdots \\ 1 & \lambda_{M+1} & \dots & \lambda_{M+1}^M \end{pmatrix}, \quad \det(\mathbb{V}_{M+1}) \equiv \prod_{j>k}(\lambda_j - \lambda_k) \neq 0. \tag{S37}$$

In other words, the eigenvectors of C_{M+1} are the columns of the inverse of the Vandermonde matrix $\mathbb{V}_{M+1}$. From $\mathbb{A}(\mathbf{X}\mathbb{V}_{M+1}^{-1}) = (\mathbf{X}\mathbb{V}_{M+1}^{-1})\Lambda_{M+1} + \widehat{\mathbf{r}}_{M+1}e^T\mathbb{V}_{M+1}^{-1} \approx (\mathbf{X}\mathbb{V}_{M+1}^{-1})\Lambda_{M+1}$, we see that the columns $\widehat{\mathbf{v}}_i$ of $\widehat{\mathbf{V}} = \mathbf{X}\mathbb{V}_{M+1}^{-1} = (\widehat{\mathbf{v}}_1, \dots, \widehat{\mathbf{v}}_{M+1})$ are approximate eigenvectors of $\mathbb{A}$. With an eye towards (S28), we write $\mathbf{X} = \widehat{\mathbf{V}}\mathbb{V}_{M+1}$, i.e., for $k = 0, \dots, M$,

$$(\mathscr{U}_d^k\mathbf{f})(\mathbf{z}_0) = \mathbf{X}e_{k+1} = \sum_{i=1}^{M+1} \frac{\widehat{\mathbf{v}}_i}{\|\widehat{\mathbf{v}}_i\|_2}\|\widehat{\mathbf{v}}_i\|_2\lambda_i^k = \sum_{i=1}^{M+1} \mathbf{v}_i\|\widehat{\mathbf{v}}_i\|_2\lambda_i^k. \tag{S38}$$

It is precisely this structure that yields the spatio-temporal representation in §S1.2.1. Indeed, if we set

$$\Phi = \begin{pmatrix} \mathbf{f} & \mathscr{U}_d\mathbf{f} & \mathscr{U}_d^2\mathbf{f} & \dots & \mathscr{U}_d^M\mathbf{f} \end{pmatrix} \mathbb{V}_{M+1}^{-1} = \mathscr{K}_{M+1}\mathbb{V}_{M+1}^{-1},$$

then λ_j and the jth column $\Phi_{:j}$ of Φ are a Ritz pair of $\mathscr{U}_d$, $\mathscr{U}_d\Phi_{:j} \approx \lambda_j\Phi_{:j}$, see (S32), (S33). If $\Phi_{:j} = (\varphi_{1j}, \dots, \varphi_{dj})^T$, then $\mathscr{U}\varphi_{ij} \approx \lambda_j\varphi_{ij}$, $i = 1, \dots, d$. We have for $k = 0, \dots, M$

$$\mathscr{U}_d^k\mathbf{f}(\mathbf{z}_0) = \begin{pmatrix} \mathscr{U}^k f_1(\mathbf{z}_0) \\ \vdots \\ \mathscr{U}^k f_d(\mathbf{z}_0) \end{pmatrix} = \sum_{i=1}^{M+1} \Phi_{:i}\lambda_i^k = \sum_{i=1}^{M+1} \begin{pmatrix} \varphi_{1i}(\mathbf{z}_0) \\ \vdots \\ \varphi_{di}(\mathbf{z}_0) \end{pmatrix} \lambda_i^k, \tag{S39}$$

and we can extrapolate this to the future steps by increasing k which amounts to rising the powers of λ_i. Also note that Φ evaluated at $\mathbf{z}_0$ equals precisely $\widehat{\mathbf{V}}$, so that (S38) is a concrete numerical realization of (S39). In an ideal situation, λ_j is geometrically simple eigenvalue and φ_{ij} are nearly collinear for $i = 1, \dots, d$. However, this is not essential for the purposes of snapshots representation and prediction because the action of $\mathscr{U}_d$ is component-wise, and each φ_{ij} is an approximate eigenfunction of $\mathscr{U}$.

This algebraically elegant process has a drawback that becomes apparent when we consider its numerical software implementation. Vandermonde matrices are notoriously ill-conditioned. Moreover, in case of an off–attractor analysis the values $|\lambda_i|^j$ may vary in size over several orders of magnitude, which poses challenging problems for the finite precision computation. For that reason, an SVD based method of Schmid[28], designated as DMD, has become the main computational device for the KMD. However, we have recently shown in[13] that this companion matrix based approach can be implemented more accurately using the DFT and specially tailored algorithms for the Vandermonde and the related Cauchy matrices.

S1.3.5 Schmid's dynamic mode decomposition (DMD)

The Rayleigh-Ritz procedure outlined in §S1.3.4 is based on a Krylov sequence, which naturally fits the dynamics of a discrete dynamical system driven by $\mathscr{U}$ (in the space of observables). However, it yields a numerically ill-conditioned problem, as a consequence of that very representation. From a numerical point of view, the Rayleigh-Ritz procedure is best executed in unitary/orthonormal bases, so that $\mathbf{X}$ should be replaced with an orthonormal matrix spanning the same subspace. Since $\mathbf{X}$ can be nearly numerically rank deficient (its columns are actually generated by the power method), Schmid[28] used the PCA[29] with prescribed cutoff threshold to construct the best lower dimensional subspace (i.e. a POD basis) that captures the data, and then used the Rayleigh-Ritz extraction from that subspace. For the readers' convenience, we briefly review the DMD algorithm; we assume the more general setting where the snapshots are generated with several initial conditions, so that the input data are not necessarily of the form (S14). That is, the matrices $\mathbf{X}$ and $\mathbf{Y}$ are such that, column-wise, $\mathbf{x}_k = \mathbf{f}(\mathbf{z}_k)$, $\mathbf{y}_k = \mathbf{f}(\mathbf{T}(\mathbf{z}_k)) = \mathbb{A}\mathbf{x}_k$; see[26]. The total number of snapshots (column dimension) is in this general case denoted by m; in the case of a single trajectory (S14), $m = M + 1$.

The theoretical underpinning is the classical matrix theorem on best low rank approximations.

[1] Since C_{M+1} is an unreduced Hessenberg matrix, its eigenvalues must be of geometric multiplicity one. If C_{M+1} has multiple eigevaules, then its generalized eigenvector matrix is the inverse of the confluent Vandermonde matrix generated by the distinct eigenvalues. The Jordan structure of each multiple eigenvalue consists of a single Jordan block.

Theorem S1.2 *(Eckart-Young[30], Mirsky[31]) Let the SVD of* $\mathbf{X} \in \mathbb{C}^{d\times m}$ *be*

$$\mathbf{X} = U\Sigma\mathscr{V}^*, \;\; \Sigma = \mathrm{diag}(\sigma_i)_{i=1}^{\min(d,m)}, \;\; \sigma_1 \geq \cdots \geq \sigma_{\min(d,m)} \geq 0.$$

For $r \in \{1,\ldots,\mathrm{rank}(\mathbf{X})\}$*, define* $U_r = U(:,1:r)$*,* $\Sigma_r = \Sigma(1:r,1:r)$*,* $\mathscr{V}_r = \mathscr{V}(:,1:r)$*, and* $\mathbf{X}_r = U_r\Sigma_r\mathscr{V}_r^*$*. Then,* $\mathbf{X}_r$ *is closest matrix of rank at most* r *to* $\mathbf{X}$*, in* $\|\cdot\|_2$ *and the Frobenius norm* $\|\cdot\|_F$*, i.e.*

$$\min_{\mathrm{rank}(\Xi)\leq r} \|\mathbf{X}-\Xi\|_2 = \|\mathbf{X}-\mathbf{X}_r\|_2 = \sigma_{r+1}; \quad \min_{\mathrm{rank}(\Xi)\leq r} \|\mathbf{X}-\Xi\|_F = \|\mathbf{X}-\mathbf{X}_r\|_F = \sqrt{\sum_{i=r+1}^{\min(d,m)} \sigma_i^2}. \tag{S40}$$

Hence, we can replace $\mathbf{X}$ with its best low rank approximation by truncating its SVD $\mathbf{X} = U\Sigma\mathscr{V}^* \approx U_r\Sigma_r\mathscr{V}_r^*$, where $U_r = U(:,1:r)$ is $d\times r$ orthonormal ($U_r^*U_r = \mathbb{I}_r$), $\mathscr{V}_r = \mathscr{V}(:,1:r)$ is $m\times r$, also orthonormal ($\mathscr{V}_r^*\mathscr{V}_r = \mathbb{I}_r$), and $\Sigma_r = \mathrm{diag}(\sigma_i)_{i=1}^r$ contains the largest r singular values of $\mathbf{X}$. In brief, U_r is the POD basis for the snapshots $\mathbf{x}_1,\ldots,\mathbf{x}_m$,

$$\sum_{i=1}^m \|\mathbf{x}_i - U_rU_r^*\mathbf{x}_i\|_2^2 = \min_{\Theta^*\Theta=\mathbb{I}_r} \sum_{i=1}^m \|\mathbf{x}_i - \Theta\Theta^*\mathbf{x}_i\|_2^2.$$

The index r is selected so that the approximation error (S40) is below a user prescribed threshold value, and it is a numerical rank[32] of $\mathbf{X}$. Now, DMD uses the range of U_r for the Rayleigh-Ritz extraction. The Rayleigh quotient $A_r = U_r^*\mathbb{A}U_r$ is computed, using

$$\mathbf{Y} = \mathbb{A}\mathbf{X} \approx \mathbb{A}U_r\Sigma_r\mathscr{V}_r^*, \;\; \text{and} \;\; \mathbb{A}U_r = \mathbf{Y}\mathscr{V}_r\Sigma_r^{-1}, \tag{S41}$$

as

$$A_r = U_r^*\mathbf{Y}\mathscr{V}_r\Sigma_r^{-1}, \tag{S42}$$

which is suitable for data driven setting because it does not use $\mathbb{A}$ explicitly. Clearly, (S41, S42) only require that $\mathbf{Y} = \mathbb{A}\mathbf{X}$; it is not necessary that $\mathbf{Y}$ is shifted $\mathbf{X}$ as in §S1.3.4. Each eigenpair (λ, w) of A_r generates the corresponding Ritz pair (λ, U_rw) for $\mathbb{A}$. This is the essence of the Schmid's method[28], summarized in Algorithm S1 below.

Algorithm S1 $[\mathbf{V}_r,\Lambda_r] = \mathrm{DMD}(\mathbf{X},\mathbf{Y})$

Require: • $\mathbf{X} = (\mathbf{x}_1,\ldots,\mathbf{x}_m), \mathbf{Y} = (\mathbf{y}_1,\ldots,\mathbf{y}_m) \in \mathbb{C}^{d\times m}$ that define a sequence of snapshots pairs $(\mathbf{x}_k, \mathbf{y}_k \equiv \mathbb{A}\mathbf{x}_k)$. (Tacit assumption is that d is large and that $m \ll d$.)

1: $[U,\Sigma,\mathscr{V}] = svd(\mathbf{X})$; ▷ *The thin SVD:* $\mathbf{X} = U\Sigma\mathscr{V}^*$, $U \in \mathbb{C}^{d\times m}$, $\Sigma = \mathrm{diag}(\sigma_i)_{i=1}^m$, $\mathscr{V} \in \mathbb{C}^{m\times m}$.

2: Determine numerical rank r ;

3: Set $U_r = U(:,1:r)$; $\mathscr{V}_r = \mathscr{V}(:,1:r)$; $\Sigma_r = \Sigma(1:r,1:r)$;

4: $A_r = ((U_r^*\mathbf{Y})\mathscr{V}_r)\Sigma_r^{-1}$; ▷ *Schmid's formula for the Rayleigh quotient* $U_r^*\mathbb{A}U_r$.

5: $[\mathbf{W}_r,\Lambda_r] = \mathrm{eig}(A_r)$; ▷ $\Lambda_r = \mathrm{diag}(\lambda_i)_{i=1}^r$; $A_r\mathbf{W}_r(:,i) = \lambda_i\mathbf{W}_r(:,i)$; $\|\mathbf{W}_r(:,i)\|_2 = 1$

6: $\mathbf{V}_r = U_r\mathbf{W}_r$. ▷ *Ritz vectors.*

Ensure: $\mathbf{V}_r = (\mathbf{v}_1 \ldots \mathbf{v}_r)$, Λ_r .

Schmid's DMD algorithm has been notably successful in CFD applications. For more on interesting applications and modifications of the DMD, see e.g.[11,33,34,35,36,37,38,39,40,41,42,43].

S1.3.6 Refined Rayleigh-Ritz Data Driven Modal Decomposition (RRRDDMD)

Recently, in[12], we revisited DMD and introduced several modifications. First, we show that the residuals $\|\mathbb{A}\mathbf{v}_i - \lambda_i\mathbf{v}_i\|_2$ can be computed in a data driven scenario as well. This allows for selecting good Ritz pairs, with small residuals, which proved to be the key for selecting good modes for the prediction algorithm; see §S1.4.3. Further, we show that the Ritz vectors can be improved by using the well known refinement technique, which we have adapted to the data driven setting of the DMD.

S1.3.7 Spatio-temporal representation of the snapshots

In general, a DMD algorithm will compute $r \leq m$ Ritz vectors (modes) with the corresponding eigenvalues. In particular, in the Schmid's DMD, r may be considerably smaller than m, as e.g. in the case of an off-attractor analysis of a dynamical systems, after removing peripheral eigenvalues, see [12, §4.1]. In any case, the most important coherent structures of the process are determined by a subset of the modes; so we may want to express the available data snapshots by $r < m$ modes. It is desirable

Algorithm S2 $[\mathbf{V}_r, \Lambda_r, \mathrm{rez}_r, \rho_r] = \mathrm{DDMD\_RRR}(\mathbf{X}, \mathbf{Y}; \varepsilon)$ {*Refined Rayleigh-Ritz Data Driven Modal Decomposition*[12]}

Require:

- $\mathbf{X} = (\mathbf{x}_1, \ldots, \mathbf{x}_m), \mathbf{Y} = (\mathbf{y}_1, \ldots, \mathbf{y}_m) \in \mathbb{C}^{d\times m}$ that define a sequence of snapshots pairs $(\mathbf{x}_k, \mathbf{y}_k \equiv \mathbb{A}\mathbf{x}_k)$. (Tacit assumption is that d is large and that $m \ll d$.)
- Tolerance level ε for numerical rank determination.

$\mathbf{D}_x = \mathrm{diag}(\|\mathbf{X}(:,i)\|_2)_{i=1}^m$; $\mathbf{X}^{(1)} = \mathbf{X}\mathbf{D}_x^\dagger$; $\mathbf{Y}^{(1)} = \mathbf{Y}\mathbf{D}_x^\dagger$;
$[U, \Sigma, \mathscr{V}] = svd(\mathbf{X}^{(1)})$; ▷ *The thin SVD:* $\mathbf{X}^{(1)} = U\Sigma\mathscr{V}^*$, $U \in \mathbb{C}^{d\times m}$, $\Sigma = \mathrm{diag}(\sigma_i)_{i=1}^m$.
Determine numerical rank r, with the threshold ε. See [12, §3.1.1] .
Set $U_r = U(:,1:r)$; $\mathscr{V}_r = \mathscr{V}(:,1:r)$; $\Sigma_r = \Sigma(1:r,1:r)$;
$B_r = \mathbf{Y}^{(1)}(\mathscr{V}_r\Sigma_r^{-1})$; ▷ *Schmid's data driven formula for* $\mathbb{A}U_r$.
$[Q_r, R] = qr(\begin{pmatrix} U_r & B_r \end{pmatrix})$; ▷ *The thin QR factorization:* $\begin{pmatrix} U_r & B_r \end{pmatrix} = Q_r R$; Q_r *not computed.*
$A_r = \mathrm{diag}(\overline{R_{ii}})_{i=1}^r R(1:r, r+1:2r)$; ▷ $A_r = U_r^*\mathbb{A}U_r$ *is the Rayleigh quotient.*
$\Lambda_r = \mathrm{eig}(A_r)$ ▷ $\Lambda_r = \mathrm{diag}(\lambda_i)_{i=1}^r$; *Ritz values, i.e. eigenvalues of* A_r.
for $i = 1, \ldots, r$ **do**
  $[\sigma_{\lambda_i}, w_{\lambda_i}] = svd_{\min}(\begin{pmatrix} R(1:r,r+1:2r) - \lambda_i R(1:r,1:r) \\ R(r+1:2r,r+1:2r) \end{pmatrix})$; ▷ *Min. singular value and the corr. right sing. vector, see [12, §3.3].*
  $\mathbf{W}_r(:,i) = w_{\lambda_i}$; $\mathrm{rez}_r(i) = \sigma_{\lambda_i}$; ▷ *Optimal residual.*
  $\rho_r(i) = w_{\lambda_i}^* A_r w_{\lambda_i}$; ▷ *Rayleigh quotient,* $\rho_r(i) = (U_r w_{\lambda_i})^*\mathbb{A}(U_r w_{\lambda_i})$.
end for
$\mathbf{V}_r = U_r\mathbf{W}_r$; ▷ *Refined Ritz vectors.*

Ensure: $\mathbf{V}_r = (\mathbf{v}_1 \ \ldots \ \mathbf{v}_r)$, Λ_r, rez_r, ρ_r.

that such modes can represent the snapshots reasonably well, and that they have small residuals which, as we shall see below, is essential for the prediction of the evolution of the sequence (S14), with $m = M+1$. Assume that we have such a selection of r numerically linearly independent modes and, to ease the notation, assume that we have indexed the Ritz pairs so that the selected ones are indexed with $j = 1, \ldots, r$. With this setup, a modal decomposition of $\mathbf{f}_k = \mathbf{f}(\mathbf{z}_k)$ can be written as

$$\mathbf{f}_k \approx \sum_{j=1}^{r} \lambda_j^k \alpha_j \mathbf{v}_j, \quad k = 0, \ldots, M+1. \tag{S43}$$

If $r = M+1 = m$, then the coefficients $\alpha_1, \ldots, \alpha_m$ can be computed as

$$(\alpha_j)_{j=1}^m = \mathbf{V}_m^\dagger \mathbf{f}_0, \tag{S44}$$

so that this reconstruction is exact for $k = 0, \ldots, M$. In matrix notation, if we define $\mathbf{V}_r = \begin{pmatrix} \mathbf{v}_1 & \ldots & \mathbf{v}_r \end{pmatrix}$ then we have

$$\begin{pmatrix} \mathbf{f}_0 & \mathbf{f}_1 & \ldots & \mathbf{f}_{M+1} \end{pmatrix} \approx \begin{pmatrix} \mathbf{v}_1 & \mathbf{v}_2 & \ldots & \mathbf{v}_r \end{pmatrix} \begin{pmatrix} \alpha_1 & & & \\ & \alpha_2 & & \\ & & \ddots & \\ & & & \alpha_r \end{pmatrix} \begin{pmatrix} 1 & \lambda_1 & \ldots & \lambda_1^{M+1} \\ 1 & \lambda_2 & \ldots & \lambda_2^{M+1} \\ \vdots & \vdots & \ldots & \vdots \\ 1 & \lambda_r & \ldots & \lambda_r^{M+1} \end{pmatrix} \equiv \mathbf{V}_r D_\alpha \mathbb{V}_{r,M+2}. \tag{S45}$$

To compensate for the truncation error, the coefficients α_j can be recomputed by solving the weighted least squares problem

$$(\alpha_1, \ldots, \alpha_r) = \mathrm{argmin}_{\alpha_j} \sum_{k=0}^{M+1} w_k^2 \|\sqrt{\Omega}(\mathbf{f}_k - \sum_{j=1}^{r} \mathbf{v}_j \alpha_j \lambda_j^k)\|_2^2, \tag{S46}$$

where $w_k \geq 0$ are the weights that can be used to emphasize importance of some time indicies or to introduce forgetting factors, Ω is positive definite matrix,[2] and $\sqrt{\Omega}$ stands for the positive definite square root or the Cholesky factor of Ω. For numerical methods for this optimization problem we refer to[44],[14]. Here, for the reader's convenience, we provide an explicit formula for $\Omega = \mathbb{I}_d$:

$$(\alpha_1, \ldots, \alpha_r)^T = [(\mathbf{V}_r^*\mathbf{V}_r) \odot (\overline{\mathbb{V}_{r,M+2}\mathbf{W}^2\mathbb{V}_{r,M+2}^*})]^{-1}[(\overline{\mathbb{V}_{r,M+2}\mathbf{W}} \odot (\mathbf{V}_r^*\mathbf{X}\mathbf{W}))\overrightarrow{\mathbf{1}}], \tag{S47}$$

where $\mathbf{W} = \mathrm{diag}(w_k)$, $\overrightarrow{\mathbf{1}} = (1, 1, \ldots, 1)^T$, and $\odot$ denotes the Hadamard matrix product; see [14, §3.2].

[2] In fact, we allow also a diagonal semidefinite matrix Ω as a mean to exclude selected components of the $\mathbf{f}_k$'s from the minimization (S46).

S1.4 Global Koopman prediction algorithm

Here we give the details of the new proposed algorithm, designated as *Global Koopman Prediction* (GKP) algorithm. The basic idea is to extract the intrinsic eigenvalues and modes of the dynamical system under consideration, and then to predict the evolution of the system by using the principles outlined in §S1.2.1, §S1.3.3. In order to reveal the relevant eigenvalues and the corresponding modes that capture the dynamics of the system on a larger time interval and not only locally, one has to use large training sets. This strategy is at risk if the algorithm is oblivious to unusual and unexpected changes (perturbations) that can be classified as Black Swan events. If such data are used in a learning window, the long term prediction is doomed to fail. We use the numerically computed spectral information on $\mathscr{U}$ to develop an additional device to equip the algorithm with a *litmus test* for detecting Black Swan events (a posteriori, of course), and, moreover, with a retouching scheme to restore the global prediction capability (see §2.2 in the main text). Furthermore, an important feature is that the data snapshots are lifted in a Hankel matrix structure, as described in §S1.4.2.

S1.4.1 Setting the scene - the prediction task

Consider a discrete dynamical system $\mathbf{z}_{k+1} = \mathbf{T}(\mathbf{z}_k)$ that is accessible through a sequence of snapshots

$$\mathbf{f}_0, \mathbf{f}_1, \mathbf{f}_2, \ldots \quad (\mathbf{f}_k \in \mathbb{R}^d, \; k = 0, 1, 2, \ldots, M, M+1, \ldots) \tag{S48}$$

where $d \geq 1$ is the dimension of the scalar or vector-valued *system observable* $\mathbf{f} : \mathscr{X} \to \mathbb{R}^d$, and $\mathbf{f}_k = \mathbf{f}(\mathbf{z}_k)$ is its value for the (possibly unknown) state $\mathbf{z}_k$, with the time stamp t_k, $k = 0, 1, 2, \ldots$. The goal is to learn the dynamics from the available data and then to predict the future values.

More precisely, suppose that the *present time moment* is t_{p-1}, and that, up to that moment, the data is readily available; we seek a prediction of the data value at the next time moments $t_p, t_{p+1}, \ldots, t_{p+\tau(p)}$. We call that future time moments t_p the *prediction moments*. The prediction will be based on a *sliding window* of size w in the sequence (S48), i.e. we will use the $\mathbf{f}_k$'s starting from the index $b = p - \mathsf{w}$ that defines the *active window* $\mathscr{W}(p, \mathsf{w})$ of consecutive data (*training set*) with indices $b = p - \mathsf{w}, b+1, \ldots, b+\mathsf{w}-1 = p-1$. In terms of the system mapping $\mathbf{T}$, these values can be represented as

$$\mathbf{f}_b = (\mathbf{f} \circ \mathbf{T}^b)(\mathbf{z}_0),\; \mathbf{f}_{b+1} = (\mathbf{f} \circ \mathbf{T}^{b+1})(\mathbf{z}_0), \ldots,\; \mathbf{f}_{b+\mathsf{w}-1} = (\mathbf{f} \circ \mathbf{T}^{b+\mathsf{w}-1})(\mathbf{z}_0). \tag{S49}$$

S1.4.2 Lifting the data into a Hankel matrix structure and the H-DMD

The key for a successful application of the prediction framework from §S1.2.1 is that the finite dimensional numerical approximation from §S1.3.3 captures the spectral information accurately enough. To that end, we adopt the Hankel-DMD approach of[45],[46]. For an active window $\mathscr{W}(p, \mathsf{w})$, first conveniently split $\mathsf{w} = m_H + n_H$, and then lift the observables into the higher dimensional space $\mathbb{R}^\ell$, $\ell = d \cdot n_H$, and arrange them as columns of a $\ell \times (m_H + 1)$ (block) Hankel matrix as follows:

$$\mathbb{H} = \begin{pmatrix} \mathbf{f}_b & \mathbf{f}_{b+1} & \cdots & \mathbf{f}_{b+m_H-1} & \mathbf{f}_{b+m_H} \\ \mathbf{f}_{b+1} & \mathbf{f}_{b+2} & \cdots & \mathbf{f}_{b+m_H} & \mathbf{f}_{b+m_H+1} \\ \vdots & \vdots & \ddots & \vdots & \vdots \\ \mathbf{f}_{b+n_H-1} & \mathbf{f}_{b+n_H} & \cdots & \mathbf{f}_{b+n_H+m_H-2} & \mathbf{f}_{b+n_H+m_H-1} \end{pmatrix} = \begin{pmatrix} \mathbf{h}_1 & \mathbf{h}_2 & \ldots & \mathbf{h}_{m_H+1} \end{pmatrix}. \tag{S50}$$

We can think of the $\mathbf{h}_i$'s as the values of the vector-valued observable $\mathbf{h} : \mathscr{X} \longrightarrow \mathbb{R}^\ell$ composed with the powers of $\mathbf{T}$ analogously to (S49), i.e. $\mathbf{h} = \begin{pmatrix} \mathbf{f} \circ \mathbf{T}^b & \mathbf{f} \circ \mathbf{T}^{b+1} & \ldots & \mathbf{f} \circ \mathbf{T}^{b+n_H-1} \end{pmatrix}^T$ and

$$\begin{pmatrix} \mathbf{h}_1 & \mathbf{h}_2 & \mathbf{h}_3 & \ldots & \mathbf{h}_{m_H+1} \end{pmatrix} = \begin{pmatrix} \mathbf{h}(\mathbf{z}_b) & \mathbf{h} \circ \mathbf{T}(\mathbf{z}_b) & \mathbf{h} \circ \mathbf{T}^2(\mathbf{z}_b) & \ldots & \mathbf{h} \circ \mathbf{T}^{m_H}(\mathbf{z}_b) \end{pmatrix} \tag{S51}$$

$$= \begin{pmatrix} \mathbf{h}(\mathbf{z}_b) & (\mathscr{U}_\ell \mathbf{h})(\mathbf{z}_b) & (\mathscr{U}_\ell^2 \mathbf{h})(\mathbf{z}_b) & \ldots & (\mathscr{U}_\ell^{m_H} \mathbf{h})(\mathbf{z}_b) \end{pmatrix}, \tag{S52}$$

which can be interpreted as a Krylov sequence for the Koopman operator $\mathscr{U}_\ell = \otimes_1^\ell \mathscr{U}$, $\mathscr{U}_\ell \mathbf{h} = \mathbf{h} \circ \mathbf{T}$. The techniques from §S1.3 now apply in this new setting simply by setting $\mathbf{h}$ instead of $\mathbf{f}$, and ℓ instead of d. The matrix $\mathbb{H}$ plays the role of the snapshot matrix[3] $\mathbf{S}$, and we have $\mathbf{X} = \mathbb{H}(:, 1 : m_H)$, $\mathbf{Y} = \mathbb{H}(:, 2 : m_H + 1)$. We will attempt predicting the $\mathbf{h}_k$'s, and from the obtained results extract the predictions of the original observable $\mathbf{f}$. The starting points are the DMD of $\mathbb{H}$ (H-DMD), and the corresponding KMD. Since the KMD changes with the sliding active data window $\mathscr{W}(p, \mathsf{w})$, we use the term active KMD (AKMD) when we refer to the computation used for prediction.

For numerical implementations of the DMD see[47–52].

[3]Note that this is different from a system identification technique based on the SVD decomposition of $\mathbb{H}$.

S1.4.3 Prediction - the basic idea and its limitations

Suppose that the DMD algorithm, applied to (S50), has extracted $r = m_H$ Ritz pairs, and that the Ritz vector span the range of $\mathbb{H}$. Then we can determine the coefficients α_j such that

$$\mathbf{h}_k = \mathbb{A}^{k-1}\mathbf{h}_1 = \sum_{j=1}^{m_H} \lambda_j^{k-1}\alpha_j \mathbf{v}_j + \delta_{k,m_H+1}\widehat{\mathbf{r}}_{m_H+1}, \;\; k = 1,\dots,m_H+1, \tag{S53}$$

where δ_{k,m_H+1} is the Kronecker delta symbol, and $\widehat{\mathbf{r}}_{m_H+1}$ is the residual of the orthogonal projection of $\mathbf{h}_{m_H+1}$ onto the range of $\mathbf{X}$. This means that the decomposition of the snapshots in terms of the modes is exact, except for the last one, which may not belong to the range of $\mathbf{X}$, and the residual $\widehat{\mathbf{r}}_{m_H+1}$ represents its decomposition error. For details we refer to [13, §2.4, §3.2]. If we want to extend the above relation beyond the index $k = m_H + 1$ (i.e. to extrapolate into the future the evolution of the sequence $\mathbf{h}_k$), we can apply the appropriate power of $\mathbb{A}$ and use the approximation $\mathbb{A}\mathbf{v}_j = \lambda_j \mathbf{v}_j + \mathbf{r}_j \approx \lambda_j \mathbf{v}_j$. This is a fairly simple operation - it amounts to increasing the power of the λ_j's. Of course, the residuals will accumulate with each such iteration, e.g.

$$\mathbb{A}\mathbf{h}_{m_H+1} = \sum_{j=1}^{m_H} \lambda_j^{m_H+1}\alpha_j \mathbf{v}_j + \sum_{j=1}^{m_H} \lambda_j^{m_H}\alpha_j \mathbf{r}_j + \mathbb{A}\widehat{\mathbf{r}}_{m_H+1}, \tag{S54}$$

$$\mathbb{A}^2\mathbf{h}_{m_H+1} = \sum_{j=1}^{m_H} \lambda_j^{m_H+2}\alpha_j \mathbf{v}_j + \sum_{j=1}^{m_H} \lambda_j^{m_H+1}\alpha_j \mathbf{r}_j + \sum_{j=1}^{m_H} \lambda_j^{m_H}\alpha_j \mathbb{A}\mathbf{r}_j + \mathbb{A}^2\widehat{\mathbf{r}}_{m_H+1}, \;\; \mathbb{A}^3\mathbf{h}_{m_H+1} = \dots \tag{S55}$$

So, using the first sums above (and ignoring the residual terms) to predict future of the $\mathbf{h}_k$'s has limited range, except in the case of small $\widehat{\mathbf{r}}_{m_H+1}$ and small residuals $\mathbf{r}_j$, which are not too much amplified under the action of the powers of $\mathbb{A}$. Hence, it is desirable to have a KMD that uses only the selected modes corresponding to the Ritz pairs with small residuals, and that we can have an accurate decomposition of the type (S45), using the selected modes. This selection is possible in data driven scenarios using the methods from[44] and[12], outlined in §S1.3.6. The desire to have a high fidelity representation of the data snapshots with as few as possible modes $\mathbf{v}_j$ is motivated by revealing latent coherent structures of the e.g. flow field; small residuals allow for extrapolation of the dynamics forward in time.

If our goal is solely the prediction, the weight factors w_i in (S46) can be tuned to favor most recent snapshots, and the weighting matrix Ω can emphasize particular block rows in the $\mathbf{h}_k$'s; see §S1.3.7 and [14, §3]. In particular, with a suitable choice of $\mathbf{W}$ and Ω, we can focus the reconstruction of the $\mathbf{h}_k$'s to the present snapshot $\mathbf{f}_{n+n_H+m_H-1} = \mathbf{f}_{t_{p-1}}$.

Since the $\mathbf{f}_k$'s, starting from the past time stamp index b and ending at the present index $p-1 = b+n_H+m_H-1$, are in the last block row of $\mathbb{H}$ (see (S50)), the corresponding formulas are obtained by taking the last d components of the Ritz vectors $\mathbf{v}_j$. To that end, define $\widehat{\mathbf{v}}_j = \mathbf{v}_j((n_H-1)d+1 : n_H d)$ as the trailing d components of $\mathbf{v}_j$. Hence, from the AKMD of the lifted observables, we read off approximate decomposition of the snapshots $\mathbf{f}_k$ as

$$\widetilde{\mathbf{f}}_k = \sum_{j=1}^{r} \widehat{\mathbf{v}}_j \alpha_j \lambda_j^{k-1}. \tag{S56}$$

For $k = b+n_H,...,b+n_H+m_H-1$, (S56) is a reconstruction of the acquired data, while for $k = p, p+1,\dots,p+\tau(p)$, (S56) is an extrapolation of the AKMD, and it gives us predictions for future data snapshots. We say that $\widetilde{\mathbf{f}}_{p+\tau}$, $\tau > 0$ is the prediction of the observable at the lead time τ.

If the number of rows n_H of Hankel matrix is smaller than number of columns m_H $(n_H < m_H)$ then the KMD gives some sort of regression function for the data in the reconstruction window $b+n_H,\dots,b+n_H+m_H-1$. The reason why the first part of active window is not declared as reconstruction window is that when the KMD of the form (S56) is used, the Koopman eigenfunction values are determined such that for $t = 0$ the sum of the right hand side is equal to the first snapshot. When applied to Hankel matrix this means that the data in the beginning of the active window $b,\dots,b+n_H-1$, which form the first column of Hankel matrix are reconstructed with high accuracy. On the other hand, if $n_H \geq m_H$ and if the Hankel matrix has full column rank, the data in the whole active window are reconstructed with high accuracy.

S1.5 A worked example

We now illustrate the key elements of the procedure outlined in §S1.4.2, §S1.4.3 using a worked example. The problem under study is the spread of the coronavirus disease (COVID-19). The data consists of reported cummulative daily cases in some European countries. It should be stressed that the algorithm uses only the raw data – no other information on the nature of the data or on modelling parameters is assumed. Further, the data itself is clearly not ideal, as it depends on the reliability of the tests, testing policies in different countries (triage, number of tests, reporting intervals, reduced testing during the weekends), contact tracing strategies, the number of asymptomatic transmissions etc. Moreover, using the data from different countries in

the same vector observable poses an additional difficulty for a data driven revealing of the dynamics, because the countries independently and in an uncoordinated manner impose different restrictions, thus changing the dynamics.

For an analysis of a particular country, it is better to define the observables as the reported cases on local level, e.g. provinces, counties, cities with similar conditions. Clearly, the dynamics of the spread of the infection depends on the population density as well. This is best seen e.g. by comparing the heat map of the reported cases in the USA with the image of the USA from space at night. In the numerical examples in this section, we purposely use data from different countries to make the prediction task more challenging, which makes it an excellent stress test example.

Our goal with this example is twofold. First, we show the potentials and the limits of the proposed prediction algorithm. Secondly, we discuss technical details of the computational scheme.

We use the following datasets:

DS1 The numbers of reported COVID-19 cases in Germany, France and the United Kingdom in the period February 29 to November 18. The ordered triple of reported cases is an observable.

DS2 The dataset **DS1** augmented by the numbers of reported cases in Denmark, Czechia, Slovenia, Austria and Slovakia.

DS3 The numbers of reported COVID-19 cases in a selected European country in the period February 29 to November 18, augmented with two sequences of filtered data.

The test of the prediction algorithm runs on the lifted data (265 observables from $\mathbb{R}^d$: $d = 3$ for **DS1** and **DS3**; $d = 8$ for **DS2**) i.e. on the columns of the $94d \times 172$ Hankel matrix $\mathbb{H} = \begin{pmatrix} \mathbf{h}_1 & \mathbf{h}_2 & \dots & \mathbf{h}_{172} \end{pmatrix}$ (see (S50)) with the block partition 94×172, each block being $d \times 1$. The matrix $\mathbb{H}$ is used as a historical record, encoding the period February 29 – November 18, and we run the prediction algorithm starting from some past index and test its accuracy by comparison with the historical data. We use simple increasing window starting at the index $b = 1$ and ending at $p - 1$, where we choose different values of p. Then we predict the next $\tau + 1$ values from the moment p on.

In the first experiment, we use **DS1** and attempt prediction for 35 days ahead. We take the first 40 columns of $\mathbb{H}$ as available data and set $\mathbf{X} = \mathbb{H}(1:282, 1:39)$, $\mathbf{Y} = \mathbb{H}(1:282, 2:40)$. (This corresponds to the period February 29 – July 10, and the prediction for 35 days ahead starts July 11.) The prediction relative error is shown on the left panel in Figure S1. The right panel shows the Koopman Ritz values computed in the algorithm; note that the algorithm has revealed the eigenvalue 1, and that all other Ritz values are inside the unit circle. The quite satisfactory prediction skill (recall, no information whatsoever on the nature of the data is used) and well behaved Ritz values are related to the nature of the dynamics of the infection during the summer.

In this example, it is instructive to check the Government Response Stringency Index (GRSI)[534] for the entire time interval involved in the computation. The three indexes behave differently: it can be noticed that France had sharper changes than Germany and the United Kingdom (e.g. around June 20), and sometimes similar increase of stringency but a week earlier than the other two countries (e.g. beginning to mid March). On the other hand, the GRSI for Germany and the United Kingdom were not that much different throughout the observed period; see the left panel in Figure S2. It should be noted, however, that the GRSI does not measure the quality of the implementation of the imposed restriction and that for a particular country it does not necessarily indicate the trends in the dynamics of the disease spreading.

[4] For an interactive exploration of the GSRI see https://ourworldindata.org.

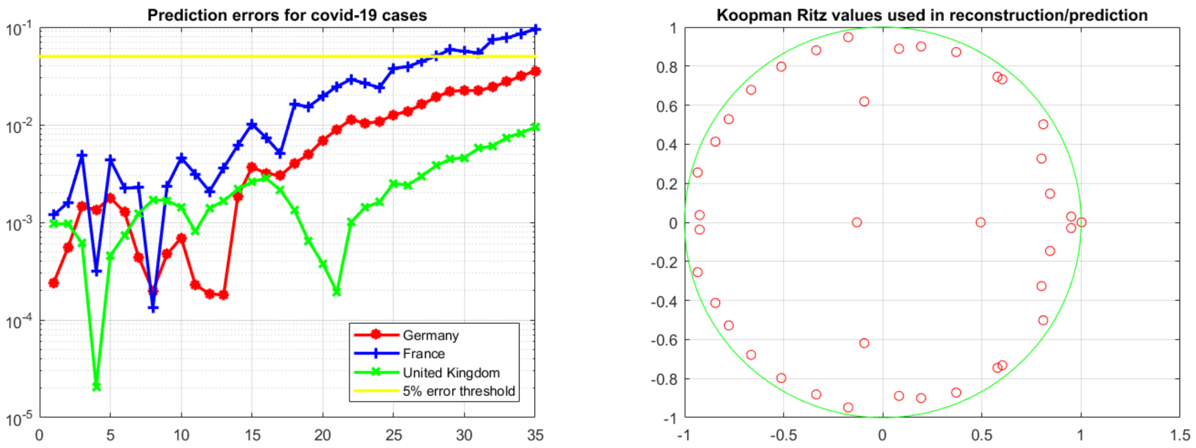

Figure S1. *Left panel*: Relative prediction error for Germany, France and the United Kingdom for a 35 days prediction starting after the data window $\mathbf{h}_{1:40}$. (In terms of the original data, prediction starts on July 11.) *Right panel*: The Koopman Ritz values used in the KMD.

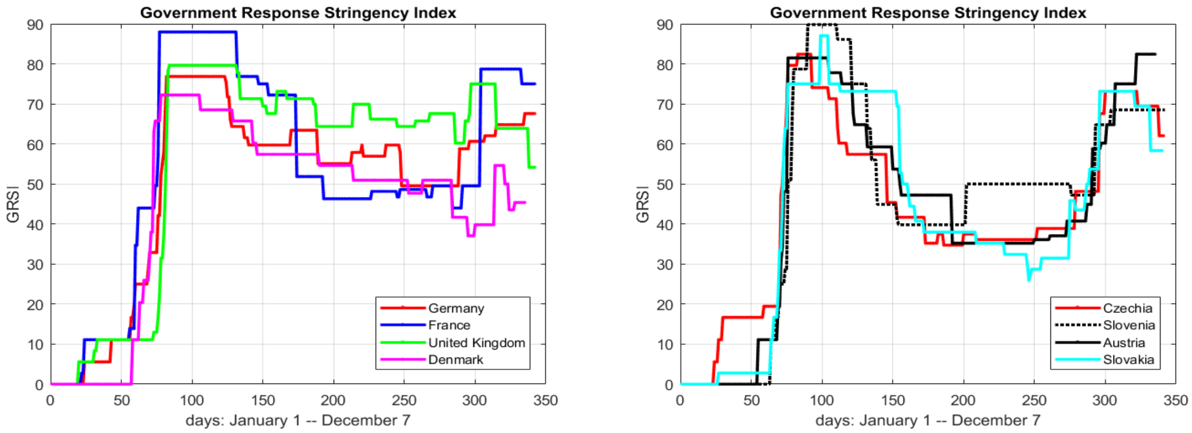

Figure S2. Government Response Stringency Index measures response indicators (OxCGRT indicators) such as school closing, workplace closings, cancelling public events, restrictions on gathering size, closing public transport, stay at home requirement, restriction on internal movement and international travel. The GRSI data for Germany, France, United Kingdom, Denmark, Czechia, Slovenia, Austria and Slovakia are taken from[53]. For more details see[54].

Now, in the same interval, we add new observables by including the data from five more countries: Danemark, Czechia, Slovenia, Austria and Slovakia. Hence, the matrix $\mathbb{H}$ is 752×172. The prediction errors for a 28 days prediction are given in the left panel in Figure S3. Remarkably, the computed Koopman Ritz values nearly match the one computed in the first test with only three countries, see the right panel in Figure S3. Note that even with the differences shown in Figure S2, the main trend of the implementation of the measures is similar. This might help explain the robustness of the spectrum indicated in Figure S3, where such differences do not seem to lead to drastic change in the spectral behavior. We believe this indicates the robustness of our methodology.

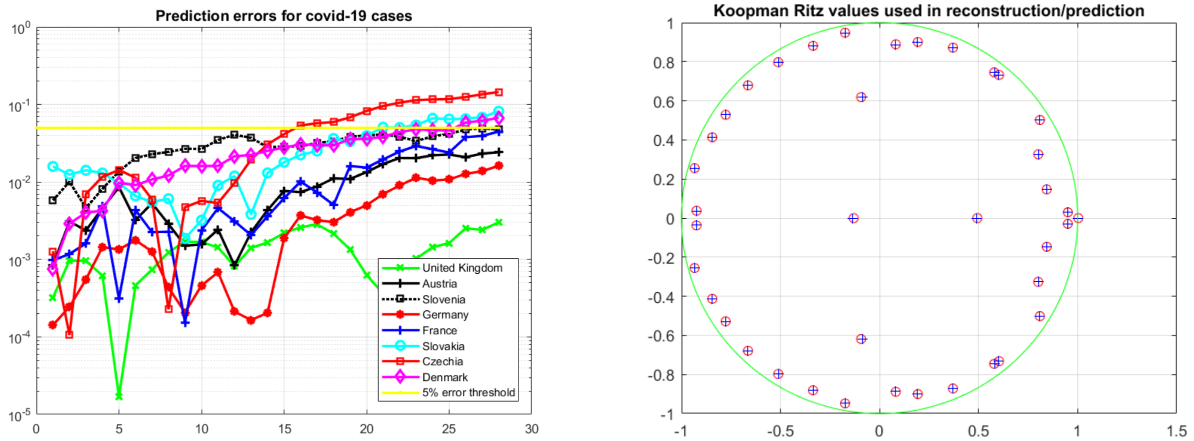

Figure S3. *Left panel*: Relative prediction error for eight European countries for a 28 days prediction starting after the data window $\mathbf{h}_{1:40}$. (In terms of the original data, prediction starts on July 11.) *Right panel*: The Koopman Ritz values used in the KMD, denoted as blue pluses (+). The red circles (∘) denote the Ritz values computed using only three countries as shown in Figure S1.

We proceed with the numerical experiment using the dataset **DS1**. We further expand the learning window and then consider three consecutive steps with $\mathbf{h}_{1:105}$, $\mathbf{h}_{1:106}$, $\mathbf{h}_{1:107}$. The relative errors for 35 days prediction are shown in the first row of Figure S4. In the context of policy changes that affected the dynamics of the infection spreading, and the fact that the algorithm is purely data driven, the results can be considered satisfactory: in the first graph, the error is below five percent for 16 days and below ten percent for three weeks for all three countries (first graph), below six percent for almost entire 35 days period (second graph), below five percent for more than three weeks and below ten percent for 30 days (third graph).

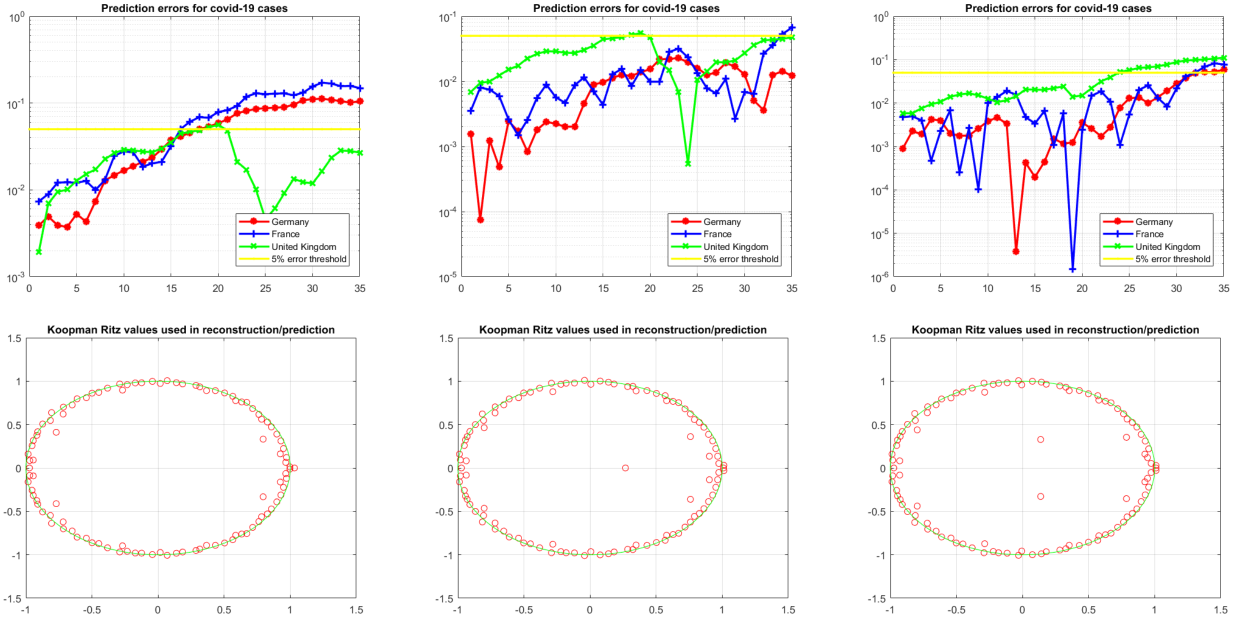

Figure S4. *First row*: Prediction error for Germany, France and the United Kingdom for a 35 days prediction starting from the data windows $\mathbf{h}_{1:105}$ (prediction for September 14 – October 18), $\mathbf{h}_{1:106}$ (prediction for September 15 – October 19), $\mathbf{h}_{1:107}$ (prediction for September 16 – October 20), respectively. *Second row*: The corresponding Koopman Ritz values used in the KMD.

In the next test, we use the data windows $\mathbf{h}_{1:132}, \ldots, \mathbf{h}_{1:140}$ for 28 days predictions for the time intervals October 11 - November 7, October 12 - November, ..., October 19 - November 15. The results are shown in Figure S5.

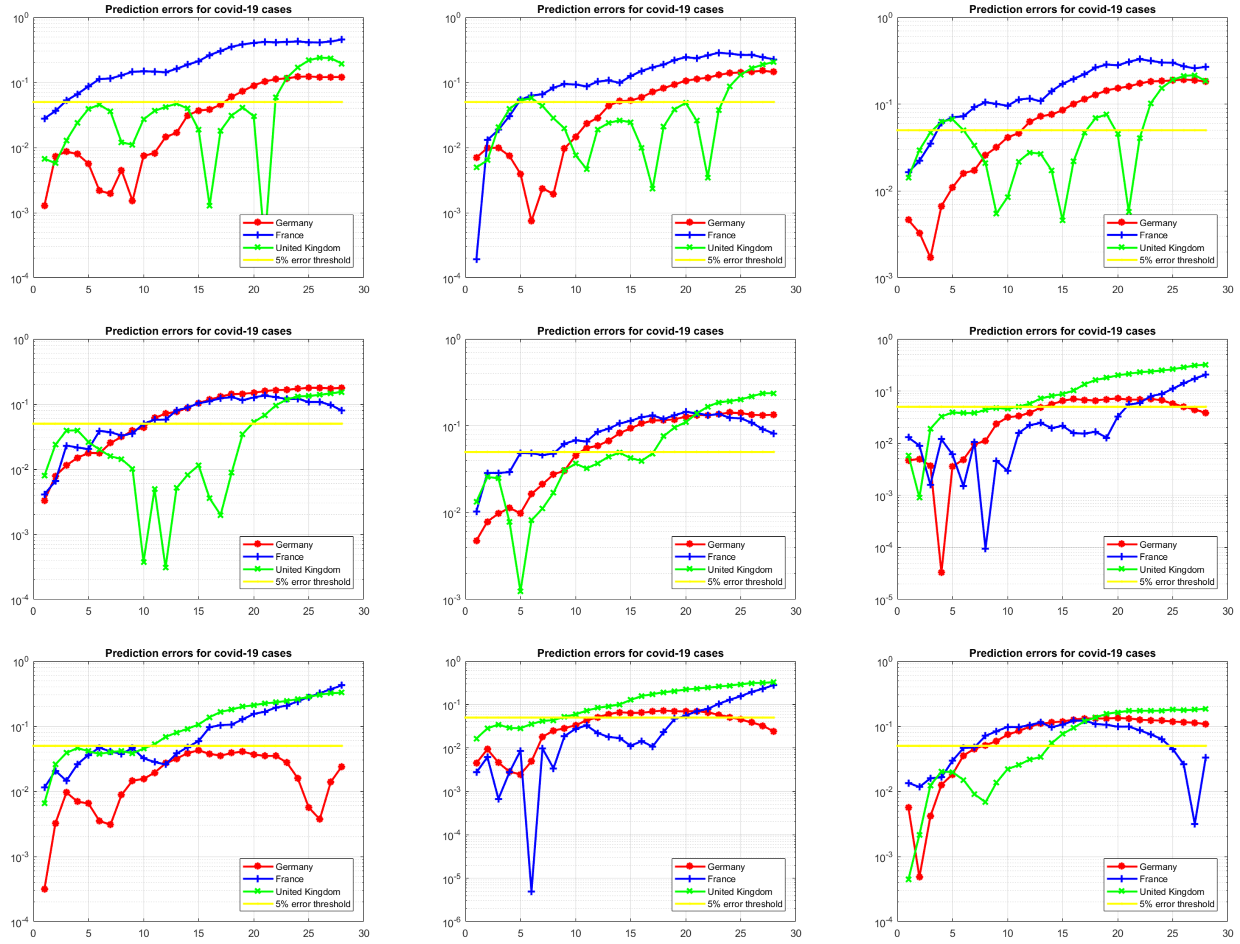

Figure S5. Prediction error for Germany, France and the United Kingdom for a 28 days prediction, based on the windows $\mathbf{h}_{1:132},\ldots,\mathbf{h}_{1:140}$, respectively. The prediction intervals are, respectively, October 11 - November 7, October 12 - November, ..., October 19 - November 15.

Now, we go to the datased **DS3**. The focus is on some computational details related to the two main ingredients – the DMD and the KMD. The datased **DS3** is constructed by a single and a double application of the Savitzky-Golay filter to the Germany data, so that $d = 3$. (The filter uses cubic polynomial and data window of width 5. On the left boundary, we add zero values, and on the right boundary we leave the original data. The filtered data differ from the original at most five to ten percent relative error in the first 30 days and at most $O(10^{-3})$ afterwards.) The purpose of the test is to create a situation that one could encounter when deploying the Koopman/DMD framework for data driven prediction or for a discovery and analysis of latent coherent structures.

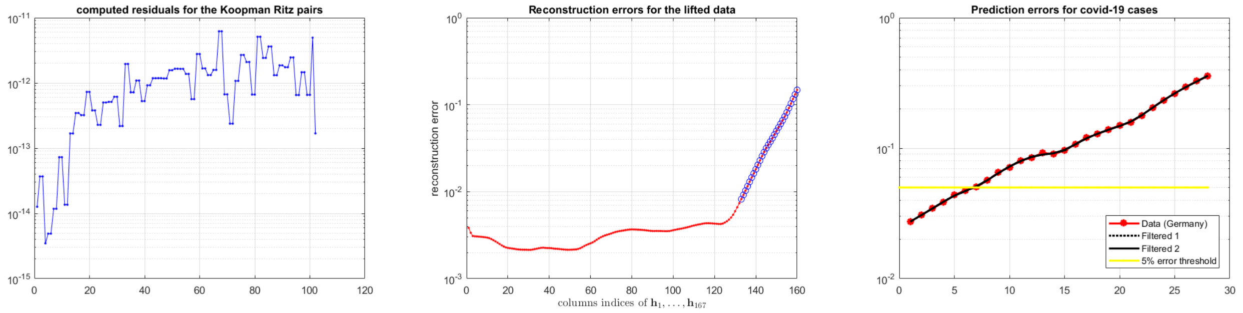

Figure S6. Prediction experiment with **DS3** with data from Germany. *Left panel*: the computed residuals for the computed 102 Koopman Ritz pairs (extracted from a subspace spanned by 132 snapshots $\mathbf{h}_{1:132}$). Note that all residuals are small. The corresponding Ritz values are shown in the first panel in Figure S7. *Middle panel*: KMD reconstruction error for $\mathbf{h}_{1:132}$ and the error in the predicted values $\mathbf{h}_{133:160}$ (encircled with $\circ$). The reconstruction is based on the coefficients $(\alpha_j)_{j=1}^r = \arg\min_{\alpha_j} \sum_k \|\mathbf{h}_k - \sum_{j=1}^r \lambda_j^k \alpha_j \mathbf{v}_j\|_2^2$. *Right panel*: Prediction errors for the period October 11 – November 7. Compare with the first graph in Figure S5.

We recall that a DMD algorithm uses a rank revealing decomposition with some threshold value and that the number of the computed Ritz pairs may be smaller than the column dimension of the matrix $\mathbf{X}$; see §S1.3.5. Then the reconstruction formula (S44) for the coefficients is not valid, and one has to satisfy (S43) by solving the least squares problem $\sum_k \|\mathbf{h}_k - \sum_{j=1}^r \lambda_j^k \alpha_j \mathbf{v}_j, \|_2^2 \longrightarrow \min_{\alpha_j}$, where the reconstruction error is not necessarily small, and it introduces noise into the extrapolation process outlined in §S1.4.3. (Recall, if we have full set of modes, then the reconstruction is perfect and the only error is from the finite precision arithmetic.) The prediction skill based on this KMD is shown in Figure S6. Now we change the reconstruction strategy and state the problem as the weighted least squares problem (S46) with the weights that favour the four most recent snapshots with weights set to one, and with the weights of all other snapshots set to the machine round-off unit $\varepsilon \approx 2.2 \cdot 10^{-16}$.

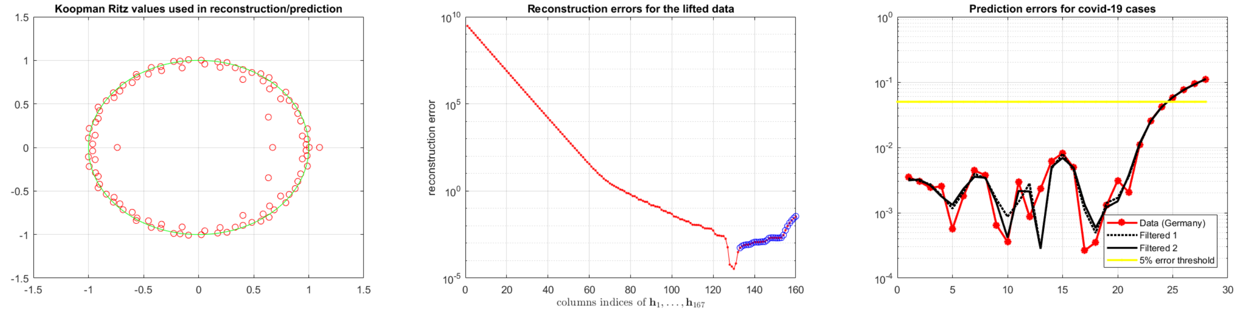

Figure S7. Prediction experiment with **DS3** with data from Germany. *Left panel*: the computed 102 Koopman Ritz values (extracted from a subspace spanned by 132 snapshots $\mathbf{h}_{1:132}$). The corresponding residuals are shown in the first panel in Figure S6. *Middle panel*: KMD reconstruction error for $\mathbf{h}_{1:132}$ and the error in the predicted values $\mathbf{h}_{133:160}$ (encircled with $\circ$). The reconstruction is based on the coefficients $(\alpha_j)_{j=1}^r = \arg\min_{\alpha_j} \sum_k w_k^2 \|\mathbf{h}_k - \sum_{j=1}^r \lambda_j^k \alpha_j \mathbf{v}_j\|_2^2$. *Right panel*: Prediction errors for the period October 11 – November 7. Compare with the first graph in Figure S5, and with the third graph in Figure S6.

The effect of weighting is best seen by comparing the middle graphs in Figures S6 and S7. In the case of weighting, the reconstruction of almost all leading snapshots is bad, but the ones more important for the prediction task have much smaller error.

S1.6 Comments on SIR type models

The key coefficient in SIR-type models, the so-called reproduction number R_0 can be estimated using Koopman operator techniques. Namely, the classic SIR model reads

$$\begin{aligned} \dot{s} &= -\beta s i \\ \dot{i} &= \beta s i - \nu i \\ \dot{r} &= \nu i \end{aligned} \tag{S57}$$

Under condition $s = 1$ (infinite reservoar of susceptibles), the exponential growth happens when $\beta - \nu > 0$, i.e. $\beta/\nu > 1$. The reproduction number is defined by $R_0 = \beta/\nu$. Thus, R_0 is related to the coefficient of exponential growth. Since we know that eigenvalues of the linearized system are eigenvalues of the Koopman operator, the largest real Koopman eigenvalue is related to R_0. Another number commonly estimated for use in tracking of epidemics is the instantaneous reproduction number defined by[55]

$$R_t^{inst} = \beta s D,$$

where D is the duration of the infectiousness. For small ν, and constant $s = 1$, we have

$$R_t^{inst} \approx \beta D, \tag{S58}$$

providing another connection between the SIR models, Koopman operator spectrum and Reproduction numbers.

S1.7 The framework for prediction and Black Swan event detection

In GKP algorithm, the spectral information is extracted from a sequence of active windows – for each window, the snapshots are arranged in a Hankel matrix whose columns define a new set of snapshots and approximate eigenvalues and eigenvectors are computed using Algorithm S2. In the case when the dynamics of the system is not coupled with some other dynamical system, we expect that, in the absence of unexpected disturbances, the AKMD will capture at least the basic trends of the dynamics. In particular, the spectral radius of the active window (the maximal absolute value of the selected Ritz values) should not change too much. Further, the DMD algorithm should compute Ritz pairs with reasonably small residuals. This is plausible, because the sequence (S14) can be, at any moment, interpreted as an excerpt from a power method generated sequence, and the power method in the limit reveals the absolutely dominant eigenvalues.

However, if the dynamical system data are hit by disturbance, this could be recognized e.g. by detecting the active windows whose spectral radii change significantly, or by the absence of Ritz pairs with small residuals (see Figures S8b, S8c, S8d). This enables us to pinpoint the discrete time moments/subintervals at which disturbances interfere with the original dynamics. For the chosen reference interval $\mathscr{I}$, if $\max_j |\lambda_j| \notin \mathscr{I}$ or if there are no Ritz pairs with reasonably small residuals, we flag the observed active window as the window which possibly contains a Black Swan event. By sliding the active windows along the computational domain, using the flagged windows, we determine the time sub-intervals containing disturbances whose dynamics is not well captured by the corresponding AKMD models.

The reference interval $\mathscr{I}$ can be determined (and dynamically adjusted) e.g. by first computing the Ritz values for many active windows, and then by trial and error, including a statistical reasoning and information theoretic techniques (see e.g.[56]) learn to differentiate between the acceptable interval $\mathscr{I}$ for spectral radii and the values that are considered outliers. This is best done on a case-by-case basis.

This scheme can be implemented with different sizes of the Hankel matrices (see §S1.4.2) and with different sizes of active windows and then the Black Swan event intervals can be determined by taking into account all determined intervals.

S1.7.1 The retouching trick to process Black Swan events

If the Black Swan event data are included in the training set, the dynamics of the original system (decoupled from this disturbance) cannot be revealed, which means that the prediction of the dynamics after the Black Swan event will be damaged, if not impossible. However, instead of using the original data we can replace them with the data obtained by the prediction based on the information from the previous active windows, preceding flagged intervals. This is illustrated in (S59): the perturbed value $(\mathscr{U}^k\mathbf{f})(\mathbf{z}_0) + \varepsilon_k$ is replaced with $\widehat{(\mathscr{U}^k\mathbf{f})(\mathbf{z}_0)}$, which is a predicted value based on the previous undisturbed data. The same can be done for the remaining data in the flagged window.

undisturbed	$\dots$	$(\mathscr{U}^{k-1}\mathbf{f})(\mathbf{z}_0)$	$(\mathscr{U}^k\mathbf{f})(\mathbf{z}_0)$	$(\mathscr{U}^{k+1}\mathbf{f})(\mathbf{z}_0)$	$\dots$
disturbance at k, $k+1$	$\dots$	$(\mathscr{U}^{k-1}\mathbf{f})(\mathbf{z}_0)$	$(\mathscr{U}^k\mathbf{f})(\mathbf{z}_0) + \varepsilon_k$	$(\mathscr{U}^{k+1}\mathbf{f})(\mathbf{z}_0) + \mathscr{U}\varepsilon_k + \varepsilon_{k+1}$	$\dots$
use prediction at k, $k+1$	$\dots$	$(\mathscr{U}^{k-1}\mathbf{f})(\mathbf{z}_0)$	$\widehat{(\mathscr{U}^k\mathbf{f})(\mathbf{z}_0)}$	$\widehat{(\mathscr{U}^{k+1}\mathbf{f})(\mathbf{z}_0)}$	$\dots$

(S59)

The prediction after the Black Swan event then becomes more stable and in most cases quite successfully predicts data after the Black Swan event; see §2.2 and Fig. 2 in the main text.

There are many variations of this scheme. For instance, it could happen that the Black Swan interval detected in the algorithm is too long and possibly unrealistic. Therefore we limit the length of the interval on which the data are replaced in order to prevent the algorithm from changing the dynamics too much. Then we apply the algorithm again and detect if the replacements result with decreasing of maximum of the absolute value of the eigenvalues over the active windows. The whole process can be repeated more times to remove eventual Black Swan events that are not taken into account in the previous steps. Finally, the retouched data, cleaned from the Black Swan event disturbances, are used for the prediction.

Algorithm S3 (Global Koopman Prediction (GKP) with Black Swan event detection and switching to local prediction)

Require: • Data snapshots $\mathbf{f}_0, \mathbf{f}_1, ..., \mathbf{f}_{end}$; • the size of the training data window $\mathsf{w} = n_H + m_H$; • the dimensions n_H, m_H ($n_H > m_H$) of the Hankel matrices; • the threshold $\eta > 0$ for the maximal acceptable value of the residual of Ritz vectors; • the sliding step Δp; • the maximal number N_{rep} of iterative retouching of the perturbed data; • the maximal time length L_{BS} for one step replacement of the Black Swan event data with the predicted values; • the reference interval $\mathscr{I}$ for spectral radius used for the detection of Black Swan event moments; • lead times τ_g and τ_l for the global and local prediction.

Ensure: Predicted system observables $\widetilde{\mathbf{f}}_{n_H+m_H}, \widetilde{\mathbf{f}}_{n_H+m_H+1}, \ldots, \widetilde{\mathbf{f}}_{\mathsf{w}+n_{dmd}\cdot\Delta p+\tau_g}$ (or $\widetilde{\mathbf{f}}_{\mathsf{w}+n_{dmd}\cdot\Delta p+\tau_l}$)

```
j_rep = 0; n_dmd = ⌊(end−w)/Δp⌋; BS_event = False, n_BS = 0
while (j_rep < N_rep) and (j_rep = 0 or n_BS ≠ 0) do
    n_BS = 0
    for p = w, w+Δp, ..., w+n_dmd·Δp do
        For the active window W(p,w) apply KMD algorithm to obtain AKMD using n_H × m_H Hankel matrices.
        If there are no Ritz values for which the associated residual is smaller than η, set max_j |λ_j| = ∞.
        if max_j |λ_j| ∉ I then
            if BS_event = False then
                Set t_BSbegin = t_max(0,p−Δp); BS_event = True; n_BS = n_BS + 1        ▷ New disturbance appears.
            end if
            Flag the time interval [t_{p−Δp}, t_p] as a Black Swan event interval;
            In the interval [t_{p−Δp}, t_p] use Local Koopman Prediction with lead time τ_l (Algorithm S4)
            ▷ Remark: Local Koopman Prediction algorithm (Algorithm S4) can be applied on the whole domain and then
              associated local prediction is used on the detected critical intervals.
            Store the data from last active window not including Black Swan event for retouching the data in [t_{p−Δp}, t_p]
              using the prediction obtained by global AKMD .
        else
            if BS_event = True then
                Set t_BSend = t_{p−Δp}; BS_event = False        ▷ End of the Black Swan event.
                Replace the original data in the Black Swan event interval [t_BSbegin, min(t_BSbegin + L_BS, t_BSend] with the stored
                  retouched data.
            else
                Using the AKMD associated with W(p,w) and (S56), extrapolate to obtain the predictions f̃_{p+τ}, τ ≤ τ_g.
            end if
        end if
    end for
    j_rep = j_rep + 1
end while
```

S1.7.2 Local Koopman prediction

In some cases, the global prediction algorithm is not feasible. For instance, when we just start collecting the data, we have not enough information for a GKP analysis. Or, in the situation when GKP recognizes the beginning of a Black Swan event, as discussed at the beginning of §S1.7. Then, the available data cannot be used for prediction, because the dynamical system has changed. The new model must be built from scratch, as if we just started getting new data. The best we can do is to create a new local algorithm that needs less data, but also with a much shorter reach into the future.

In the Local Koopman Prediction (LKP) algorithm we change the size of the active window depending on the success of the previous prediction. The idea is to assimilate as much acquired data as possible, so we set Hankel matrix dimension variable with prediction moment, i.e. $n_H = n_H(p)$ and $m_H = m_H(p)$. We also choose the minimal Hankel matrix dimension

$$(n_{H,min} \cdot d) \times (m_{H,min} + 1), \tag{S60}$$

and we start predictions with such minimal Hankel matrix i.e. for first prediction $p = p_0$ we set

$$n_H(p_0) = n_{H,min}, \;\; m_H(p_0) = m_{H,min}. \tag{S61}$$

When data $\mathbf{f}_p$ at prediction time t_p becomes available, we can compute the error of the prediction $\widetilde{\mathbf{f}}_p$, using suitable norm, as

$$\varepsilon_p = \|\widetilde{\mathbf{f}}_p - \mathbf{f}_p\| / \|\mathbf{f}_p\|. \tag{S62}$$

At other prediction moments, if the prediction error (S62) is smaller than the referent error ε_{ref} we assimilate the newly acquired data into the active window by increasing the Hankel matrix size

$$n_H(p) = n_H(p-1) + 1, \text{ or } m_H(p) = m_H(p-1) + 1. \tag{S63}$$

Otherwise, i.e. if the prediction error (S62) is larger then the referent one, we reset the Hankel matrix dimension to the minimal one: $n_H(p) = n_{H,min}$, $m_H(p) = m_{H,min}$.

In both cases we recompute the Hankel matrix and the AKMD for each new prediction.

Algorithm S4 (Local Koopman Prediction (LKP) with resizing Hankel matrix)

Require: • Data snapshots $\mathbf{f}_0, \mathbf{f}_1, \ldots, \mathbf{f}_{end}$; • indices of time moments for the begin and the end of the local prediction k_0, k_f (optionally) • minimal Hankel matrix dimension $n_{H,min}$, $m_{H,min}$; • error threshold ε_{ref}; • lead time τ_l.

Ensure: Predicted system observables $\widetilde{\mathbf{f}}_{n_{H,min}+m_{H,min}}, \widetilde{\mathbf{f}}_{n_{H,min}+m_{H,min}+1}, \ldots, \widetilde{\mathbf{f}}_{end+\tau_l}$ (or $\widetilde{\mathbf{f}}_{k_0}, \ldots, \widetilde{\mathbf{f}}_{k_f+\tau_l}$)

```
if k_0 and k_f not defined then
    k_0 = n_{H,min} + m_{H,min}, k_f = end
end if
for p = k_0, k_0 + 1, ..., k_f let t_{p-1} be the time of the last known data do
    if the error ε_p (S62) is larger than referent error ε_ref then
        Resize the Hankel matrix to the minimal size (S61).
    else
        Increase the size of the Hankel matrix using (S63).
    end if
    Form the Hankel matrix (S50) and compute the AKMD.
    Using the AKMD, extrapolate to obtain the prediction (S56).
end for
```

S2 Supplementary material – Discussion.

In this supplementary material we validate our approach by three case studies. In §S2.1, we use the Lorenz system to illustrate the main idea of monitoring the Koopman Ritz values and the prediction skills of the proposed method. The model free aspect of the proposed method is further illustrated in applications in two entirely different settings: physiological processes (resonant breathing) in Supplementary section S2.2 and geomagnetic substorms (prediction of the AL index) in Supplementary section S2.3. Finally, in Supplementary section S2.4 we provide additional numerical results related to the influenza epidemics studied in section 2.2 of the paper.

S2.1 Case study: Lorenz system

The critical underlying concept in chaotic dynamics is that of sensitivity to initial conditions and the associated positivity of Lyapunov exponents that measure long term exponential deviation of nearby trajectories[57]. Namely, the long term exponential divergence of nearby trajectories leads to unpredictability due to the finite nature of (any) prediction algorithm. Even the implementation of exact equations of a dynamical system on any computing device leads to finite precision calculations and therefore ultimate exponential divergence of prediction from true trajectory. However, this neglects the finer aspects of chaotic dynamics that are exhibited in the most paradigmatic of chaotic systems – the Lorenz dynamical systems, modeled by Lorenz equations

$$\dot{\mathbf{x}} = \begin{pmatrix} \dot{x} \\ \dot{y} \\ \dot{z} \end{pmatrix} = \begin{pmatrix} \sigma(y-x) \\ x(\rho - z) - y \\ xy - \beta z \end{pmatrix} \tag{S64}$$

with $\sigma = 10$, $\beta = 8/3$, and $\rho = 28$ for which the system exhibits chaotic behavior. For understanding of the prediction capability for the Lorenz system, more important than the long term exponential divergence of trajectories is the short term divergence typically induced by switching between the two *wings* of the butterfly (see Figure S8a).

At the core of our approach is the observation that, while inside one of the butterfly wings, the system behaves in a predictable manner. The exponential divergence is ultimately due to switching between the two butterfly wings. The first time such a switch happens, the situation resembles a Black Swan event[15] (although there is an ontological difference highlighted in the main text): the trajectory suddenly wonders off to a different part of the state space and starts exploring there, until

another switch happens taking it back to the known part of the state space. This fits our paradigm of splitting the state space into domains over which prediction is possible and monitoring for the switch between such domains.

The current theory is thus an extension of the ideas in[20], where deterministic components of stochastic dynamical systems were extracted using Koopman operator methods, and[58] where it was shown that Lorenz system can be described well by a set of linear evolution equations driven by stochastic term that induces switching. In both of these, the detection of the switching moment and the precise interaction of local and global behavior on subdomains of state space was not accounted for; we address it here.

We use (S64) to test the prediction potential of the KMD. We have generated data using numerical simulation (ODE solver) of (S64) with the time resolution $\delta t = 0.01s$, thus obtaining a discrete dynamical system. For a present moment (index) p, an *active window* of length w is selected as in §S1.4.2, with $b = \mathsf{p} - \mathsf{w}$, and the selected data are lifted in the Hankel structure. The KMD is computed for the corresponding vector valued observables $\mathbf{h}_i$, and used for their prediction as explained in §1 of the paper. For computing the KMD for the global prediction algorithm, the active windows of size $\mathsf{w} = 400$ and 300×100 Hankel matrices are used. By sliding the active windows along the computational domain we get prediction at different times. When

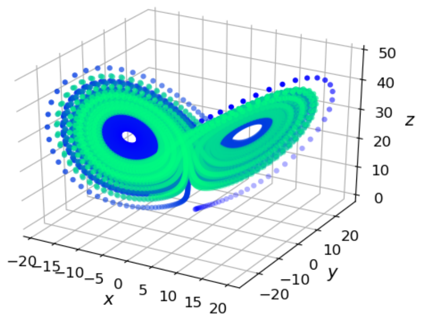

(a) *Lorenz system (S64).*

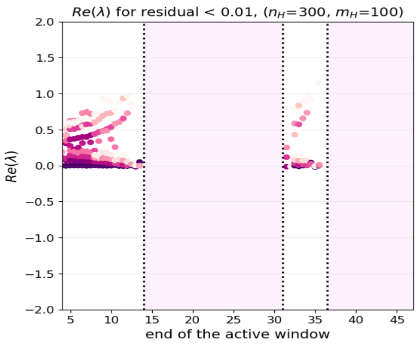

(b) $\Re(\lambda)$

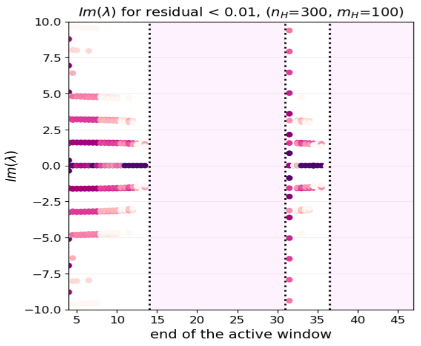

(c) $\Im(\lambda)$

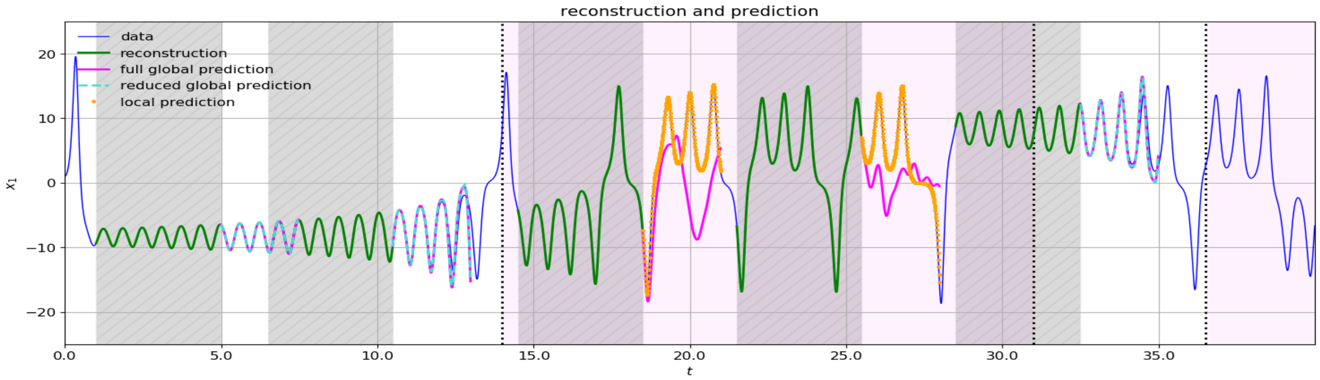

(d) Global and local prediction for 2.5s ahead.

Figure S8. (S8a): the Lorenz system (S64). For each active window, the Ritz pairs with the residual below $\eta_r = 0.01$ are selected (see [12, §3.2]); the real and the imaginary parts of the corresponding Ritz values are shown in (S8b, S8c). The color intensity of the eigenvalues indicates the amplitudes of the corresponding modes. (S8d): KMD reconstruction and prediction of the observable $x_1 \equiv x$ for the Lorenz system (S64). The data are collected in five active windows (time intervals $[1,5]$, $[6.5,10.5]$, $[14.5,18.5]$, $[21.5,25.5]$, $[28.5,32.5]$, marked by shadowed rectangles) and then the dynamics is predicted for the time moments ahead of the active window. Note - by comparing the positions of the intervals with poor prediction with the eigenvalue-free pink rectangles in Figures S8b, S8c - that the failure of the global prediction occurs after the active windows which do not contain Ritz pairs with sufficiently small residuals, as indicated by magenta curves. The local algorithm recovers the prediction capability, using a sequence of shorter moving KMD's and prediction for 10 time steps ahead, as indicated by orange curves.

the actual data and the prediction errors become available, we either continue forecasting with the same KMD, or a switching device invokes the local prediction scheme with 21×11 Hankel matrices if the error is above a preset threshold. The prediction is then with shorter forecast lead time, and predicted data are based on a sequence of local KMD's. The local algorithm keeps increasing the active windows and the lead time, whilst monitoring the error; see the **Methods** section.

In Figure S8, we show the KMD reconstruction and prediction results of the observable $x_1 = x$ of the system (S64) for a

selection of five active windows. While the reconstruction -indicated by green traces - works well (as expected, see e.g.[44],[13],[14]), the prediction capability -indicated by magenta traces - is lost for the third and the fourth active windows. An inspection of the quality of the approximate Ritz pairs computed by the DMD[12] shows that for the time interval containing those two windows none of the computed pairs has the residual below 0.01, i.e. no useful spectral information, which is essential for the KMD, could be extracted from the available data snapshots, see Figures S8b, S8c. As a consequence, the prediction using numerical realization of KMD cannot give satisfactory results.

On the other hand, in that part of the domain where the trajectory behaves chaotically, and the intensive change of the nature of the eigenpairs precludes accurate numerical approximations, local prediction scheme quickly adapts to the new data, forgets the previously acquired information, and delivers better results. See Figure S8d.

The reconstruction with a reduced number of modes (see Figure S8d) uses only the Ritz pairs with small residuals (see [12, §3.2]). The number of modes used for prediction after the first, the second and the fifth active window (gray rectangles) were 10, 18 and 10, respectively. One can observe that the reconstruction and prediction capabilities - shown in dashed green - are comparable with using the full KMD.

S2.1.1 Remark

Regarding the question of detecting the switching moments, Figure S8d provides an insight. If we look at the first pink zone with no “good" eigenvalues (Figures S8b, S8c), we see that it starts close to the switching moment. Also, this zone is quite long because the switching moments in that zone are too close to each other and no learning data window can fit in-between. Only when two switching moments are distant enough, we can find “good" eigenvalues, the learning data window exits the pink zone, and the global prediction recovers. It is remarkable that recent works[59,60] have found spectral objects - pseudoeigenfunctions - that govern quite regular short term dynamics inside the wing of the Lorenz attractor. This dynamical feature - discovered by careful analyses of the continuous Koopman operator spectrum for the Lorenz system - seems to enable the prediction algorithm performance.

S2.1.2 Discussion

Historically, the most discussed way in which a substantial change in dynamics can occur in dynamical systems is due to a change of a value of a bifurcation parameter[61]. The prediction method that we propose is not necessarily related to a change of parameter in the system. Namely, the original description of the black swan event does not relate to a change in parameter, just to travel to another part of the space (here considering the dynamical system to be the ecological system). White swans were known to exist in Europe, but explorers found black swans in Australia. The prediction that an explorer would make when traveling to Australia might have been existence of a white swan. Upon observation, they concluded that black swans exist. The bird had all other properties of the white swan, except for the color. There were no parameter changes, no bifurcation that occurred. Similarly, the prediction of the dynamics while on one wing of the Lorenz butterfly attractor is based on the eigenvalues of the Koopman operator detected while sampling that wing. Once the dynamics "travels" to the other wing, the change in dynamics is recognized (although there are no parameter changes), but as the dynamics continues on the other wing, the same eigenvalues are obtained. The difference is in the resulting local[4] eigenfunctions, (or pseudoeigenfunctions, as in[59,60]), that are related by the symmetry $(x,y) \to (-x,-y)$.

As is well known, chaotic dynamics is an asymptotic property of a dynamical system, and the associated unpredictability is not due to local passage near saddles, but to long term repeat of such events, that ultimately leads to mixing dynamics[62]. Switch in dynamics is here due to internal effects, and thus ontologically different from the Black Swan situation. The switch is due - in the Lorenz case - precisely to the local saddle event, that transitions the dynamics from one wing of the butterfly to the other. We presented a method by which such passage can actually be detected, and accounted for, inside a prediction algorithm.

We note there are methods of prediction of chaotic dynamical systems that can predict the evolution over several Lyapunov times of chaotic systems[63].

Note that our purpose is somewhat different than in[63], We are more interested in detection of failure to predict accurately, then establishing a method for long-term (climate) prediction. In separate work[27] we pursue the question of long-term prediction of the Lorenz model and provide evidence of ability of Koopman based methods to predict over many Lyapunov time-scales.

S2.2 Case study: Resonant breathing

The mathematical model of the human cardiovascular system was developed by Ursino and Magosso in ([64],[65],[66],[67]). This model includes mathematical descriptions of a pulsating heart, as well as the mechanics of blood flow ([68]) and baroreflex activation ([64],[65],[66],[67]). It includes more than 90 parameters and 21 states (pressures, flows, volumes, resistances, and elastances). Twenty-one delay differential equations reflect conservation of mass and balance of forces at arteries and veins, as well as delayed physiological responses to vagal and sympathetic neural activity. This allows for simulation of high-resolution blood pressure and heart period as a function of time. In[69] we modified the Ursino and Magosso model to use experimentally derived

respiration period as a model input. In addition, we set external noise from the Ursino and Magosso model to zero, because of the noise in the respiratory input used in our model.

Data for model validation were provided by 12 men and 12 women who were healthy college students between 21 and 24 year of age. They were participants in an experiment one of the aims of which was to develop a computational physiology approach to model how cardiovascular processes change when the baroreflex mechanism is challenged. This study was approved by the Rutgers University Institutional Review Board for the protection of human subjects involved in research. One of the tasks that the participants completed, was a 5-minutes resonance breathing task (6P) ([70],[71],[72],[73]), during which they breathed at a rate of approximately 6 breaths/min following a visual pacer (Easy Air, Biofeedback Foundation of Europe, Montreal, Canada). The specific details on the participants' selection/exclusion process and experimental procedure can be found in[69].

In[69], to find the optimal set of parameters for each subject, we selected as model output the cost function that takes into account power spectral densities and time averages of several observables, such as heart period. Instead of doing brute-force optimization on the cost function with over 90 parameters in the model, we used the following procedure: 1) an initial sensitivity analysis was performed to select the most important parameters to tune, and 2) optimization of these most important parameters was performed to minimize the cost function. The details of the calibration procedure can be found in[69].

We use the results of the chosen simulation to analyze the global prediction algorithm on it. The numerical solutions were obtained using AIMdyn's GOSUMD software. The used time step for numerical simulations was $\Delta t_0 = 0.003$. The parameters in the simulations, with the exception of the function modeling breathing, were chosen as obtained in[69]. The input breathing function in this simulation was chosen so that in the first part of simulation, the period of input breathing function was constant and equal to 10 seconds. The period of 10 second simulates the rhythm of resonant breathing. In the second half of the simulation, experimentally determined normal breathing function was used as an input.

As already mentioned, in the global prediction algorithm one should provide long enough set of data in order to extract the dynamical system parameters related to the phenomena we want to capture with the algorithm. On the other hand, the time step between the neighboring snapshots should be chosen so that the balance between the numerical complexity and of the length of dynamical phenomena one want to reveal with used KMD algorithm is achieved.

Since in the system there are no frequencies larger than 10, it is enough to take $\Delta t = 0.03$ between neighboring snapshots. By using time-lagged embedding for each variable separately, we form the Hankel matrices and apply the GKP for the reconstruction and prediction. In the computations we present here, we use the training sets that consists of 900 snapshots and Hankel matrices of dimension 600×300 sliding along the computational domain with the chosen step. The length of the training sets was chosen so that at least two time periods of the global disturbance that we want to reveal are included in them.

The switching moment from resonant to normal breathing is nicely detected by eigenvalues provided by DMD algorithm in Figure S9. The nature of eigenvalues changes significantly in the active window beginning at $t = 150$ when switching of the dynamics occurs. It is nicely visible from Figures S9 – S10 that when the training set is in the zone of the resonant breathing, the GKP results are perfect in that same zone, and then deteriorate as we move into the normal breathing zone. This is as expected since after the moment of transition from the resonant to normal breathing the dynamical system is not governed by the same set of parameters. When the training set is in the zone of the normal breathing, the GKP in that zone is much less accurate then in the resonant breathing zone. It catches well the global behavior but it is poor in the details.

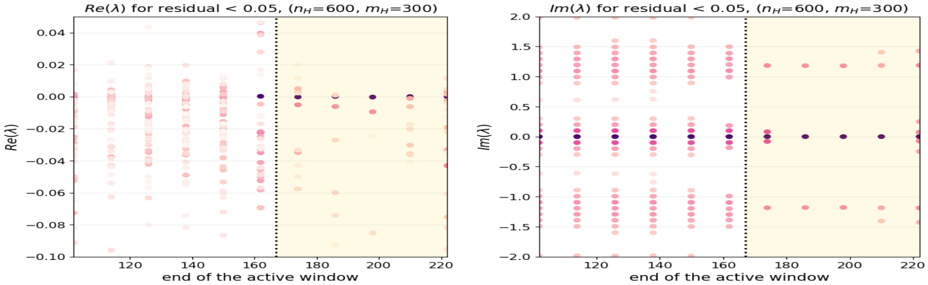

Figure S9. Physiology model. The real and the imaginary parts of eigenvalues for sliding active windows for which the residuals are smaller than the threshold $\eta_r = 0.025$. The intensity of color of eigenvalues is associated with the amplitude of modes.

What we can conclude from the presented results is the following. When the training set is in the zone of the resonant breathing, the GKP algorithm results are perfect in that same zone, and then deteriorate as we move into the normal breathing zone. This is as expected since after the moment of transition form the resonant to normal breathing the dynamical system is not governed by the same set of parameters. When the training set is in the zone of the normal breathing, the GKP in that same normal breathing zone is much less accurate then in the resonant breathing zone. It catches well the global behavior but it is poor in the

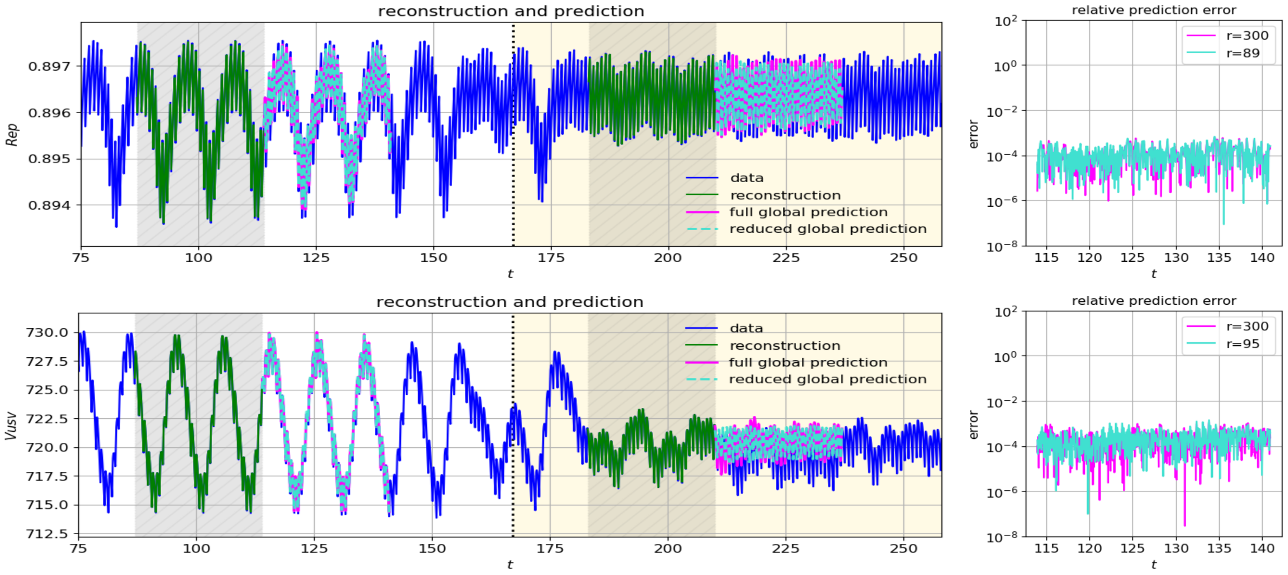

Figure S10. Physiology model. Extrasplanchnic peripheral resistance and splanchnic venous unstressed volume. First column: Reconstruction and prediction obtained using GKP for the chosen active windows. The full ($r = 300$) and reduced ($r < 300$) prediction obtained with GKP on active windows in the first part of simulation in the breathing zones captures accurately the dynamics, while even the full prediction obtained with the training set in the resonant breathing zone does not capture well the dynamics. Second column: The prediction errors for the full and reduced prediction (with r modes) for the chosen active window in the first part of simulation.

detail, most of the error value comes from the difference in the phase. This is also logical since normal breathing is much more irregular than the resonant breathing and it turns out that it can not be learned with high accuracy.

S2.3 Case study: Geomagnetic substorms

Geomagnetic storms and substorms are violent disturbances of the Earth's magnetosphere, caused by energy transfer of the solar wind into the planets magnetosphere, with potentially severe impact on the human civilization[74,75].

Physics-based modeling (see e.g.[76],[77],[78]) of geomagnetic substorms and storm/substorm interaction is a challenging task and the subject of intensive study. It must cover multiscale, nonlinear interactions of plasmas that are not in equilibrium, or are in an unstable equilibrium, which makes such modeling difficult to apply when prediction is needed, see e.g.[79].

On the other hand, given an abundance of observation data, a data-driven approach is an attractive alternative; see e.g.[80],[81]. The intensity of a substorm is quantified by the Auroral Electrojet (AE) index, the AL, which is a measure of the magnitude of the geomagnetic field disturbances on the ground induced by ionospheric currents developed during substorm. Other information such as e.g. solar wind data[82], the *Dst* index, and other substorm signature indices may be available[83] and used as observables.

For the purpose of this case study of the proposed approach as a purely data driven black-box methodology, we choose to use the AL index as the only observable; the data are downloaded from the Kyoto Geomagnetism Data Service (`http://wdc.kugi.kyoto-u.ac.jp/`).

The presented results are obtained by using global prediction algorithm with the active windows of size 30 and the Hankel matrices of dimension 20×10. By sliding the active windows along the computational domain we get prediction at different time moments. In Figure S11 we present the obtained reconstruction and prediction results for four active windows. Note that the modal representation of the signal is good, and it could provide a valuable insights to the experts in magnetic storm physics.

In the framework of our theory, the poor global prediction results are to be expected – almost all eigenvectors used in the KMD have large residuals. This once more justifies our approach, based on using the residuals of the Ritz pairs, computable even in the data driven setting, using the method from[12]. However, large prediction errors trigger the switch to the local prediction algorithm, which delivers more accurate predictions, at least for shorter lead time, as shown in Figure S11.

S2.4 Additional numerical results for prediction of influenza cases

Here we provide some additional results for the material in §2 of the paper. In Figures S12–S15 we show prediction of the dynamics of influenza for USA and UK for 2 and 52 weeks ahead, obtained with the KMD decompositions in the global

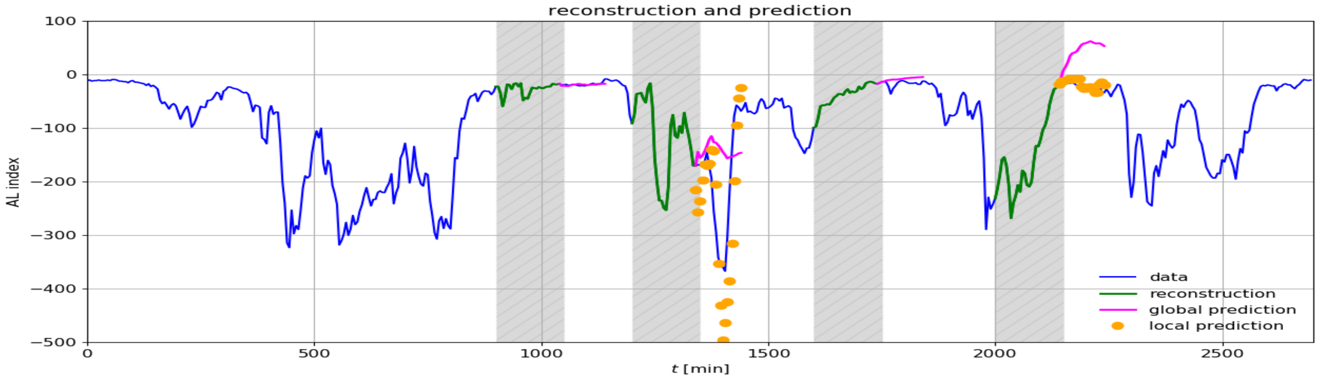

Figure S11. Geomagnetic substorms data: KMD reconstruction and prediction of the AL index. For reconstruction and global prediction, the data are collected in four active windows (time intervals $[900, 1050]$, $[1200, 1350]$, $[1600, 1750]$, $[2000, 2150]$, indicated by shadowed rectangles), the algorithm uses 20×10 Hankel matrices and then the dynamics is predicted for 20 time steps ahead. The time resolution of the collected data is $\delta t = 5$ min. The local prediction algorithm uses 3×2 matrices and the error threshold for resizing the Hankel matrix to minimal size (switching to the local algorithm) is set to 10. The dynamics with the local prediction algorithm is predicted two time steps ahead.

prediction algorithm, using sliding active windows of size 312.

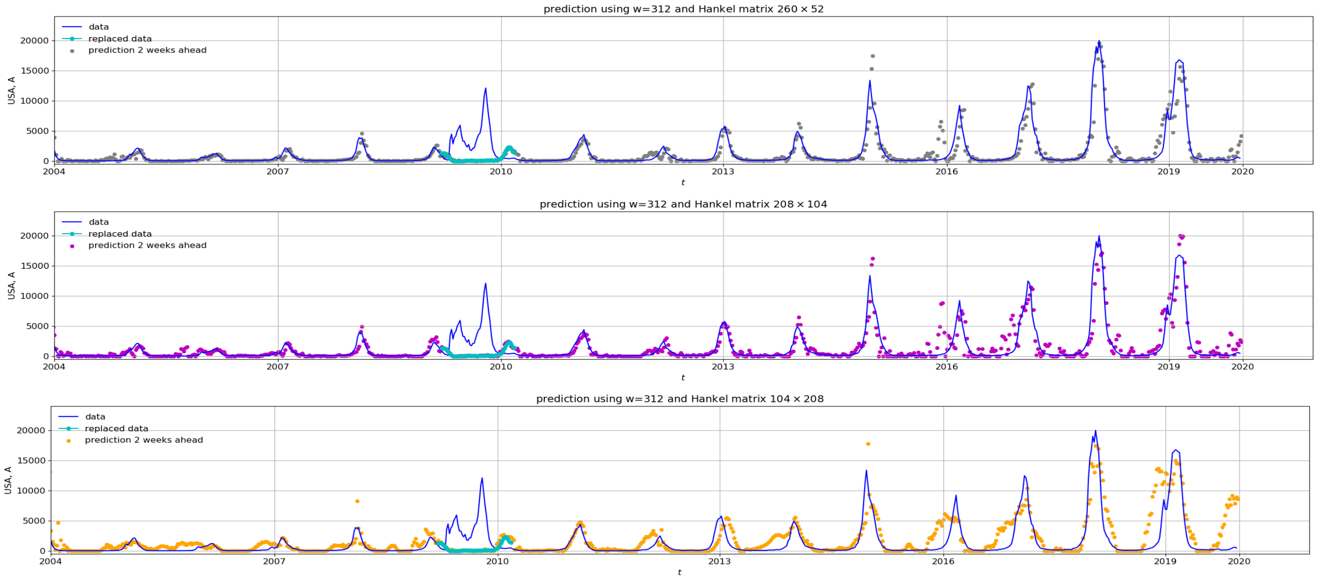

Figure S12. Influenza data (USA). Global Koopman prediction on influenza with the size of active windows 312 and different sizes of Hankel matrices. The prediction 2 weeks ahead by using KMD's from the active windows sliding along computational domain with sliding step $\Delta p = 1$.

In Figure S16, we provide additional numerical illustration (most relevant eigenvalues before and after retouching, with the corresponding prediction errors, and relation with the dominant frequencies from the DFT analysis) related to Figure 1b in the main paper.

References

1. Mezić, I. & Banaszuk, A. Comparison of systems with complex behavior. *Phys. D: Nonlinear Phenom.* **197**, 101–133 (2004).
2. Levnajić, Z. & Mezić, I. Ergodic theory and visualization. i. mesochronic plots for visualization of ergodic partition and invariant sets. *Chaos: An Interdiscip. J. Nonlinear Sci.* **20**, 033114 (2010).

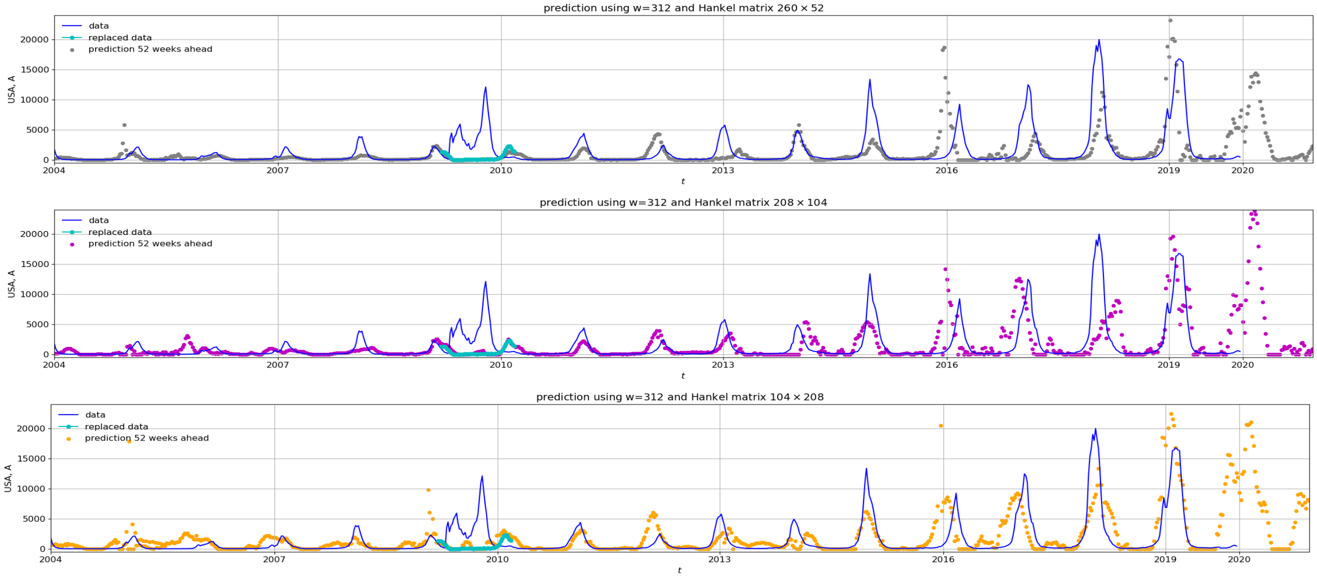

Figure S13. Influenza data (USA). Global Koopman prediction on influenza with the size of active windows 312 and different sizes of Hankel matrices. The prediction 52 weeks ahead by using KMD's from the active windows sliding along computational domain with sliding step $\Delta p = 1$.

3. Mohr, R. & Mezić, I. Construction of eigenfunctions for scalar-type operators via laplace averages with connections to the Koopman operator. *arXiv preprint arXiv:1403.6559* (2014).
4. Mezić, I. Spectrum of the Koopman operator, spectral expansions in functional spaces, and state-space geometry. *J. Nonlinear Sci.* 1–55 (2019).
5. Korda, M. & Mezić, I. On convergence of extended dynamic mode decomposition to the Koopman operator. *J. on Nonlinear Sci.* **28**, 687–10 (2018).
6. Das, S. & Yorke, J. A. Super convergence of ergodic averages for quasiperiodic orbits. *Nonlinearity* **31**, 491 (2018).
7. Arbabi, H. & Mezić, I. Ergodic theory, dynamic mode decomposition, and computation of spectral properties of the Koopman operator. *SIAM J. on Appl. Dyn. Syst.* **16**, 2096–2126 (2017).
8. Mezić, I. & Runolfsson, T. Uncertainty propagation in dynamical systems. *Automatica* **44**, 3003–3013 (2008).
9. Budišić, M., Mohr, R. & Mezić, I. Applied Koopmanism. *Chaos: An Interdiscip. J. Nonlinear Sci.* **22** (2012).
10. Susuki, Y., Mezic, I., Raak, F. & Hikihara, T. Applied Koopman operator theory for power systems technology. *Nonlinear Theory Its Appl. IEICE* **7**, 430–459, DOI: 10.1587/nolta.7.430 (2016).
11. Williams, M., Kevrekidis, I. & Rowley, C. A data-driven approximation of the Koopman operator: extending dynamic mode decomposition. *J. Nonlinear Sci.* **25**, 1307–1346 (2015).
12. Drmač, Z., Mezić, I. & Mohr, R. Data driven modal decompositions: Analysis and enhancements. *SIAM J. on Sci. Comput.* **40**, A2253–A2285, DOI: 10.1137/17M1144155 (2018). https://doi.org/10.1137/17M1144155.
13. Drmač, Z., Mezić, I. & Mohr, R. Data driven Koopman spectral analysis in Vandermonde–Cauchy form via the DFT: Numerical method and theoretical insights. *SIAM J. on Sci. Comput.* **41**, A3118–A3151, DOI: 10.1137/18M1227688 (2019). https://doi.org/10.1137/18M1227688.
14. Drmač, Z., Mezić, I. & Mohr, R. On least squares problems with certain Vandermonde-Khatri-Rao structure with applications to DMD. *SIAM J. on Sci. Comput.* **42**, A3250–A3284, DOI: 10.1137/19M1288474 (2020). https://doi.org/10.1137/19M1288474.
15. Taleb, N. N. *The Black Swan: The Impact of the Highly Improbable* (Random House Group, 2007).
16. Singh, R. K. & Manhas, J. S. *Composition operators on function spaces*, vol. 179 of *North-Holland Mathematics Studies* (North Holland, 1993).
17. Eisner, T., Farkas, B., Haase, M. & Nagel, R. *Operator theoretic aspects of ergodic theory*, vol. 272 of *Graduate Texts in Mathematics* (Springer, 2015).

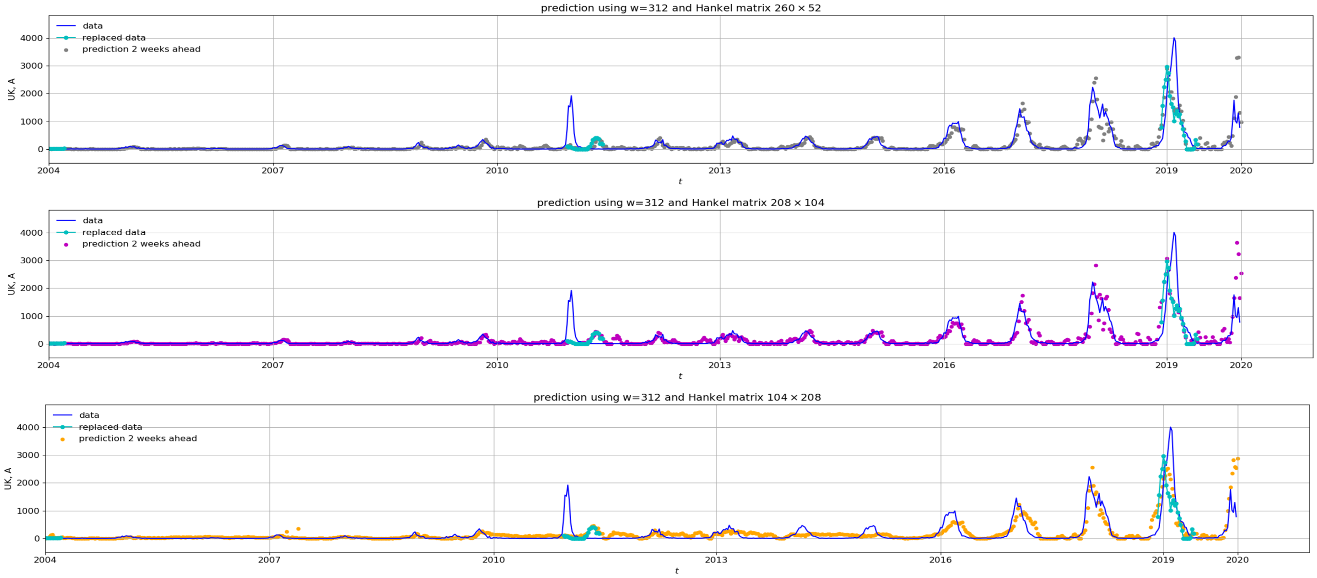

Figure S14. Influenza data (UK). Global Koopman prediction on influenza with the size of active windows 312 and different sizes of Hankel matrices. The prediction 2 weeks ahead by using KMD's from the active windows sliding along computational domain with sliding step $\Delta p = 1$.

18. Pilyugin, S. Y. Theory of pseudo-orbit shadowing in dynamical systems. *Differ. Equations* **47**, 1929–1938, DOI: 10.1134/S0012266111130040 (2011).

19. Pilyugin, S. *Shadowing in Dynamical Systems*, vol. 1706 of *Lecture Notes in Mathematics* (Springer, 1999).

20. Mezić, I. Spectral properties of dynamical systems, model reduction and decompositions. *Nonlinear Dyn.* **41**, 309–325 (2005).

21. Mezić, I. Spectrum of the Koopman operator, spectral expansions in functional spaces, and state-space geometry. *J. Nonlinear Sci.* 1–55 (2019).

22. Mezić, I. Spectrum of the Koopman operator, spectral expansions in functional spaces, and state-space geometry. *J. Nonlinear Sci.* DOI: 10.1007/s00332-019-09598-5 (2019).

23. Giannakis, D. Data-driven spectral decomposition and forecasting of ergodic dynamical systems. *Appl. Comput. Harmon. Analysis* **47**, 338 – 396, DOI: https://doi.org/10.1016/j.acha.2017.09.001 (2019).

24. Govindarajan, N., Mohr, R., Chandrasekaran, S. & Mezićc, I. On the approximation of Koopman spectra for measure preserving transformations. *SIAM J. on Appl. Dyn. Syst.* **18**, 1454–1497, DOI: 10.1137/18M1175094 (2019). https://doi.org/10.1137/18M1175094.

25. Ghosal, S., Ramanan, V., Sarkar, S., Chakravarthy, S. & Sarkar, S. Detection and analysis of combustion instability from hi-speed flame images using dynamic mode decomposition. In *ASME. Dynamic Systems and Control Conference, Volume 1*, DOI: 10.1115/DSCC2016-9907 (2016).

26. Tu, J. H., Rowley, C. W., Luchtenburg, D. M., Brunton, S. L. & Kutz, J. N. On dynamic mode decomposition: Theory and applications. *J. Comput. Dyn.* **1**, 391–421, DOI: 10.3934/jcd.2014.1.391 (2014).

27. Drmač, Z., Mezić, I. & Mohr, R. Identification of nonlinear systems using the infinitesimal generator of the koopman semigroup – a numerical implementation of the mauroy-goncalves method. *Mathematics* **9**, 2075, DOI: 10.3390/math9172075 (2021).

28. Schmid, P. Dynamic mode decomposition of numerical and experimental data. *J. Fluid Mech.* **656**, 5–28 (2010).

29. Pearson, K. On lines and planes of closest fit to systems of points in space. *Philos. Mag.* **2**, 559–572 (1901).

30. Eckart, C. & Young, G. The approximation of one matrix by another of lower rank. *Psychometrika* **1**, 211–218, DOI: 10.1007/BF02288367 (1936).

31. Mirsky, L. Symmetric gauge functions and unitarily invariant norms. *The Q. J. Math.* **11**, 50, DOI: 10.1093/qmath/11.1.50 (1960).

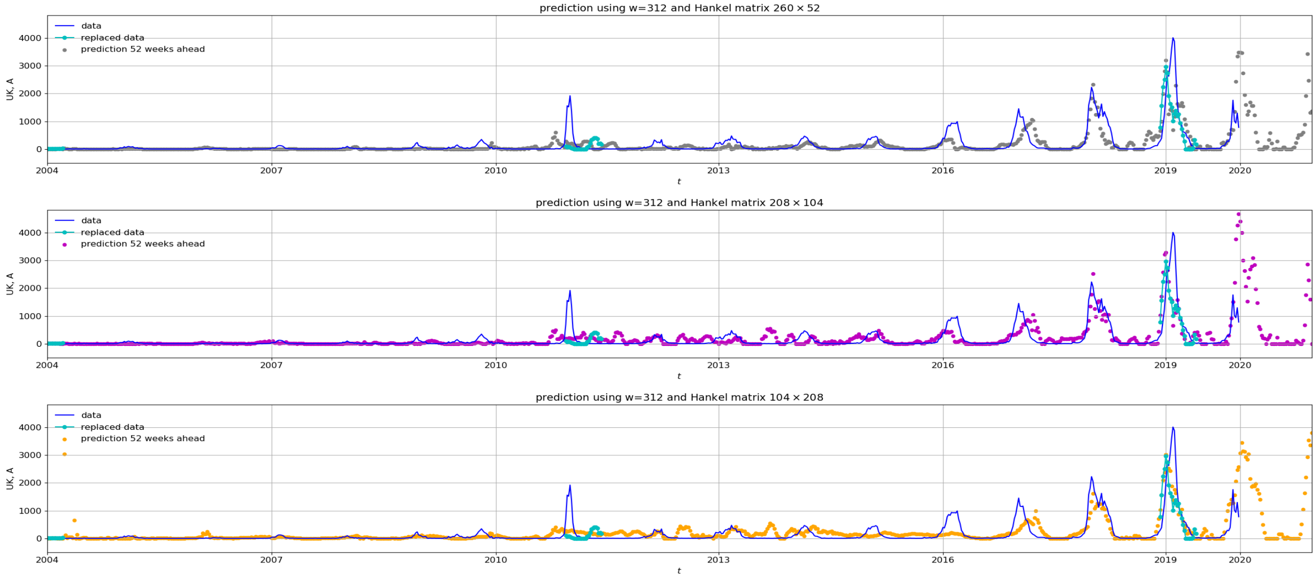

Figure S15. Influenza data (UK). Global Koopman prediction on influenza with the size of active windows 312 and different sizes of Hankel matrices. The prediction 52 weeks ahead by using KMD's from the active windows sliding along computational domain with sliding step $\Delta p = 1$.

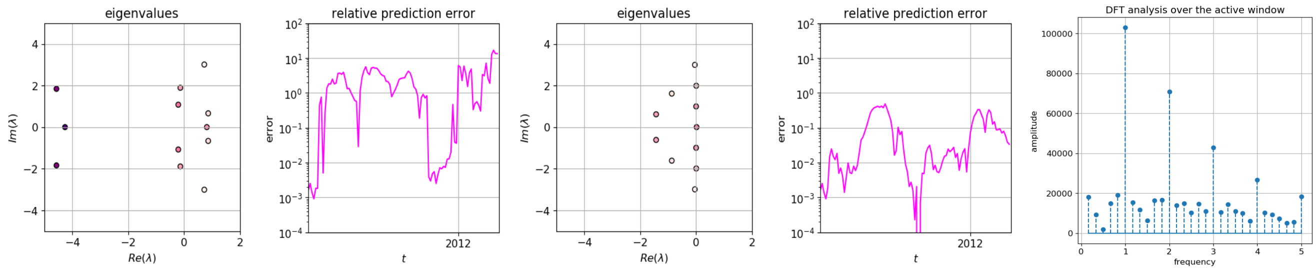

(a) Before retouching **(b)** After retouching **(c)** DFT analysis.

Figure S16. Influenza data (USA). The most relevant eigenvalues and the prediction errors in the global algorithm (using 208×104 Hankel matrices) for the active window as in Figure 1b in the main paper. Note how the unstable eigenvalues ($\Re(\lambda) > 0$) impact the prediction performance, and how the retouching moves them to the left. Compare with Figures 1b and 2 in the main paper. Note how the dominant frequencies from the DFT analysis correspond to the imaginary parts of the eigenvalues computed after the retouching and selected by the residual criterion.

32. Golub, G. H., Klema, V. C. & Stewart, G. W. Rank degeneracy and least squares problems. Tech. Rep. CS-TR-76-559, STANFORD UNIV CA DEPT OF COMPUTER SCIENCE, Stanford, CA, USA (1976).

33. Taira, K. *et al.* Modal analysis of fluid flows: An overview. *AIAA J.* **55**, 4013–4041 (2017).

34. Chen, K. K., Tu, J. H. & Rowley, C. W. Variants of dynamic mode decomposition: Boundary condition, Koopman, and Fourier analyses. *J. Nonlinear Sci.* **22**, 887–915 (2012).

35. Hemati, M. S., Rowley, C. W., Deem, E. A. & Cattafesta, L. N. De-biasing the dynamic mode decomposition for applied Koopman spectral analysis. *ArXiv e-prints* (2015). 1502.03854.

36. Dawson, S. T. M., Hemati, M. S., Williams, M. O. & Rowley, C. W. Characterizing and correcting for the effect of sensor noise in the dynamic mode decomposition. *Exp. Fluids* **57**, 42, DOI: 10.1007/s00348-016-2127-7 (2016).

37. Hemati, M. S., Williams, M. O. & Rowley, C. W. Dynamic mode decomposition for large and streaming datasets. *Phys. Fluids* **26**, 111701 (2014).

38. Takeishi, N., Kawahara, Y. & Yairi, T. Subspace dynamic mode decomposition for stochastic Koopman analysis. *Phys. Rev. E* **96**, 033310, DOI: 10.1103/PhysRevE.96.033310 (2017).

39. Takeishi, N., Kawahara, Y., Tabei, Y. & Yairi, T. Bayesian dynamic mode decomposition. In *Proceedings of the Twenty-Sixth International Joint Conference on Artificial Intelligence, IJCAI-17*, 2814–2821, DOI: 10.24963/ijcai.2017/392 (2017).

40. Takeishi, N., Kawahara, Y. & Yairi, T. Sparse nonnegative dynamic mode decomposition. In *2017 IEEE International Conference on Image Processing (ICIP)*, 2682–2686, DOI: 10.1109/ICIP.2017.8296769 (2017).

41. Takeishi, N., Kawahara, Y. & Yairi, T. Learning Koopman invariant subspaces for dynamic mode decomposition. In Guyon, I. *et al.* (eds.) *Advances in Neural Information Processing Systems 30*, 1130–1140 (Curran Associates, Inc., 2017).

42. Proctor, J., Brunton, S. & Kutz, J. Dynamic mode decomposition with control. *SIAM J. on Appl. Dyn. Syst.* **15**, 142–161, DOI: 10.1137/15M1013857 (2016). https://doi.org/10.1137/15M1013857.

43. Askham, T. & Kutz, J. Variable projection methods for an optimized dynamic mode decomposition. *SIAM J. on Appl. Dyn. Syst.* **17**, 380–416, DOI: 10.1137/M1124176 (2018). https://doi.org/10.1137/M1124176.

44. Jovanović, M. R., Schmid, P. J. & Nichols, J. W. Sparsity-promoting dynamic mode decomposition. *Phys. Fluids* **26**, 024103 (2014).

45. Tu, J. H., Rowley, C. W., Luchtenburg, D. M., Brunton, S. L. & Kutz, J. N. On dynamic mode decomposition: theory and applications. *J. Comput. Dyn.* **1**, 391–421 (2014).

46. Arbabi, H. & Mezić, I. Ergodic theory, dynamic mode decomposition and computation of spectral properties of the koopman operator. *ArXiv e-prints* (2016). 1611.06664.

47. Drmač, Z., Mezić, I. & Mohr, R. Data driven modal decompositions: Analysis and enhancements. *SIAM J. on Sci. Comput.* **40**, A2253–A2285, DOI: 10.1137/17M1144155 (2018). https://doi.org/10.1137/17M1144155.

48. Drmač, Z., Mezić, I. & Mohr, R. Identification of nonlinear systems using the infinitesimal generator of the koopman semigroup - a numerical implementation of the mauroy-goncalves method. *Mathematics* **9**, DOI: 10.3390/math9172075 (2021).

49. Drmač, Z. A lapack implementation of the dynamic mode decomposition i. Tech. Rep., Department of Mathematics, University of Zagreb, Croatia, and AIMdyn Inc. Santa Barbara, CA (2022). LAPACK Working Note 298.

50. Drmač, Z. A lapack implementation of the dynamic mode decomposition ii. Tech. Rep., Department of Mathematics, University of Zagreb, Croatia, and AIMdyn Inc. Santa Barbara, CA (2022). LAPACK Working Note 300.

51. Drmač, Z. A lapack implementation of the Dynamic Mode Decomposition. *ACM Trans. Math. Soft. (in revision)* (2023).

52. Drmač, Z. Hermitian dynamic mode decomposition – numerical analysis and software solution. *ACM Trans. Math. Soft. (in revision)* (2023).

53. Hale, T., Webster, S., Petherick, A., Phillips, T. & Kira, B. Oxford COVID-19 government response tracker. Tech. Rep., Blavatnik School of Government (2020).

54. Petherick, A. *et al.* Variation in government responses to COVID-19. Tech. Rep. BSG-WP-2020/032, Blavatnik School of Government (2020).

55. Gostic, K. M. *et al.* Practical considerations for measuring the effective reproductive number, r_t. *PLoS computational biology* **16**, e1008409 (2020).

56. Metzner, P., Putzig, L. & Horenko, I. Analysis of persistent nonstationary time series and applications. *Commun. Appl. Math. Comput. Sci.* **7**, 175–229, DOI: 10.2140/camcos.2012.7.175 (2012).

57. Lorenz, E. N. Deterministic nonperiodic flow. *J. atmospheric sciences* **20**, 130–141 (1963).

58. Brunton, S. L., Brunton, B. W., Proctor, J. L., Kaiser, E. & Kutz, J. N. Chaos as an intermittently forced linear system. *Nat. communications* **8**, 1–9 (2017).

59. Korda, M., Putinar, M. & Mezić, I. Data-driven spectral analysis of the Koopman operator. *Appl. Comput. Harmon. Analysis* **48**, 599–629 (2020).

60. Giannakis, D., Das, S. & Slawinska, J. Reproducing kernel hilbert space compactification of unitary evolution groups. *arXiv preprint arXiv:1808.01515* (2018).

61. Iooss, G. & Joseph, D. D. *Elementary stability and bifurcation theory* (Springer Science & Business Media, 2012).

62. Luzzatto, S., Melbourne, I. & Paccaut, F. The Lorenz attractor is mixing. *Commun. Math. Phys.* **260**, 393–401 (2005).

63. Pathak, J., Lu, Z., Hunt, B. R., Girvan, M. & Ott, E. Using machine learning to replicate chaotic attractors and calculate lyapunov exponents from data. *Chaos: An Interdiscip. J. Nonlinear Sci.* **27**, 121102 (2017).

64. Magosso, E. & Ursino, M. Cardiovascular response to dynamic aerobic exercise: A methematical model. *Med. Biol. Eng. Comput.* **40**, 660–674 (2002).

65. Ursino, M. Interaction between carotid baroregulation and the pulsating heart: a mathematical model. *Am. J. Physiol. Circ. Physiol.* **275**, H1733–H1747 (1998).

66. Ursino, M. & Magosso, E. Acute cardiovascular response to isocapnic hypoxia. i. a mathematical model. *Am. J. Physiol. Circ. Physiol.* **279**, H149–H165 (2000).

67. Ursino, M. & Magosso, E. Role of short-term cardiovascular regulation in heart period variability: a modeling study. *Am. J. Physiol. Circ. Physiol.* **53**, H1479 (2003).

68. Stefanovska, A. Physics of the human cardiovascular system. *Contemp. Phys.* **40**, 31–55 (1999).

69. Fonoberova, M. *et al.* A computational physiology approach to personalized treatment models: the beneficial effects of slow breathing on the human cardiovascular system. *Am. J. Physiol. Circ. Physiol.* **307**, H1073–H1091 (2014).

70. Lehrer, P. M. *et al.* Heart rate variability biofeedback increases baroreflex gain and peak expiratory flow. *Psychosom. medicine* **65**, 796–805 (2003).

71. Lin, G. *et al.* Heart rate variability biofeedback decreases blood pressure in prehypertensive subjects by improving autonomic function and baroreflex. *The J. Altern. Complementary Medicine* **18**, 143–152 (2012).

72. Vaschillo, E., Lehrer, P., Rishe, N. & Konstantinov, M. Heart rate variability biofeedback as a method for assessing baroreflex function: a preliminary study of resonance in the cardiovascular system. *Appl. psychophysiology biofeedback* **27**, 1–27 (2002).

73. Vaschillo, E. G., Vaschillo, B., Buckman, J. F., Pandina, R. J. & Bates, M. E. Measurement of vascular tone and stroke volume baroreflex gain. *Psychophysiology* **49**, 193–197 (2012).

74. Space Weather Prediction Center, National Oceanic and Atmospheric Administration. https://www.swpc.noaa.gov/. Accessed: 2020-01-04.

75. Hapgood, M. The great storm of may 1921: An exemplar of a dangerous space weather event. *Space Weather.* **17**, 950–975 (2019).

76. Spencer, E., Vadepu, S. K., Srinivas, P., Patra, S. & Horton, W. The dynamics of geomagnetic substorms with the WINDMI model. *Earth, Planets Space* **70** (2018).

77. Sharma, A. S., Kamide, Y. & (Eds.), G. S. L. *Disturbances in Geospace: The Storm-Substorm Relationship*, vol. 142 of *Geophysical Monograph Series* (American Geophysical Union, 2003).

78. Kamide, Y. *et al.* Current understanding of magnetic storms: Storm-substorm relationships. *J. Geophys. Res. Space Phys.* **103**, 17705–17728, DOI: 10.1029/98JA01426 (1998).

79. Morley, S. K., Freeman, M. P., & Tanskanen, E. I. A comparison of the probability distribution of observed substorm magnitude with that predicted by a minimal substorm model. *Ann. Geophys.* **25**, 2427–2437 (2007).

80. Giannakis, D., Gkioulidou, M. & Harlim, J. Probabilistic prediction of the AL index with the diffusion forecasting model. *arXiv e-prints* arXiv:1612.07272 (2016). 1612.07272.

81. Maimaiti, M., Kunduri, B., Ruohoniemi, J. M., Baker, J. B. H. & House, L. L. A deep learning-based approach to forecast the onset of magnetic substorms. *Space Weather.* **17** (2019).

82. Newell, P. *et al.* Substorm probabilities are best predicted from solar wind speed. *J. Atmospheric Solar-Terrestrial Phys.* **146**, 28 – 37, DOI: https://doi.org/10.1016/j.jastp.2016.04.019 (2016).

83. Nosé, M. *et al.* Wp index: A new substorm index derived from high-resolution geomagnetic field data at low latitude. *Space Weather.* **10**, DOI: 10.1029/2012SW000785 (2012).
